# A Viral Neuraminidase-Specific Sensor for Taste-Based Detection of Influenza

**DOI:** 10.1021/acscentsci.5c01179

**Published:** 2025-10-01

**Authors:** Martina Raschig, Marcus Gutmann, Josef Kehrein, Eberhard Heller, Michael Bomblies, Marcel Groß, Oskar Steinlein, Peggy Riese, Stephanie Trittel, Tessa Lühmann, Carlos A. Guzmán, Jürgen Seibel, Heinrich Jehle, Christian Linz, Stephan Hackenberg, Lorenz Meinel

**Affiliations:** † Institute of Pharmacy and Food Chemistry, University of Würzburg, Am Hubland, 97074 Würzburg, Germany; ‡ Institute of Organic Chemistry, University of Würzburg, Am Hubland, 97074 Würzburg, Germany; § Bettackerst. 17, 72636 Frickenhausen, Germany; ∥ Faculty of Medicine and University Hospital Cologne, Department of Oral, Maxillofacial and Plastic Surgery, 14309 University of Cologne, Kerpener Str. 62, 50937 Cologne, Germany; ⊥ Department of Otorhinolaryngology, Plastic, Aesthetic and Reconstructive Head and Neck Surgery, 27207 University Hospital Würzburg, 97080 Würzburg, Germany; # Department Vaccinology and Applied Microbiology (VAC), Helmholtz Centre for Infection Research (HZI), 38124 Braunschweig, Germany; g Helmholtz Institute for RNA-Based Infection Research (HIRI), 28336 Helmholtz Center for Infection Research (HZI), 97080 Würzburg, Germany

## Abstract

Influenza has caused the deadliest pandemics in history, thereby prompting advances in our ability to ensure vigilance at all stages of future outbreaks. Quarantining patients early is crucial when it comes to preventing these outbreaks, but it is challenging with influenza due to presymptomatic transmission. Presymptomatic detection translates into massive screening needs, which necessitate cost-effective tools with access for anyone, anywhere, and at any time. We met these challenges by synthesizing sensors that respond to influenza infections with taste generation by using the tongue as an always-available detector. In doing so, we utilized the virus’s need for neuraminidase cleavage of α-glycosidic bonds to detect its presence in patients. We synthesized *N*-acetylneuraminic acid-thymol derivatives and chemically tuned them to respond to viral but not bacterial neuraminidase. Viral selectivity was further confirmed via structural analysis and molecular docking. Influenza sensors that respond to viral presence with taste may have unmatched advantages regarding accessibility and cost-effectiveness, including the potential to first-line stratify millions of healthy individuals from flu patients, thereby enabling us to leverage our response armamentarium in future outbreaks.

## Graphical abstract

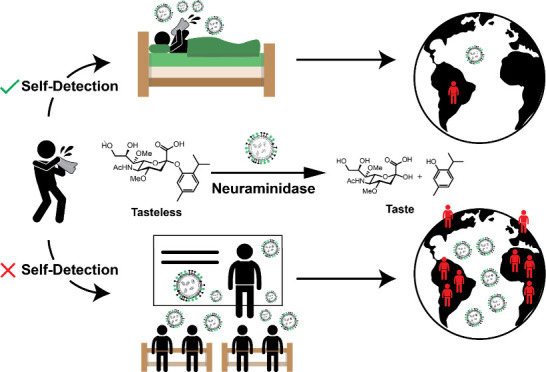

## Introduction

Influenza causes acute respiratory diseases, with an estimated death toll of about 500,000 individuals every year.^1,2^ The predominant circulating types A and B can be further subdivided: Type B is categorized into lineages B/Victoria/2/87 and B/Yamagata/16/88, and type A (HxNy) is categorized into subtypes based on the surface enzymes hemagglutinin (H) and neuraminidase (N).^1,3–7^ Although influenza infections declined in the 2020/2021 season due to measures against the COVID-19 pandemic, the risk of circulating influenza viruses should not be underestimated.^8,9^

Apocalyptic outbreaks include the “Spanish flu” (1918–1920), with subsequent waves of the H1N1 virus affecting more than one-fourth of the global population, thereby making it one of the deadliest pandemics in history.^10–14^ H3N2—which hit globally in 1968—continues to circulate today.^14–17^ Influenza spreads from animals, as with bird flu. Between 2022 and 2024, zoonoses were reported from dairy cows, poultry, and unknown animal exposure in the US and were mostly identified as H5N1.^18^ Moreover, avian influenza spread directly from poultry to humans in 1997,^19^ and swine influenza viruses have caused sporadic human infections.^6^ Additionally, 2009 saw another H1N1 pandemic: This time, more than 80% of deaths occurred in people younger than 65—a striking difference from typical seasonal influenza epidemics.^20–23^ Consequently, the US government developed a “National Strategy for Pandemic Influenza Implementation Plan” that includes diagnostic devices for stratifying patients affected by influenza or secondary bacterial infections.^19,24,25^

If an upcoming influenza pandemic hits again, we must be prepared to screen regions of virtually unlimited size instantaneously, be they cities, states, or entire continents.^26–29^ Diagnostics with high accuracy—such as PCR—offer satisfying sensitivity (i.e., they correctly identify those with the infection) and specificity (i.e., they correctly identify those without the infection). However, they are slow, and their widespread use is logistically challenging or overly expensive, thereby blocking effective responses to pandemics, particularly in low-income countries.^26^ As demonstrated during the COVID-19 pandemic, serological tests are hardly accurate in early, asymptomatic phases, with the best sensitivity appearing during the first week after symptom onset.^30^ Therefore, a significant gap exists in current approaches to influenza infections, which renders these approaches potentially less effective. Many available methods either are too complex for widespread use or fail to accurately identify the presymptomatic stages when influenza can spread effectively. Thus, there is an urgent need for readily manufacturable, easily supplied, and straightforward first-line defense tools. These tools could quickly help identify individuals at risk of carrying influenza so that they can be moved into quarantine. This initial step could then be followed by more accurate—albeit slower and more expensive—confirmatory tests. We therefore set out to create a flu-testing framework that is rapidly accessible, cheap to produce, easy to distribute, and responsive in the early phases of infection as a prerequisite for global use. We solved this challenge by switching away from complex detectors and machinery and toward a detector that is available for anyone, everywhere, and anytime: the tongue. We thus chemically deployed a crucial step in viral replication: the requirement of neuraminidase to cleave α-glycosidic bonds. We synthesized alkylated *N*-acetylneuraminic acids and glycosidically linked a model taste molecule: the monoterpene thymol. The *N*-acetylneuraminic acid-based sensor molecule has been chemically modified to specifically respond to viral but not bacterial neuraminidase at concentrations found in the saliva of patients with active influenza.^31–37^ In these patients, the synthesized molecules—or sensors—exploit the virus’s essential need for neuraminidase to release the infectious agent from host cells. In the final chemical design, viral—but not bacterial—neuraminidase cleaves off thymol from the molecule in human saliva. Future designs might integrate these sensors into chewing gum or thin film, and once an individual tastes thymol, measures regarding isolation and further confirmation can be taken by anyone instantaneously (Figure 1).^28,32,38–41^

**Figure 1:**
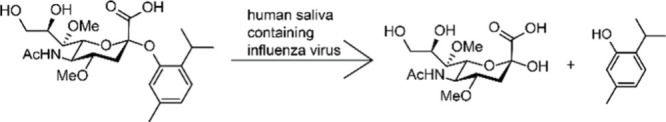
Principle of taste-based influenza detection and structure of the *N*-acetylneuraminic acid sensor.

## Results and Discussion

### Design and Synthesis of Sensors for Self-Diagnosing Influenza Infections

We synthesized and tested two *N*-acetylneuraminic acid sensors (Figure 2, S1–S3, S23–S44):^31–35,42–44^ (i) thymol that was *O*-glycosidically linked to unmodified *N*-acetylneuraminic acid, which is referred to as “the unmethylated reference sensor” (**6**), and (ii) thymol that was *O*-glycosidically linked to 4,7-di-*O*-methyl-*N*-acetylneuraminic acid, which is referred to as sensor (**15**). Starting from *N*-acetylneuraminic acid, 4,7-di-*O*-methyl-*N*-acetylneuraminic acid (**10**) was synthesized using modified protocols (Figure S3A).^32,33,43^ After acetylation of the hydroxyl groups of compounds (**11**) and (**2**), respectively, the anomeric position was chlorinated and hydrolyzed under aqueous conditions. The subsequent glycosylation of thymol was performed by a Mitsunobu reaction, which may be superior to the Koenigs–Knorr reaction for aromatic compounds.^32,33,42,45–52^ Deprotection of compounds (**5**) and (**14**) via sodium methoxide and lithium hydroxide resulted in (un)­methylated sensors (**6**) and (**15**) (Figure 2A).^45,53,54^ To test for improved time efficiency in large-scale productions, compound (**14**) was deprotected without purification, and only the final sensor (**15**) was purified. Three approaches were tested for coupling thymol to the modified *N*-acetylneuraminic acid backbone and subsequent deprotection: (i) To simplify the purification of compound (**14**), the polymer-supported reagent PPh_3_ was used, as PPh_3_O can then be separated directly by filtration. Reaction conversion at room temperature after 24 h was insufficient with lower diethyl azodicarboxylate (DEAD) amounts (1–1.3 equiv), which we increased to 3 equiv, thereby resulting in an additional byproduct (Figure S39). This byproduct complicated the purification and reduced the yield. Furthermore, the β-anomer of the sugar could not be isolated in this approach because the byproduct was coeluted during medium-performance liquid chromatography. This led to a modified synthesis approach (ii) using 1–1.3 equiv of DEAD but with dissolved PPh_3_, resulting in yield improvements and the synthesis of the pure β-sensor (**15**). This synthesis strategy also allows omitting the purification of compound (**14**), with potential advantages in effective and large-scale production of the α-sensor (**15**) (Figure 2A, ‘Synthetic Procedures’ section in the Supporting Information). To avoid reaction steps with unstable intermediates (compound (**13-1**)), we developed a third (iii) synthesis route (Figures 2B, S43, S44). The hydroxy groups at positions 8 and 9 of compound (**11**) were protected for that. The anomeric hydroxy group, required for the subsequent Mitsunobu reaction, remained unprotected, so that thymol could be coupled directly. After final deprotection, sensor (**15**) was obtained. Synthesis route (iii) requires two steps less than (ii) and reduces the handling of labile intermediates (Figure 2B).

**Figure 2:**
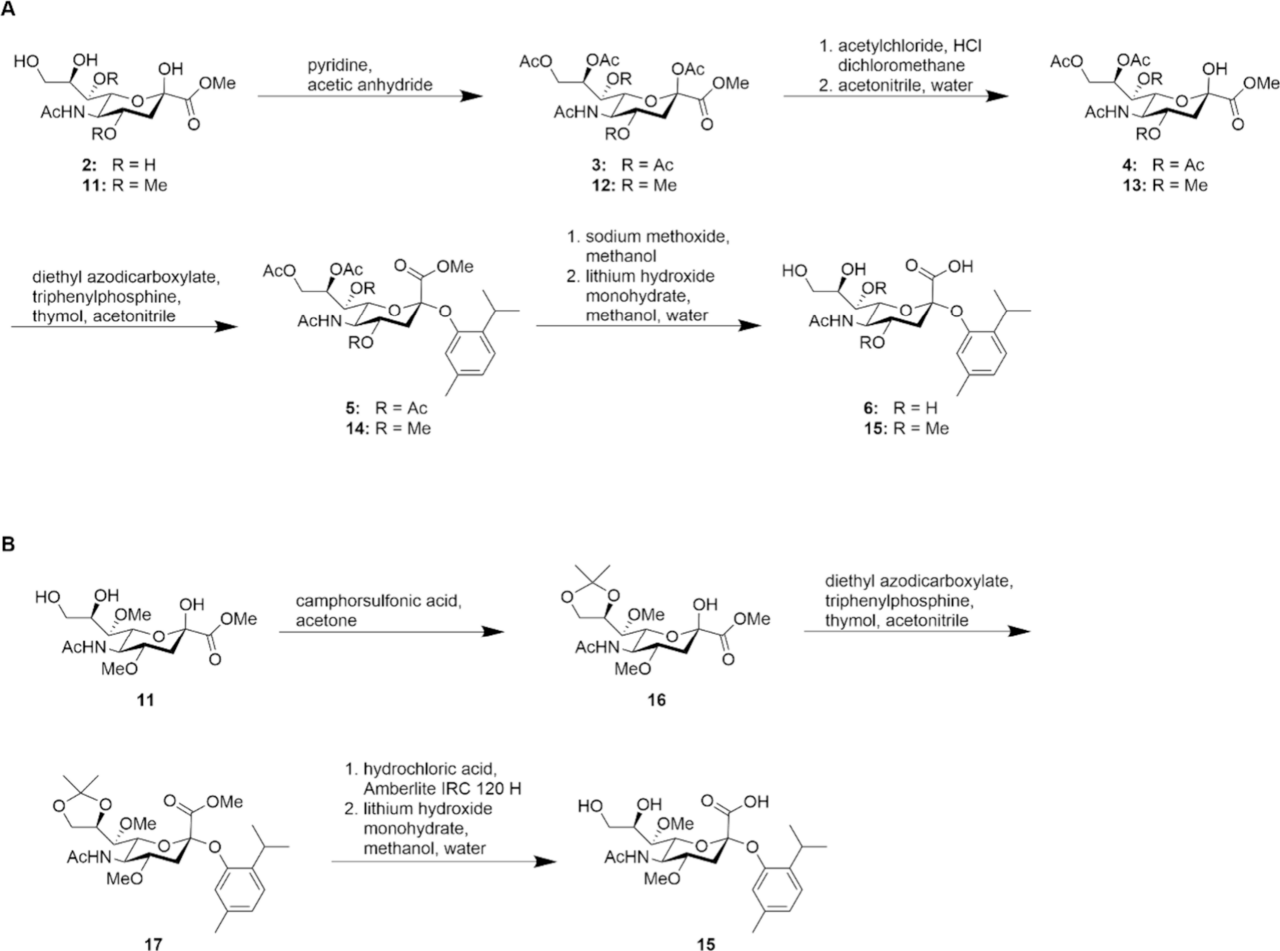
(A) Synthesis of thymol linked α-glycosidically to *N*-acetylneuraminic acid (unmethylated reference sensor (**6**)) or α-glycosidically to 4,7-di-*O*-methyl-*N*-acetylneuraminic acid (sensor (**15**); the scheme is again shown in Figure S1). (B) Optimized synthesis of thymol linked α-glycosidically to 4,7-di-*O*-methyl-*N*-acetylneuraminic acid.

It was unnecessary to separate the anomers in the precursor synthesis because the separation of the desired sensor into α-anomers (elution time for the α-sensor (**15**) *t* = 5.07 min) and β-anomers (elution time for the β-sensor (**15**) *t* = 4.90 min) via reverse-phase column chromatography was successful (see the ‘Synthetic Procedures’ section in the Supporting Information; Figures S6, S8). Conformations of the α- and β-anomers were confirmed by ^1^H NMR, ^13^C NMR (Figures S40, S41), and HMBC NMR (Figure S42). Moreover, menthol was conjugated to (un)­modified *N*-acetylneuraminic acid using the Koenigs–Knorr reaction in order to expand the chemical space from aromatic taste molecules (thymol) to saturated molecules (menthol; Figure S2).

The α- and ß-anomers of sensor (**15**) were evaluated for cytotoxicity using HEK 293 (human kidney cell line) and NIH 3T3 cells (mouse fibroblast cell line) (Figure S4). Both anomers showed no reduction in cell viability at concentrations up to 1.0 mM (>90% viability), indicating that these compounds are not cytotoxic (Figure S4).^55^

The stability of the α-sensor (**15**) was evaluated for 4 weeks under storage and stressed conditions: −20 °C, 4 °C, and 25 °C at 60% relative humidity (rh), and 50 °C at 75% rh. All storage conditions resulted in at least 95% stable α-sensor (**15**), except stressed conditions at 50 °C (94% stable; Figure S5).

### Tracking the Seasonal Dependency of Neuraminidase Activity in Human Saliva

The synthesized sensors were studied for their specificity to recombinant Influenza A virus H1N1 neuraminidase (viral neuraminidase) and recombinant *M. viridifaciens* neuraminidase (Kroppenstedt 2005,^56^ bacterial neuraminidase) in phosphate-buffered saline (PBS), *M. viridifaciens* buffer (50 mM sodium acetate, 150 mM NaCl, pH 4.5), and saliva. Bacterial neuraminidase was more effective than viral neuraminidase in cleaving glycosidic bonds (Table S1). In order to define clinically relevant neuraminidase activities in the saliva from PCR-positive influenza patients, we collected saliva in hospitalized, late-stage patients, reflecting influenza infections at ∼4–7 days postinfection during two seasons (2017/2018 and 2022/2023; Figure 3A; Supporting Information, section ‘Collection of Saliva Samples’). Neuraminidase activity was 8.9 ± 6.5 mU/mL (*n* = 16, median = 7.8 mU/mL) and 13.4 ± 8.3 mU/mL (*n* = 18, median = 13.1 mU/mL) and was not significantly different between the 2017/2018 and 2022/2023 seasons. Since all samples were taken from hospitalized individuals at different times, the observed scattering around the median reflects the overall variation in neuraminidase activities throughout the disease.^57,58^ As already mentioned, high concentrations are to be expected during the initial stage of the disease, which coincides with the intended utilization of our sensor as an indicator.^59^

### Sensor Selectivity Toward Viral Neuraminidase

Reflecting neuraminidase activities measured in influenza patient saliva, we used neuraminidase activities of 5–10 mU/mL for sensor analyses. We initially tested the commercially available neuraminidase sensor 4-MUNANA (4-methylumbelliferyl-*N*-acetyl-α-d-neuraminic acid),^60,61^ an unspecific viral and bacterial neuraminidase substrate. The unspecificity of 4-MUNANA results from the unmethylated neuraminic acid backbone at positions O4 and O7, similar to what is seen with our unmethylated reference sensor (**6**). With different concentrations of H1N1 (A/California/7/2009) foci forming units/mL (ffu/mL), 4-MUNANA (used at 4 mM; incubation time 10–30 min) was effectively cleaved, resulting in the cleavage product 4-methylumbelliferone (4MU) with concentrations of 0.04–0.056 mM at 10^4^ and 0.20–0.44 mM at 10^5^ ffu/mL (Figures 3B, S18A). To link these cleavage outcomes to neuraminidase activity, we used 4-MUNANA (4 mM) with viral neuraminidase of known activity (Table S1), translating into ∼4.1 ± 1.0 mU/mL at 10^4^ and ∼55.5 ± 9.0 mU/mL at 10^5^ ffu/mL (Table S2). As mentioned, these activities approximated those measured in patient saliva, establishing the clinical relevance of the neuraminidase activity space in which our experiments were performed (Figure 3A).

**Figure 3:**
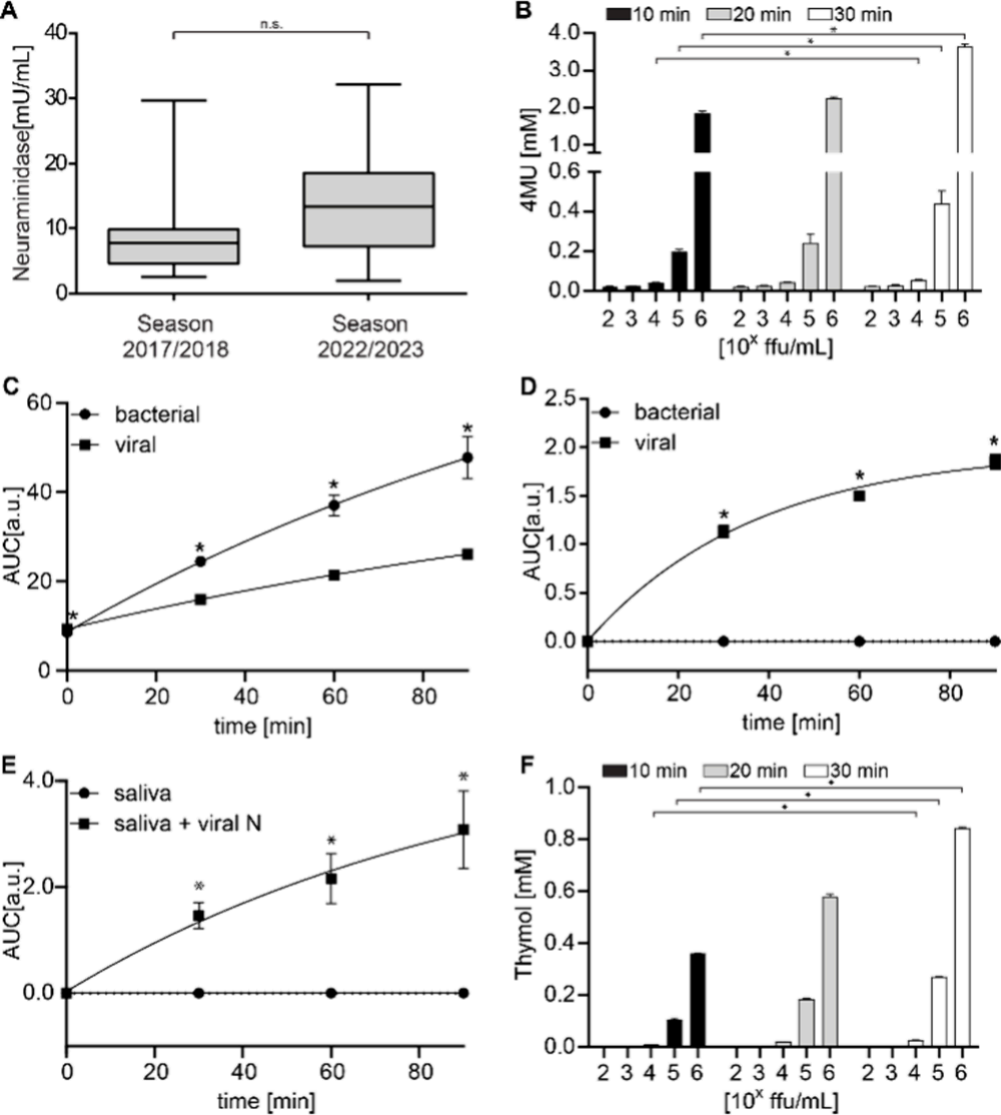
(A) Neuraminidase concentration in saliva from PCR-positive influenza patients. Values were calculated using a standard curve for which a sigmoidal (4PL) fit was used. A Mann–Whitney U test (*p* < 0.05) showed no significant difference across seasons. (B) Different concentrations of H1N1 (A/California/7/2009) incubated with 4-MUNANA for 10–30 min, resulting in the release of 4-methylumbelliferone (4MU) (mean ± SD, *n* = 3; Dixon outlier test followed by the Kruskal–Wallis test and Dunn posthoc test; *p* ≤ 0.05 (*) was considered statistically significant). (C) Selectivity of the unmethylated reference (**6**) against viral and bacterial neuraminidase (mean ± SD, *n* = 3; Dixon outlier test followed by multiple *t*-tests; *p* ≤ 0.05 (*) was considered statistically significant). (D) Outcome of experiments and statistical analysis as in panel B but using α-sensor (**15**). (E) Selectivity of α-sensor (**15**) in human saliva with and without viral neuraminidase (mean ± SD, *n* = 5; Dixon outlier test followed by *t*-test; *p* ≤ 0.05 (*) was considered statistically significant). (F) Different concentrations of H1N1 (A/California/7/2009) incubated with α-sensor (**15**) for 10–30 min, resulting in the release of thymol (mean ± SD, *n* = 3; Dixon outlier test, followed by Kruskal–Wallis test and Dunn posthoc test; *p* ≤ 0.05 (*) was considered statistically significant).

We then studied the selectivity and sensitivity of the unmethylated sensor (**6**), to viral and bacterial neuraminidase (Figures 3C, S6–S7A). When sensor (**6**) was incubated with viral or bacterial neuraminidase, thymol concentration increased for both enzymes over 1.5 h, whereas no increase in thymol concentration could be detected for the β-unmethylated reference sensor (**6**) with viral neuraminidase (Figures 3C, S6–S7A). These results highlight the preference of neuraminidase for cleaving α-glycosidically linked substrates.^62,63^ Thus, only sensors to which a taste molecule can be coupled α-glycosidically are suitable for influenza detection. Additionally, the unmethylated sensor (**6**) exhibits no selectivity toward viral or bacterial neuraminidase and is therefore unsuitable for influenza detection.

Based on these insights, sensor (**15**) was developed (Figures 3D, S7B–S8). α-Sensor (**15**) was stable with bacterial neuraminidase but responded to viral neuraminidase within 30 min (Figures 3D, S7B–S8). As demonstrated above for the unmethylated reference sensor (**6**), the conformation of the coupled flavor moiety plays a pivotal role in its release. Thus, the flavor could only be detected in the α-glycosidically linked sensor (**15**) when exposed to viral neuraminidase. Therefore, we demonstrated that modification of *N*-acetylneuraminic acid is crucial for differentiating between viral and bacterial neuraminidase. In particular, substitution at O4 is essential for inhibiting bacterial neuraminidase, whereas substitution at O7 is required to reduce the activity of other viral neuraminidases (mumps, parainfluenza).^31–34,36^ In contrast to the sensors in test systems that require an additional trigger solution for calorimetric or chemiluminescent readout, the sensors developed here present the taste molecule required for influenza detection directly after enzymatic cleavage by viral neuraminidase (Figure S19).^35,36^

α-Sensor (**15**) and α-sensor (**6**) were stable in PBS and *M. viridifaciens* buffer for at least 1.5 h, and all our experiments were conducted within this time frame (Figures S9–S10).

The cleavage rate of the α-sensor (**15**) was concentration-dependent, with, for example, a 710-fold increase in rate at 40.0 mM compared to 0.25 mM sensor concentrations (Figure S11). We also performed an inhibition assay with the viral neuraminidase inhibitor oseltamivir phosphate. Oseltamivir in PBS at concentrations of at least 0.04 mM inhibited 1.0 mM α-sensor (**15**) cleavage, indicating competitive binding of oseltamivir and the α-sensor (**15**) at the enzyme’s active site (Figure S12).

We then expanded from buffer media to tests in human saliva (Figures 3E, S13–S17). Incubation of the α-unmethylated reference sensor (**6**) in saliva resulted in the release of thymol within 30 min.^64,65^ In contrast, no release was observed for α-sensor (**15**) in human saliva or in saliva medium spiked with bacterial neuraminidase. However, when α-sensor (**15**) was incubated in human saliva spiked with viral neuraminidase, thymol was detected within 30 min. This finding confirms previous results that α-unmethylated sensor (**6**) is nonspecifically cleaved by various neuraminidases and that modified α-sensor (**15**) is only cleaved by influenza-specific neuraminidase.

Similar to the responses seen for 4-MUNANA (Figure 3B), the α-sensor (**15**) was cleaved by living viruses (Figures 3F, S18B), resulting in a release of 0.013 to 0.028 mM thymol with 10^4^ ffu/mL, and from 0.11 to 0.27 mM thymol with 10^5^ ffu/mL. These conditions reflected clinically relevant viral neuraminidase concentrations (*vide supra*; Figure 3A). Future clinical trials should confirm our evidence with patient-reported outcomes for taste sensations, differentiating performances in our sensor in pre- and postsymptomatic stages. However, the data presented here, along with previous reports, arguably provide evidence that this sensor may successfully cover all stages. Viral titers of H1N1 are reported to decline at least 1 log_10_ unit per day after the initial spike at day 2 postinfection.^57,58^ This suggests that the cleavage conditions for the α-sensor (**15**) would be advantageous in early stage compared to late-stage patients. Using saliva from the late stage, as done here, represented a conservative assessment scenario for the sensors (Table S2).

We also calculated the amount of α-sensor (**15**) required for oral use: With thymol being detected by taste at 1100–1700 ppb^66,67^ and neuraminidase activities of 4.1 ± 1.0 mU/mL (as observed in the late-stage patient saliva; Figure 3A), 2.1–11.5 mg of sensor are predicted per use (details of the calculation are provided in the Supporting Information, section ‘Calculation of Sensor Quantity’). Future sensor designs could further reduce the amount of required sensors or the time it takes to perceive a taste sensation. For example, replacing thymol with denatonium would introduce the most bitter substance used as an accepted food supplement, with taste limits 10–100 times below those of thymol.^29,68–70^ A switch from spicy thymol to bitter denatonium, or to a sensor releasing a dye upon cleavage for visual detection, could also help circumvent potential problems arising from a loss of taste during the disease.

### Dissecting the Structural Basis for Sensor Selectivity

The selectivity profile shown by α-sensor (**15**) was further studied using crystallographic data (Figure 4A). Whereas in the *M. viridifaciens* X-ray structure (PDB: 1EUS^71^), the O4 hydroxyl group of substrates interacts with D131 (similar as found within other bacterial and mammalian neuraminidases, Figure S20), a larger pocket within the viral H1N1 analog (PDB: 3TI6 ^72^) can be addressed via substitution.^73,74^ For both cases, the carboxyl group is complexed by an arginine triad, thereby enabling interactions with the key catalytic tyrosine (Y370/406) to form oxocarbenium intermediates.^75^ Point mutations of this residue can drastically impact enzyme activity.^76,77^ Neuraminic acid forms similar interactions to the inhibitors found within these crystal structures via the distortion of its pyranose ring (Figure S21). This binding mode only allows for α-glycosidically substrates to bind, with neighboring monomers (or, in our case, thymol) that are not involved in direct interactions facing outward.^71^ We performed molecular docking of (un)­methylated sensors linked with thymol or menthol, which reflected the taste molecules used for the synthesis described above. Methylated α-sensor (**15**) and α-unmethylated sensor (**6**) fit into the active site of the viral neuraminidase, with only (**6**)—and not (**15**)—fitting into the active site of the bacterial neuraminidase. Correspondingly, affinity scores were unfavorable for methylated sensors within bacterial neuraminidase (Figures 4B, S22). This finding corroborates the measured selectivity and the possibility of exchanging thymol with other flavor elements, such as menthol, denatonium benzoate, or dye (*vide supra*).

**Figure 4:**
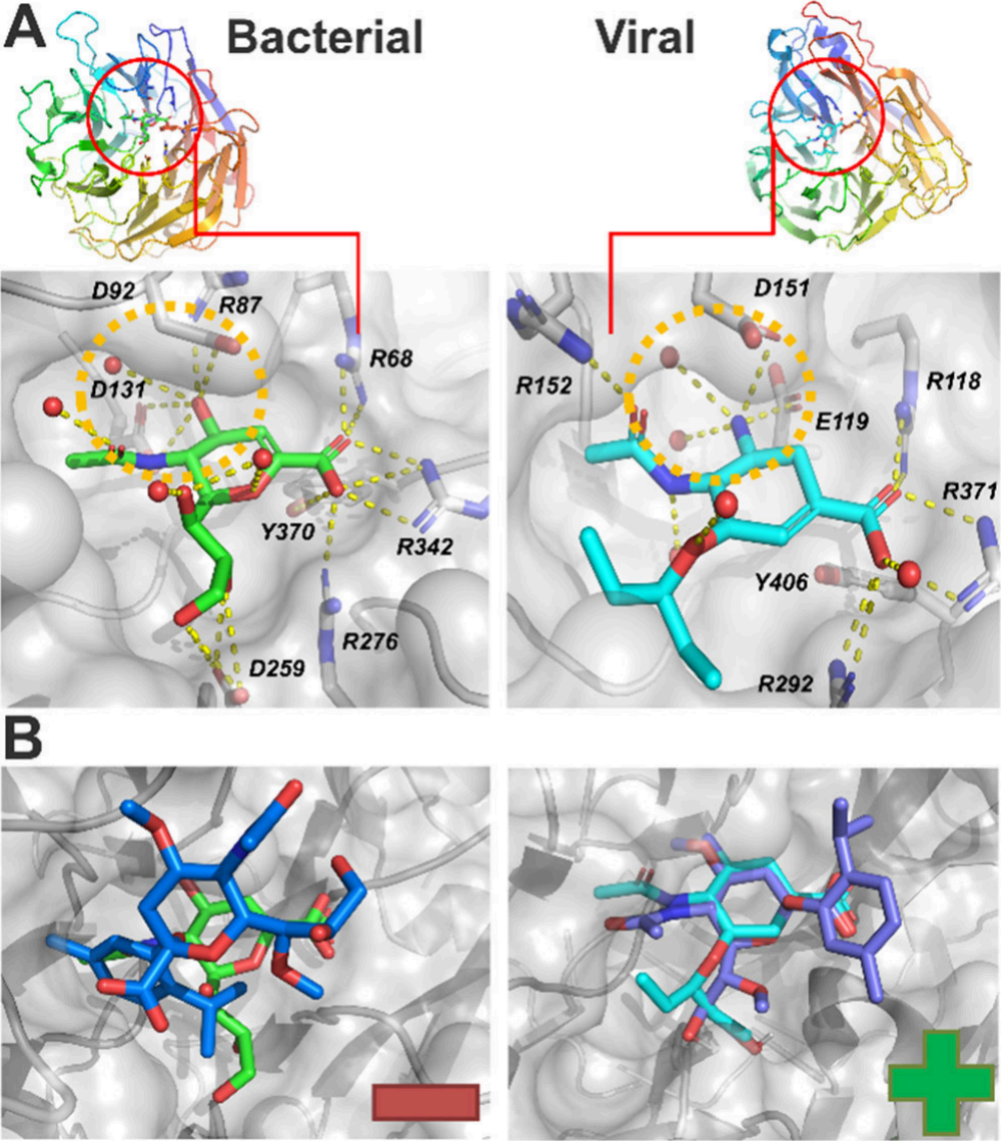
Binding modes within bacterial and viral neuraminidase. (A) Crystallized ligands oseltamivir (green) and 2-deoxy-2,3-dehydro-*N*-acetyl neuraminic acid (cyan) within PDB structures 1EUS and 3TI6, respectively. An orange circle indicates interactions of the O4 hydroxyl group with D131 for bacterial neuraminidase that is not present within the viral analog. (B) Top docking poses for α-sensor (**15**) showing binding of the pyranose ring inside the active site only for the case of viral neuraminidase (crystallized ligands are shown for reference).

## Conclusions

We demonstrated that neuraminidase cleaves α-glycosidic bonds of *N*-acetylneuraminic acid and that the selectivity toward different neuraminidases can be controlled by substituting the sugar’s hydroxyl groups (O4, O7). We additionally revealed that neuraminidase activity in infected saliva is sufficiently high to cleave our sensor designs, which feature our tongues as a 24/7 detector of influenza infection. Based on the chemical strategy outlined here, perhaps in modified form to accommodate shorter detection times, we demonstrated the potential for anyone to be able to conduct a test for influenza anywhere and at any time. Therefore, compared to existing diagnostics (Table S3), our sensor strategy addresses the need for a first-line and low-cost rapid screening tool. Future clinical studies will have to validate the value of this design based on patient-reported outcomes and the ability to stratify patients who are at risk of having influenza from healthy individuals.

## Supplementary Material



## Notes

Materials and methods, including information on chemicals, synthesis procedures, analytical results (MS, HPLC, NMR), collection of saliva samples, cell cytotoxicity, stability measurements, inhibitor measurements, enzyme kinetics, H1N1 virus cleavage experiments, neuraminidase activity determination, calculation of sensor quantity, the neuraminidase assay kit, cleavage experiments, stability measurements, calculation of sensor quantity, and docking (PDF)

## References

[1] Uyeki T. M., Hui D. S., Zambon M., Wentworth D. E., Monto A. S. (2022). Influenza. Lancet.

[2] Lozano R., Naghavi M., Foreman K., Lim S., Shibuya K., Aboyans V., Abraham J., Adair T., Aggarwal R., Ahn S. Y. (2012). Global and regional mortality from 235 causes of death
for 20 age groups in 1990 and 2010: a systematic analysis for the
Global Burden of Disease Study 2010. Lancet
(London, England).

[3] O Murchu E., Comber L., Jordan K., Hawkshaw S., Marshall L., O’Neill M., Ryan M., Teljeur C., Carnahan A., Pérez J. J. (2022). Systematic
review of the efficacy, effectiveness and
safety of recombinant haemagglutinin seasonal influenza vaccines for
the prevention of laboratory-confirmed influenza in individuals≥
18 years of age. Reviews in Medical Virology.

[4] Wei C.-J., Crank M. C., Shiver J., Graham B. S., Mascola J. R., Nabel G. J. (2020). Next-generation
influenza vaccines: opportunities and
challenges. Nat. Rev. Drug Discovery.

[5] Gilchuk I. M., Bangaru S., Gilchuk P., Irving R. P., Kose N., Bombardi R. G., Thornburg N. J., Creech C. B., Edwards K. M., Li S., Turner H. L., Yu W., Zhu X., Wilson I. A., Ward A. B., Crowe J. E. (2019). Influenza H7N9 virus neuraminidase-specific
human monoclonal antibodies inhibit viral egress and protect from
lethal influenza infection in mice. Cell Host
Microbe.

[6] Mostafa A., Abdelwhab E. M., Mettenleiter T. C., Pleschka S. (2018). Zoonotic potential
of influenza A viruses: a comprehensive overview. Viruses.

[7] Wang X., Jiang H., Wu P., Uyeki T. M., Feng L., Lai S., Wang L., Huo X., Xu K., Chen E. (2017). Epidemiology
of avian influenza A H7N9 virus in human beings across five epidemics
in mainland China, 2013–17: an epidemiological study of laboratory-confirmed
case series. Lancet Infectious Diseases.

[8] Adlhoch C., Pebody R. (2020). What to expect for the influenza season 2020/21 with
the ongoing COVID-19 pandemic in the World Health Organization European
Region. Eurosurveillance.

[9] Adlhoch C., Mook P., Lamb F., Ferland L., Melidou A., Amato-Gauci A. J., Pebody R. (2021). Very little influenza in the WHO
European Region during the 2020/21 season, weeks 40 2020 to 8 2021. Eurosurveillance.

[10] Taubenberger J. K., Reid A. H., Janczewski T. A., Fanning T. G. (2001). Integrating historical,
clinical and molecular genetic data in order to explain the origin
and virulence of the 1918 Spanish influenza virus. Philosophical Transactions of the Royal Society of London.
Series B: Biological Sciences.

[11] Johnson N. P., Mueller J. (2002). Updating the accounts:
global mortality of the 1918–1920″
Spanish″ influenza pandemic. Bulletin
of the History of Medicine.

[12] Cipriano P. F. (2018). 100 years
on: the Spanish Flu, pandemics and keeping nurses safe. International Nursing Review.

[13] Nickol M. E., Kindrachuk J. (2019). A year of terror and a century of reflection: perspectives
on the great influenza pandemic of 1918–1919. BMC Infectious Diseases.

[14] Kilbourne E. D. (2006). Influenza
Pandemics of the 20th Century. Emerg Infect
Dis..

[15] Kawaoka Y., Krauss S., Webster R. G. (1989). Avian-to-human transmission of the
PB1 gene of influenza A viruses in the 1957 and 1968 pandemics. Journal of virology.

[16] Lipatov A. S., Govorkova E. A., Webby R. J., Ozaki H., Peiris M., Guan Y., Poon L., Webster R. G. (2004). Influenza: emergence
and control. Journal of virology.

[17] Palese P. (2004). Influenza:
old and new threats. Nature medicine.

[18] U.S. Centers for Disease Control and Prevention . H5 Bird Flu: Current Situation. https://www.cdc.gov/bird-flu/situation-summary/index.html (accessed 11/12/2024).

[19] U.S. Centers for Disease Control and Prevention . National Pandemic Strategy. https://www.cdc.gov/pandemic-flu/php/national-strategy/index.html (accessed 04/08/2024).

[20] Xu R., Ekiert D. C., Krause J. C., Hai R., Crowe J. E., Wilson I. A. (2010). Structural basis of preexisting immunity
to the 2009
H1N1 pandemic influenza virus. Science.

[21] Swerdlow D. L., Finelli L., Bridges C. B. (2011). 2009 H1N1 Influenza Pandemic: Field
and Epidemiologic Investigations in the United States at the Start
of the First Pandemic of the 21st Century. Clinical
Infectious Diseases.

[22] Monto A. S., Fukuda K. (2020). Lessons From Influenza
Pandemics of the Last 100 Years. Clinical Infectious
Diseases.

[23] U.S. Centers for Disease Control and Prevention . 2009 H1N1 Pandemic (H1N1pdm09 virus). https://archive.cdc.gov/www_cdc_gov/flu/pandemic-resources/2009-h1n1-pandemic.html (acessed 11/12/2024).

[24] Council, H. S. National Strategy for Pandemic Influenza: Implementation Plan; Executive Office of the President, 2006.

[25] Morse S. S. (2007). The US
pandemic influenza implementation plan at six months. Nature medicine.

[26] Miesler T., Wimschneider C., Brem A., Meinel L. (2020). Frugal innovation for
point-of-care diagnostics controlling outbreaks and epidemics. ACS Biomaterials Science & Engineering.

[28] Ritzer J., Luhmann T., Rode C., Pein-Hackelbusch M., Immohr I., Schedler U., Thiele T., Stubinger S., Rechenberg B. v., Waser-Althaus J., Schlottig F., Merli M., Dawe H., Karpisek M., Wyrwa R., Schnabelrauch M., Meinel L. (2017). Diagnosing peri-implant
disease using
the tongue as a 24/7 detector. Nat. Commun..

[29] ter
Mors B., Driessen M. D., Seher A., Haubitz I. R., Raschig M., Nowak M., Jockel-Schneider Y., Linz C., Meinel L. (2023). The development
of matrix-metalloproteinase responsive sensors for the machine-independent
detection of oral inflammation. Sensors &
Diagnostics.

[30] Makoah N. A., Tipih T., Litabe M. M., Brink M., Sempa J. B., Goedhals D., Burt F. J. (2022). A systematic
review and meta-analysis
of the sensitivity of antibody tests for the laboratory confirmation
of COVID-19. Future virology.

[31] Beau J.-M., Schauer R. (1980). Metabolism of 4-O-methyl-N-acetylneuraminic acid a
synthetic sialic acid. Eur. J. Biochem..

[32] Liav, A. ; Hansjergen, J. A. ; Shimasaki, C. D. 4,7-Dialkoxy N-acetylneuraminic acid derivatives and methods for detection of influenza type A and B viruses in clinical specimens. CA Patent CA2237790A1, April 2, 1998.

[33] Liav A., Hansjergen J. A., Achyuthan K. E., Shimasaki C. D. (1999). Synthesis
of bromoindolyl 4,7-di-O-methyl-Neu5Ac: specificity toward influenza
A and B viruses. Carbohydrate research.

[34] Corfield A. P., Sander-Wewer M., Veh R. W., Wember M., Schauer R. (1986). The action
of sialidases on substrates containing O-acetylsialic acids. Biological Chemistry Hoppe-Seyler.

[35] Achyuthan K. E., Pence L. M., Appleman J. R., Shimasaki C. D. (2003). ZstatFlu®-II
test: a chemiluminescent neuraminidase assay for influenza viral diagnostics. Luminescence: The journal of biological and chemical luminescence.

[36] Shimasaki C., Achyuthan K., Hansjergen J., Appleman J. (2001). Rapid diagnostics:
the detection of neuraminidase activity as a technology for high-specificity
targets. Philosophical Transactions of the Royal
Society of London. Series B.

[37] Achyuthan K. E., Pence L. M., Mantell D. R., Nangeroni P. E., Mauchan D. M., Aitken W. M., Appleman J. R., Shimasaki C. D. (2003). Engineering
a chemical implementation device and an imaging device for detecting
chemiluminescence with a Polaroid high-speed detector film: application
to influenza diagnostics with the ZstatFlu®-II test. Luminescence: The journal of biological and chemical luminescence.

[38] Meisen I., Peter-Katalinić J., Müthing J. (2003). Discrimination
of Neolacto-Series Gangliosides with α2–3- and α2–6-Linked
N-Acetylneuraminic Acid by Nanoelectrospray Ionization Low-Energy
Collision-Induced Dissociation Tandem Quadrupole TOF MS. Anal. Chem..

[39] Sueki A., Matsuda K., Yamaguchi A., Uehara M., Sugano M., Uehara T., Honda T. (2016). Evaluation
of saliva as diagnostic
materials for influenza virus infection by PCR-based assays. Clin. Chim. Acta.

[40] Contreras C., Newby J. M., Hillen T. (2021). Personalized virus
load curves for
acute viral infections. Viruses.

[41] Drewnowski A. (2001). The Science
and Complexity of Bitter Taste. Nutrition Reviews.

[42] Kánya N., Kun S., Batta G., Somsák L. (2020). Glycosylation with ulosonates under
Mitsunobu conditions: scope and limitations. New J. Chem..

[43] Yang W., Liu X., Peng X., Li P., Wang T., Tai G., Li X. J., Zhou Y. (2012). Synthesis of novel N-acetylneuraminic
acid derivatives as substrates for rapid detection of influenza virus
neuraminidase. Carbohydrate research.

[44] Liav, A. ; Hardgrave, R. F. ; Blystone, S. ; Turner, G. A. ; Foundation, O. M. R. Synthesis of 4-alkoxy-n-acetylneuraminic acid. WO Patent WO1996004291A1, February 15, 1996.

[45] Šardzík R., Noble G. T., Weissenborn M. J., Martin A., Webb S. J., Flitsch S. L. (2010). Preparation of aminoethyl glycosides for glycoconjugation. Beilstein journal of organic chemistry.

[46] Norton A. K., Kok G. B., von Itzstein M. (2001). The synthesis
of C-9 modified derivatives
of the α-methyl glycoside of Kdn methyl ester. Journal of Carbohydrate Chemistry.

[47] Gao G., Schwardt O., Ernst B. (2004). Synthesis
of aryl sialosides using
Mitsunobu conditions. Carbohydrate research.

[48] Marra A., Sinay P. (1989). Acetylation of N-Acetylneuraminic Acid and Its Methyl-Ester. Carbohydrate research.

[49] Shelke S. V., Cutting B., Jiang X. H., Koliwer-Brandl H., Strasser D. S., Schwardt O., Kelm S., Ernst B. (2010). A Fragment-Based
In Situ Combinatorial Approach To Identify High-Affinity Ligands for
Unknown Binding Sites. Angew. Chem. Int. Edit.

[50] Gregar T. Q., Gervay-Hague J. (2004). Synthesis of oligomers derived from amide-linked neuraminic
acid analogues. J. Org. Chem..

[51] Carter T.
S., Mooibroek T. J., Stewart P. F. N., Crump M. P., Galan M. C., Davis A. P. (2016). Platform
Synthetic Lectins for Divalent Carbohydrate
Recognition in Water. Angew. Chem. Int. Edit.

[52] Nakamura M., Takeda K., Takayanagi H., Asai N., Ibata N., Ogura H. (1993). Studies on Sialic Acids. Part XXIX. Synthesis of Tetrazolyl Derivatives
of 3-Deoxy-D-glycero-D-galacto-2-nonulosonic Acid (KDN) as Useful
Glycosyl Donors, and Their Application for O- and C-Glycosylations. Chem. Pharm. Bull..

[53] Shidmoossavee F. S., Watson J. N., Bennet A. J. (2013). Chemical
insight into the emergence
of influenza virus strains that are resistant to Relenza. J. Am. Chem. Soc..

[54] Chefalo P., Pan Y., Nagy N., Harding C., Guo Z. (2003). Preparation and immunological
studies of protein conjugates of N-acylneuraminic acids. Glycoconjugate journal.

[55] International Organization for Standardization . ISO 10993–5:2009 Biological evaluation of mdeical devices, Part 5: Tests for in vitro cytotoxicity. 2009; p 34.

[56] Kroppenstedt R. M., Mayilraj S., Wink J. M., Kallow W., Schumann P., Secondini C., Stackebrandt E. (2005). Eight new species of the genus Micromonospora,
Micromonospora citrea sp. nov., Micromonospora echinaurantiaca sp.
nov., Micromonospora echinofusca sp. nov. Micromonospora fulviviridis
sp. nov., Micromonospora inyonensis sp. nov., Micromonospora peucetia
sp. nov., Micromonospora sagamiensis sp. nov., and Micromonospora
viridifaciens sp. nov. Syst. Appl. Microbiol.

[57] Canini L., Carrat F. (2011). Population Modeling of Influenza A/H1N1 Virus Kinetics
and Symptom Dynamics. J. Virol..

[58] Baccam P., Beauchemin C., Macken C. A., Hayden F. G., Perelson A. S. (2006). Kinetics
of Influenza A Virus Infection in Humans. J.
Virol..

[59] Moscona A. (2005). Neuraminidase
inhibitors for influenza. New England Journal
of Medicine.

[60] Marathe B. M., Lévêque V., Klumpp K., Webster R. G., Govorkova E. A. (2013). Determination of Neuraminidase Kinetic Constants Using
Whole Influenza Virus Preparations and Correction for Spectroscopic
Interference by a Fluorogenic Substrate. PLoS
One.

[61] Klenow L., Elfageih R., Gao J., Wan H., Withers S. G., de Gier J.-W., Daniels R. (2023). Influenza virus and
pneumococcal
neuraminidases enhance catalysis by similar yet distinct sialic acid-binding
strategies. J. Biol. Chem..

[62] Juge N., Tailford L., Owen C. D. (2016). Sialidases
from gut bacteria: a mini-review. Biochemical
Society transactions.

[63] Yuan L., Zhao Y., Sun X.-L. (2020). Sialidase substrates for Sialdiase
assays-activity, specificity, quantification and inhibition. Glycoconjugate Journal.

[64] Nijjar M. S., Pritchard E. T., Dawes C., Philips S. R. (1970). Neuraminidase
activity
in the salivary glands of rats and human saliva. Archives of Oral Biology.

[65] Perlitsh M. J., Glickman I. (1966). Salivary Neuraminidase.
II. Its Source in Human Whole
Saliva. Journal of Dental Research.

[66] Wilson C. W., Shaw P. E. (1981). Importance of thymol, methyl N-methylanthranilate,
and monoterpene hydrocarbons to the aroma and flavor of mandarin cold-pressed
oils. J. Agric. Food Chem..

[67] Sánchez L. M., Ramos M. J. G., del
Mar Gómez-Ramos M., Vazquez P. P., Flores J. M. (2021). Presence,
persistence and distribution of thymol in
honeybees and beehive compartments by high resolution mass spectrometry. Environmental Advances.

[68] Meinel, L. ; Luehmann, T. ; Raschig, M. Aminomethyl-functionalized denatonium derivatives, their preparation and use. US Patent US20210087138A1, March 25, 2021.

[69] Schiffman S. S., Gatlin L. A., Frey A. E., Heiman S. A., Stagner W. C., Cooper D. C. (1994). Taste perception of bitter compounds in young and elderly
persons: Relation to lipophilicity of bitter compounds. Neurobiol. Aging.

[70] Stachowiak W., Wysocki M., Niemczak M. (2022). “Bitter”
Results: Toward
Sustainable Synthesis of the Most Bitter Substances, Denatonium Saccharinate
and Denatonium Benzoate, Starting from a Popular Anesthetic, Lidocaine. J. Chem. Educ..

[71] Gaskell A., Crennell S., Taylor G. (1995). The three
domains of a bacterial
sialidase: a β-propeller, an immunoglobulin module and a galactose-binding
jelly-roll. Structure.

[72] Vavricka C. J., Li Q., Wu Y., Qi J., Wang M., Liu Y., Gao F., Liu J., Feng E., He J. (2011). Structural
and Functional Analysis of Laninamivir and its Octanoate Prodrug Reveals
Group Specific Mechanisms for Influenza NA Inhibition. PLOS Pathogens.

[73] Holzer C. T., Von Itzstein M., Jin B., Pegg M. S., Stewart W. P., Wu W.-Y. (1993). Inhibition of sialidases
from viral, bacterial and mammalian sources
by analogues of 2-deoxy-2,3-didehydro-N-acetylneuraminic acid modified
at the C-4 position. Glycoconjugate Journal.

[74] Weck S., Robinson K., Smith M., Withers S. (2015). Understanding viral
neuraminidase inhibition by substituted difluorosialic acids. Chem. Commun..

[75] Vavricka C. J., Liu Y., Kiyota H., Sriwilaijaroen N., Qi J., Tanaka K., Wu Y., Li Q., Li Y., Yan J. (2013). Influenza
neuraminidase operates via a nucleophilic mechanism and can be targeted
by covalent inhibitors. Nat. Commun..

[76] Hsu K. C., Hung H. C., HuangFu W. C., Sung T. Y., Eight
Lin T., Fang M. Y., Chen I. J., Pathak N., Hsu J. T., Yang J. M. (2017). Identification of neuraminidase inhibitors against
dual H274Y/I222R mutant strains. Sci. Rep.

[77] Watson J. N., Dookhun V., Borgford T. J., Bennet A. J. (2003). Mutagenesis of the
conserved active-site tyrosine changes a retaining sialidase into
an inverting sialidase. Biochemistry.

