# Supplementary material for: A Viral Neuraminidase-Specific Sensor for Taste-Based Detection of Influenza
Source: ACS Cent Sci. 2025 Oct 1;11(11):2172–9. doi: 10.1021/acscentsci.5c01179 (PMC12670280; doi:10.1021/acscentsci.5c01179)
Supplement: Supplementary file 1 [file oc5c01179_si_001.pdf]

A Viral Neuraminidase-Specific Sensor for Taste-Based Detection of Influenza

Supporting Information

Martina Raschig,[a] Marcus Gutmann,[a] Josef Kehrein,[a] Eberhard Heller,[a] Michael Bomblies,[a] Marcel Groß,[b] Oskar Steinlein,[a] Peggy Riese,[f] Stephanie Trittel,[f] Tessa Lühmann,[a] Carlos A. Guzmán,[f] Jürgen Seibel,[b] Heinrich Jehle,[c] Christian Linz,[d] Stephan Hackenberg,[e] Lorenz Meinel*[a, g]

[a] Institute of Pharmacy and Food Chemistry, University of Würzburg, Am Hubland, 97074 Würzburg, Germany

[b] Institute of Organic Chemistry, University of Würzburg, Am Hubland, 97074 Würzburg, Germany

[c] Bettackerstr. 17, 72636 Frickenhausen, Germany

[d] Faculty of Medicine and University Hospital Cologne, Department of Oral, Maxillofacial and Plastic Surgery, University of Cologne, Kerpener Str. 62, 50937 Cologne, Germany

[e] Department of Otorhinolaryngology, Plastic, Aesthetic and Reconstructive Head and Neck Surgery, University Hospital Würzburg, 97080 Würzburg, Germany

[f] Department Vaccinology and Applied Microbiology (VAC), Helmholtz Centre for Infection Research (HZI), 38124 Braunschweig, Germany

[g] Helmholtz Institute for RNA-Based Infection Research (HIRI), Helmholtz Center for Infection Research (HZI), 97080 Würzburg, Germany

Supporting Text – Materials and Methods

Table S1 – S3

Supporting Figures S1-S44

Supporting References

Supporting Text

Materials and Methods

Ethical Declaration and Safety Statement

Experiments involving human saliva were approved by the local ethics committee (approval number 133/17-sc, "Medizinische Ethikkommission" of the University of Würzburg). We hereby confirm that informed consent of all participating subjects or their next of kin was obtained. Furthermore, no unexpected or unusually high safety hazards were encountered.

Chemicals

N-Acetylneuraminic acid was purchased from Biosynth (Bratislava, Slovakia). Amberlite® IRC 120H hydrogen form, camphor sulfonic acid, barium hydroxide octahydrate, sodium hydroxide (NaOH), acetic anhydride (Ac_2O), acetyl chloride (AcCl), thymol, diethyl azodicarboxylate (DEAD) (40 % solution in THF), triphenylphosphine (polymer-supported), lithium hydroxide monohydrate, sodium methoxide (5.4 M solution in methanol), formic acid (FA), trifluoro acetic acid (TFA), hydrochloric acid (HCl, 37%), phosphate buffered saline (PBS), Dulbecco's modified Eagle's medium (DMEM) and penicillin-streptomycin (P/S) were purchased from Sigma Aldrich. Fetal bovine serum (FCS/FBS) was from GIBCO life technologies (Carlsbad, CA). WST-1 was purchased from Roche (Basel, Switzerland). Recombinant Influenza A Virus H1N1 neuraminidase and recombinant *M. viridifaciens* neuraminidase were from (R&D Systems, Minneapolis, MN, US). Barium oxide and iodomethane were purchased from Acros Organic (Thermo Fisher Scientific company, Waltham, MA, US). Triethylamine was purchased from Grüssing GmbH (Filsum, Germany). Anhydrous solvents (methanol, acetonitrile, N,N-dimethylformamide, dichloromethane and pyridine) were purchased from Sigma Aldrich and anhydrous acetone from VWR chemicals (Radnor, PA, US). Deuterated solvent (CD_3OD-d_4) was purchased from Eurisotop, Deutero GmbH and Sigma Aldrich (Schnelldorf, Germany). All other chemicals used were at least chemical grade and were purchased from Sigma Aldrich (unless noted otherwise).

Column Chromatography

For purification by column chromatography, silica gel 60 – 200 µm (VWR chemicals) was used as stationary phase.

For reversed phase column chromatography (Medium-Pressure Liquid Chromatography, MPLC) an Interchim Puri Flash 430 instrument (Interchim Deutschland GmbH, Mannheim, Germany) was used,

with CHROMABOND Flash columns (C18 silica gel, Macherey-Nagel GmbH & Co. KG). The solvent mixtures are indicated with the respective syntheses.

Microwave systems

Reactions under microwave irradiation were performed in an MLS-Ethos-CFR 1600 system (MLS-GmbH, Leutkirch Germany; method 1: heat to 66 °C for 4 min (max. 300 W), hold at 66 °C for 10 h (max. 300 W), cool down; method 2: heat to 69 °C for 4 min (max. 300 W), hold at 69 °C for 5 h (max. 300 W), cool down) and an MLS-Synthwave system (MLS-GmbH, Leutkirch, Germany); method 3 heat to 100 °C for 3 min, hold at 100 °C for 5 h, cool down, 13.6 bar argon).

Thin-Layer Chromatography (TLC)

Analytical thin-layer chromatography (TLC, precoated silica gel 60 GF254 plates, Macherey Nagel GmbH & Co. KG, Düren, Germany) was used for monitoring reaction progress and product identification after column chromatography. For substance detection the TLC plates were stained (0.6 g *N*-(1-naphthyl)ethylenediamine, 200 mL methanol, 10 mL sulfuric acid) and then heated.

Freeze Drying

Samples previously frozen at -80 °C or in liquid nitrogen were freeze-dried using a system from LMS Consult GmbH & Co. KG (Brigachtal, Germany).

NMR Spectroscopy

NMR spectra were recorded with a Avance III 400 MHz spectrometer (Bruker, Karlsruhe, Germany). Spectra were evaluated with TopSpin 3.6.1 and calibrated to the residual solvent signal (CD_3OD-d_4 3.31/49.0). Coupling constants J are given in Hz and the chemical shifts in ppm. Multiplicities were defined as m (multiplet), s (singlet), d (doublet), t (triplet) and dd (doublet of doublet).

Liquid Chromatography-Mass Spectrometry (LC-MS)

Mass spectra were recorded with a Single Quadrupole system, consisting of an LC20AB liquid chromatograph, an SPD-20A UV/Vis detector and an LC−MS 2020 (Shimadzu Scientific Instruments, Columbia, MD, USA). First, the products were separated by LC using a Synergi 4 µm fusion-RP column (4.6 × 150 mm) (Phenomenex Inc., Torrance, CA) with eluent A 0.1% (v/v) formic acid in water and eluent B 0.1% (v/v) formic acid in methanol. The detection wavelength was set to 214 nm or 254 nm.

Detection range of subsequent mass analysis was set to 60 to 1000 m/z. All samples were analyzed using the positive mode.

High-resolution mass spectrometry (HRMS)

HRMS spectra were recorded with a Bruker Daltonics micrOTOF (Bruker Corporation, Billerica, MA, USA) with electron spray ionization (ESI). Depending on the substance analyzed, measurements were taken in either positive or negative mode.

High Performance Liquid Chromatography (HPLC)

HPLC measurements were performed on an Agilent 1260 infinity II HPLC (Agilent Technologies Inc., Waldbronn, Germany) equipped with a vial sampler (G7129C, Agilent), a pump (G7104C, Agilent), a Quick-Connect heat exchanger (G7116-60051) and a VWD detector (G7114A). As stationary phase a Zorbax-Eclipse XDB-C18 (Agilent) was used. As mobile phase A 0.1 % FA in Millipore water was used and as B 0.1 % FA in acetonitrile. Flow was set to 1 mL/min and detection wavelength to λ = 280 nm. The mobile phase A was held at 80 % for 2 min, then decreased to 5% in 2 min, followed by a hold of 2 min at 5%, and increased back to 80% in 2 min, and held at 80% for 2 min (method A).

For experiments in which the neuraminidase cleavage experiments and buffer stability measurements were directly performed in the HPLC vial sampler, the sampler's temperature was set to 37 °C. In all other experiments, the temperature was set between 4 and 10 °C.

Cell cytotoxicity

The cytotoxicity of the α- and ß-anomers of sensor (**15**) was investigated using a formazan assay. Stock solutions were dissolved in growth medium (DMEM + 10% FCS + 1% P/S) to a final concentration of 2 mM. HEK 293 (ATCC CRL-1573) and NIH 3T3 (ATTC CRL-1658) were maintained in T75 cell culture flasks in growth medium at 37 °C under 5% CO_2. For cytotoxicity assessment, cells were seeded in a 96-well plate (4 x 10^3 cells/mL, 100 µL per well). Dilution series of the α- and ß-anomers of sensor (**15**) were conducted at concentrations from 2 mM to 7.8 nM in growth medium and added to the cells (1:1). Cells were incubated for 24 h at 37 °C and 5% CO_2, respectively. After incubation, 10 µL WST-1 reagent was added to each well and cells were incubated for up to four hours at 37 °C according to the manufacturer instructions. Every 60 min, cells were analyzed, and the absorbance of the soluble formazan product at 450 nm, as well as background noise at 630 nm, was determined using an Infinite M Plex microplate reader (Tecan Group Ltd., Männedorf, Switzerland). We determined the relative cell viability (proliferation) with respect to the untreated sample.

Stability measurements

The α-sensor (**15**) samples were weighed into screw-cap jars and transferred to flanged glass vials under an argon atmosphere. The sensor was stored under defined conditions (temperature and humidity controlled (Sensor - Libero CE, Elpro, Schorndorf, Germany)): –20 °C, 4 °C, 25 °C at 60% relative humidity (rh), and 50 °C at 75% rh. Samples at -20 °C were stored in a freezer, while storage at 4 °C took place in a temperature-controlled room. To maintain the humidity conditions at 25 °C and 50 °C, desiccators with saturated NaBr and NaCl solutions were placed in drying cabinets, respectively. After 1, 2, and 4 weeks, the content of the α-sensor (**15**) was determined using HPLC (method A, 40 µL injection volume, λ = 280 nm). Samples stored at temperatures below -65 °C served as reference. All measurements were analyzed three times per timepoint.

Neuraminidase Storage

Recombinant Influenza A virus H1N1 neuraminidase (R&D Systems) and recombinant *M. viridifaciens* neuraminidase (R&D Systems) were used without activation. Influenza A virus H1N1 Neuraminidase solution (5 µg, 22.7 µL, 0.22 mg/mL in 16 mM Tris, 200 mM NaCl and 20% (v/v) Glycerol, pH 7.5) was diluted with assay buffer (27.3 µL, 50mM Tris, 5 mM $CaCl_2$, 200 mM NaCl, pH 7.5) resulting in a concentration of 0.1 µg/µL. 5 µL aliquots were stored at -20 °C prior use. *M. viridifaciens* Neuraminidase solution (10 µg, 22.7 µL, 0.44 mg/mL in 25 mM Tris and 150 mM NaCl, pH 7.5) was diluted with assay buffer (77.2 µL, 50 mM sodium acetate, 150 mM NaCl, pH 4.5, herein referred to as *M. viridifaciens* buffer) resulting in a concentration of 0.1 µg/µL. 5 µL aliquots were stored at -20 °C prior use.

Collection of Saliva Samples

In the first cohort (2017/2018), saliva samples were obtained from 35 patients by oral swaps. Patients contributed to our study only after hospitalization and positive PCR testing, thereby resembling cases of late-stage diseases (~4-7 days post-infection). 17 samples were excluded due to insufficient saliva extraction from oral swabs, while two samples were omitted due to suspected blood contamination, indicated by a red color. Due to poor saliva collection from the oral swaps, saliva was collected directly into sample tubes (spitting method) in cohort 2 (2022/2023), so that all but one of the 19 samples could be analyzed by Neuraminidase Assay Kit Fluorometric Blue. Infections were detected via polymerase chain reaction (FTD TM Respiratory pathogens 21 panel, Fast track Diagnostics, Siemens Healthineers, Erlangen, Germany).

Neuraminidase Assay Kit Fluorometric Blue

The neuraminidase assay was performed as described in the assay protocol.[1] In brief: The ability of the purchased neuraminidases to cleave glycosidic bonds was analyzed in a concentration range from 0.5

ng/µL (2.5 µL neuraminidase stock solution to 497.5 µL assay buffer) to 0.008 ng/µL. The saliva samples were analyzed undiluted after centrifugation. The reaction solutions were incubated for 1 h at 37 °C and the reaction products were subsequently analyzed at 365 nm absorbance and 450 nm emission. The enzyme activity of the purchased neuraminidases or the neuraminidase activity in saliva is given in mU/mL.

Stability Measurements of α-Sensor (15) and α-Sensor (6) in Buffers

The sensors were dissolved in the respective buffers (PBS buffer and *M. viridifaciens* assay buffer) at a final concentration of 0.13 mM, incubated at 37 °C and analyzed on the HPLC (method A, 20 µL injection volume, λ = 280 nm) every 30 min over a period of 90 min. Stability measurements were conducted as technical triplicate.

Neuraminidase Cleavage Experiments with α-/ß-Sensor (15), α-/ß-Sensor (6)

Stock solutions of H1N1 neuraminidase (viral neuraminidase) and *M. viridifaciens* neuraminidase (bacterial neuraminidase) were prepared for neuraminidase cleavage experiments. Each neuraminidase was thawed and 2.5 µL viral neuraminidase was diluted in 497.5 µL PBS (0.5 ng/µL, 10 mU/mL), while 2.5 µL bacterial neuraminidase was initially diluted in 497.5 µL of *M. viridifaciens* buffer. This solution was then successively diluted 1:1 five times (0.016 ng/µL, 10 mU/mL) with *M. viridifaciens* buffer. The respective sensors were weighed and diluted in PBS to achieve a concentration of 0.25 mM for α-sensor (**15**) and β-sensor (**15**) and 0.27 mM for α-sensor (**6**) and β-sensor (**6**). The sensor solutions were mixed in a 1:1 ratio with the prepared neuraminidase solutions, yielding in a final sensor concentration of 0.13 mM and a final neuraminidase concentration of 0.25 ng/mL (5 mU/mL) for viral neuraminidase and 0.008 ng/mL (5 mU/mL) for bacterial neuraminidase. The samples were incubated at 37 °C and analyzed every 30 min over a period of 90 min by HPLC (method A, 20 µL injection volume, λ = 280 nm). The samples analyzed had a sensor concentration of 0.13 mM and neuraminidase concentrations of 0.25 ng/µL (5 mU/mL) for viral neuraminidase and 0.008 ng/µL (5 mU/mL) for bacterial neuraminidase. All experiments with sensor (**15**) were performed as technical triplicate. The experiments with α-sensor (**6**) were performed as technical triplicate. In contrast, the experiments involving β-sensor (**6**) with viral neuraminidase, were performed in duplicate.

Stability Measurements of α-Sensor (15) and α-Sensor (6) in Saliva

The sensors were dissolved in PBS buffer at a concentration of 0.53 mM. The resulting solutions were mixed 1:1 with saliva, yielding in a final sensor concentration of 0.27 mM and incubated at 37°C. Samples were collected at time points 0, 30, 60, and 90 min, subsequently mixed 1:1 with methanol, and centrifuged for 10 min at $13.4*10^3$ rpm. The supernatant (sensor concentration 0.13 mM) was

analyzed using HPLC (method A, 20 µL injection volume, λ = 280 nm). To prevent further degradation of the sensor in the saliva, the samples were stored at 4°C until analysis. The experiments were performed in biological triplicate (n=3) for sensor (**6**) and with five biological replicates (n=5) for sensor (**15**).

Neuraminidase Cleavage Experiments with α-Sensor (15) in Saliva

H1N1 neuraminidase (viral neuraminidase) and *M. viridifaciens* neuraminidase (bacterial neuraminidase) were thawed and 2.5 µL viral neuraminidase was diluted in 497.5 µL saliva (0.5 ng/µL, 10 mU/mL), while 2.5 µL bacterial neuraminidase was initially diluted in 497.5 µL of *M. viridifaciens* buffer. This solution was then serially diluted 1:1 four times *M. viridifaciens* buffer and one time 1:1 with saliva (0.016 ng/µL, 10 mU/mL).
α-sensor (**15**) was diluted in PBS at a concentration of 0.53 mM. The sensor solution was mixed in a 1:1 ratio with the prepared neuraminidase-saliva solutions, yielding in a final sensor concentration of 0.27 mM, and incubated at 37 °C. Samples were collected every 30 min over a period of 90 min, mixed 1:1 with methanol and centrifuged for 15 min at 13.4 * 10^3 rpm. The supernatant (sensor concentration 0.13 mM) was analyzed using HPLC (method A, 20 µL injection volume, λ = 280 nm). To prevent further degradation of the sensor in the saliva, the samples were stored at 4°C until analysis. The experiments were performed with five biological replicates (n=5).

Inhibitor measurements

For neuraminidase inhibition experiments, oseltamivir phosphate was prepared at concentrations of 2 mM and 0.08 mM in PBS, including 20 mU/mL viral neuraminidase, respectively. As a control, 20 mU/mL viral neuraminidase in PBS without inhibitor was used. α-sensor (**15**) solutions (2 mM in PBS) were then mixed 1:1 with the prepared inhibitor or control solutions, yielding final concentrations of 1 mM sensor, 10 mU/mL neuraminidase, and 0.04 mM or 1.0 mM oseltamivir phosphate, respectively. Samples were incubated at 37 °C and taken after 10 min, 20 min, and 30 min. Each sample was diluted 1:1 with methanol and analyzed by HPLC (sensor concentration within the sample solution 0.5 mM; method A, 40 µL injection volume, λ = 280 nm). The measurements were performed as three technical replicates (n=3).

Enzyme kinetics

A stock solution of viral neuraminidase (20 mU/mL) was prepared in PBS and mixed in a 1:1 ratio with a serial dilution of α-sensor (**15**) ranging from 80 mM to 0.5 mM, resulting in 40 to 0.25 mM concentrations and a viral neuraminidase activity of 10 mU/mL. The samples were incubated at 37°C, taken every 5 min over a period of 30 min (except for 20 mM and 40 mM, samples were taken every 10 min over a period of 30 min) and mixed 1:1 with methanol. The samples were analyzed by HPLC

(method A, 40 µL injection volume, λ = 280 nm). The measurements were performed as technical triplicates (n=3).

H1N1 virus cleavage experiments with α-sensor (15)

A solution containing approximately 2.0×10^6 H1N1 (A/California/7/2009) foci forming units/mL (ffu/mL) was serially diluted to obtain a range of 2.0×10^6 to 2.0×10^2 ffu/mL. Each dilution was mixed 1:1 with α-sensor (**15**) (8.0 mM in PBS), resulting in final concentration of 1.0×10^6 to 1.0×10^2 ffu/mL and a final α-sensor (**15**) concentration of 4.0 mM. Samples were incubated at 37 °C with 5% CO_2. Every 10 min over a 30-min period, aliquots were taken, diluted 1:1 with methanol, centrifuged for 10 min at 10,000 rpm, and analyzed using HPLC (sensor concentration 2.0 mM, method A, 40°µL injection volume, λ = 280 nm). The experiments were performed in three technical replicates (n=3).

H1N1 virus cleavage experiments with 4-MUNANA

A solution containing 2.0×10^6 H1N1 (A/California/7/2009) ffu/mL was serially diluted to obtain a range of 2.0×10^6 to 2.0×10^2 ffu/mL. Each dilution was mixed 1:1 with 4-methylumbelliferyl-N-acetyl-α-D-neuraminic acid sodium salt hydrate (4-MUNANA, 8.0 mM in PBS), resulting in final concentration of 1.0×10^6 to 1.0×10^2 ffu/mL and a final 4-MUNANA concentration of 4.0 mM. Samples were incubated at 37 °C with 5% CO_2. Every 10 min over a 30-min period, aliquots were taken, diluted 1:1 with methanol, centrifuged for 10 min at 10,000 rpm, and analyzed. Fluorescence of the 4-methylumbelliferone (4MU) product was measured with excitation at λ_{Ex} = 365 nm (±9 nm) and emission at λ_{Em} = 445 nm (±20 nm) using an Infinite M Plex microplate reader (Tecan Group Ltd., Männedorf, Switzerland). All experiments were performed as technical triplicates (n=3).

Determining viral neuraminidase activity of the H1N1 (A/California/7/2009)

To assess viral neuraminidase activity of H1N1 (A/California/7/2009), a standard curve was generated using 4MU and correlated with cleaved 4-MUNANA samples of H1N1 (A/California/7/2009) and commercially available viral neuraminidase. Solutions containing 4.0 mM 4-MUNANA (in PBS) and viral neuraminidase at concentrations ranging from 20 mU/mL to 0.04 mU/mL were prepared and incubated at 37 °C for 10, 20, and 30 min, respectively. Subsequently, the reactions were stopped by adding an equal volume of methanol. Samples treated with either viral neuraminidase or H1N1 (A/California/7/2009, *vide supra*) were analyzed using an Infinite M Plex microplate reader (Tecan Group Ltd., Männedorf, Switzerland) with excitation at λ_{Ex} = 365 nm (±9 nm) and emission at λ_{Em} = 445 nm (±20 nm). 4MU standard curve was used to estimate neuraminidase activity in H1N1 samples by correlation to the activity observed with the commercial viral neuraminidase.

Calculation of Sensor Quantity

Considering the taste threshold for thymol, which ranges between 1100 and 1700 ppb, and a saliva volume between 2 and 7 mL, a sensor quantity of approximately 2.1 mg to 11.5 mg must be incorporated.[2-4] Of note, other literature describes an even lower aroma threshold for thymol (86 – 790 ppb).[5] Furthermore, the smell of the sensor could be detected even earlier than the taste.

The necessary sensor quantity was calculated as described in the following. Minimum concentrations of thymol based on the reported thresholds in ppb were first converted to molarity:

$$1\,ppb \;=\; 1\,\frac{\mu g}{L} \;=\; 0.001 mg/L$$

$$c_{limit} = thymol\,[mM] = \frac{c\,[\frac{mg}{L}]}{MW_{thymol}\,[\frac{g}{mol}]}$$

$c_{Limit}^{1100} \;=\; 1100\text{ ppb} \;=\; 0.00732\text{ mM}$

$c_{Limit}^{1700} \;=\; 1700\text{ ppb} \;=\; 0.01132\text{ mM}$

A sensor undergoes a 1:1 decomposition into thymol and 4,7-di-O-methyl-*N*-acetylneuraminic acid. Based on experimental data (measurements with 1 x 10^4 ffu/mL equalling 4 mU/mL), the yield of thymol from an initial sensor concentration of 4 mM (c_0) was measured at various incubation times.

$c_t^{10} \;=\; 0.0130\text{ mM}$

$c_t^{20} \;=\; 0.0213\text{ mM}$

$c_t^{30} \;=\; 0.0275\text{ mM}$

The release of thymol and the necessary minimum concentrations were then used to determine the sensor concentration needed $c_{sensor,needed}$ to surpass the threshold at each timepoint:

$$c_{sensor,\,needed}[mM] \;=\; \frac{c_0\;[mM] * \;c_{limit}[mM]}{c_t}$$

	c_{Limit}^{1100}	c_{Limit}^{1700}
**10 min**	2,262 mM	3,497 mM
**20 min**	1,373 mM	2,124 mM
**30 min**	1,064 mM	1,646 mM

The corresponding sensor masses (mg) for 2 mL and 7 mL (V_{saliva}) representing theoretical saliva volumes used as test environments were then calculated:

$$m_{sensor}[mg] = C_{sensor}\;[mM] * \;V_{saliva}[L] * MW_{sensor}[g/mol]$$

C_{Limit}^{1100}		C_{Limit}^{1700}	
2 mL	7 mL	2 mL	7 mL

**10 min**	2,122 mg	7,428 mg	3,282 mg	11,487 mg
**20 min**	1,289 mg	4,510 mg	1,993 mg	6,975 mg
**30 min**	0,999 mg	3,496 mg	1,545 mg	5,407 mg

Molecular docking

Molecular docking was performed by first preparing the bacterial and viral proteins (PDBs: 1EUS and 3TI6) using DockPrep within ChimeraX.[6] The protonated receptors were further processed via pdb4amber from AmberTools and converted to pdbqt files via meeko.[7] Except for the crystallized ligands used for redocking, all compounds of interest were initially built and energy minimized within Avogadro and converted via meeko, starting from a sialic acid structure with a distorted pyranose ring conformation, as found within relevant crystal structures (PDB: 8DWB).[8] We investigated methylated and unmethylated sensors linked with thymol and menthol, as well as methylated and unmethylated sialic acid for reference. Docking was performed with GNINA 1.3, a new variant of AutoDock Vina that utilizes an ensemble of convolutional neural networks (CNN) for re-scoring generated poses. A cubic grid box with side lengths of 20 Å was defined, with its center placed at the center of geometry of the original ligand.[9] An exhaustiveness value of 16 was applied with a minimum RMSD filter of 0.5 Å and 20 rotations of poses evaluated (parameter cnn_rotation). For all docking runs, the top 10 poses were output and investigated. Docking validation was performed by redocking of native ligands, leading to top poses with RMSD values < 2 Å (0.924 Å for the bacterial and 0.546 Å for the viral variant, **Figure S22B**).

Synthetic Procedures

During the synthesis of the precursors, the α and β anomers of the compound were not separated. The NMR spectra given, represents the compound shown in the figures. All reactions with anhydrous solvents were carried out in an argon atmosphere.

*Synthesis of Unmethylated Reference Sensor (**6**)*

Compound (**2**)[10]

To a suspension of *N*-acetylneuraminic acid (10.0 g, 32.3 mmol) in methanol (300 mL) TFA (1.0 mL, 13.1 mmol) was added. The mixture was stirred at room temperature until a clear solution was formed

(3 – 4 days). The solvent was evaporated to dryness to give compound (**2**) as colorless solid (10.4 g, 32.1 mmol, 99%).

^{1}H NMR (400 MHz, CD_3OD) δ 1.89 (dd, 1H, *J* = 12.8, 11.4 Hz), 2.02 (s, 3H), 2.22 (dd, 1H, *J* = 12.9, 4.9 Hz), 3.45 – 3.52 (m, 1H), 3.58 – 3.66 (m, 1H), 3.67 – 3.74 (m, 1H), 3.78 (s, 3H), 3.79 – 3.87 (m, 2H), 3.97 – 4.09 (m, 2H).

^{13}C NMR (100 MHz, CD_3OD) δ 22.6, 40.7, 53.1, 54.3, 64.9, 67.9, 70.2, 71.7, 72.1, 96.7, 171.8, 175.1.

Compound (**3**)[11, 12]

Compound (**2**) (10.1 g, 31.2 mmol) was treated with Ac_2O (73 mL, 0.77 mol) and anhydrous pyridine (69 mL, 0.86 mol) and stirred overnight at room temperature. The organic layer was extracted with saturated sodium bicarbonate ($NaHCO_3$) solution (150 mL) and brine (150 mL). The aqueous phase was back extracted with ethyl acetate and the combined organic layers were dried over sodium sulphate (Na_2SO_4). After removing the solvent under reduced pressure, the crude product was purified by column chromatography (silica gel, 50 – 100% ethyl acetate in cyclohexane) to give compound (**3**) as colorless solid (14.9 g, 27.9 mmol, 89%).

^{1}H NMR (400 MHz, CD_3OD) δ 1.86 (s, 3H), 1.97 – 2.06 (m, 10H), 2.11 (s, 3H), 2.15 (s, 3H), 2.53 (dd, 1H, *J* = 13.4, 5.0 Hz), 3.76 (s, 3H), 4.00 – 4.13 (m, 2H), 4.14 – 4.22 (m, 1H), 4.42 – 4.50 (m, 1H), 5.08 (td, 1H, *J* = 6.2, 2.6 Hz), 5.14 – 5.24 (m, 1H), 5.37 – 5.42 (m, 1H).

^{13}C NMR (100 MHz, CD_3OD) δ 20.6, 20.7, 20.8, 20.9, 22.7, 37.1, 49.9, 53.6, 63.1, 68.8, 70.0, 71.8, 73.4, 98.7, 168.1, 170.0, 171.5, 171.5, 171.9, 172.4, 173.4.

HRMS (ESI+) *m/z*: calculation for $[C_{22}H_{31}NNaO_{14}]^{+}$ 556.16368; found 556.16637.

Compound (**4**)[13]

Compound (**3**) (12.0 g, 22.5 mmol) was dissolved in anhydrous dichloromethane (75.0 mL) and cooled to -5 °C. AcCl (27.4 mL, 0.39mol) and HCl (3.4 mL, 40.7 mmol) were added, and the reaction mixture was stirred for 30 min at -5 °C. After stirring overnight at room temperature, the reaction mixture was extracted with water (150 mL), saturated $NaHCO_3$ solution (150 mL) and brine (150 mL). The organic solvent was evaporated under reduced pressure to yield compound (**4-1**) as a colorless solid (10.8 g, 21.2 mmol, 94%).

HRMS (ESI-) *m/z*: calculation for $[C_{20}H_{27}ClNO_{12}]^{-}$ 508.1227; found 508.1224 and calculation for $[C_{20}H_{28}Cl_2NO_{12}]^{-}$ 544.0994; found 544.0995.

Compound (**4-1**) (4.0 g, 7.8 mmol) was dissolved in water:acetonitrile (80 mL, 1:1) and stirred for 3 hours at room temperature. The solvent was evaporated to yield compound (**4**) as colorless solid (3.8 g, 7.7 mmol, 98%).

^{1}H NMR (400 MHz, CD_3OD) δ 1.84 (s, 3H), 1.96 – 1.99 (m, 4H), 2.00 (s, 3H), 2.04 (s, 3H), 2.09 (s, 3H), 2.26 (dd, 1H, *J* = 5.0, 12.7 Hz), 3.78 – 3.80 (m, 3H), 3.94 – 4.02 (m, 1H) 4.08 (dd, 1H, *J* = 7.2, 12.3 Hz), 4.31 (dd, 1H, *J* = 2.3, 10.6 Hz), 4.56 (dd, 1H, *J* = 2.6, 12.3 Hz), 5.15 – 5.28 (m, 2H), 5.40 (dd, 1H, *J* = 2.3, 5.1 Hz).

^{13}C NMR (100 MHz, CD_3OD) δ 20.6, 20.8, 20.8, 20.9, 22.7, 38.0, 50.5, 53.3, 63.6, 69.8, 71.1, 71.6, 72.7, 96.3, 170.7, 171.8, 172.0, 172.0, 172.5, 173.4.

HRMS (ESI+) *m/z*: calculation for $[C_{20}H_{29}NNaO_{13}]^+$ 514.1531; found 514.1534.

Compound (**5**)[14]

To a mixture of compound (**4**) (3.4 g, 6.9 mmol), triphenyl-phosphine (5.3 g, 20.2 mmol) and thymol (3.0 g, 20.0 mmol) in anhydrous acetonitrile (50 mL), DEAD (3.1 mL, 6.9 mmol, 40% solution in tetrahydrofuran (THF)) was added slowly at -5 °C. The reaction mixture was stirred for 1 hour at 0 °C and for 4 days at room temperature. After removing the solvent, the crude compound was purified by column chromatography (silica gel; 33-100% ethyl acetate in cyclohexane) to yield compound (**5**) as colorless solid (3.4 g, 5.5 mmol, 80%).

HRMS (ESI+) *m/z:* calculation for $[C_{30}H_{41}NNaO_{13}]^+$ 646.24701; found 646.24587.

Sensor (**6**)[15]

To a solution of compound (**5**) (3.3 g, 5.3 mmol) in anhydrous methanol (30 mL) sodium methoxide solution (314 µL, 1.7 mmol, 5.4 M) was added. After stirring 45 min at room temperature the solution was neutralized with Amberlite® IRC 120H H^+ form, filtered and the solvent was evaporated under reduced pressure to yield compound (**6-1**) as colorless solid (2.2 g, 4.8 mmol, 91%).
HRMS (ESI-) *m/z*: calculation for $[C_{22}H_{32}NO_9]^-$ 454.20826; found 454.20820.

A mixture of compound (**6-1**) (1.1 g, 2.4 mmol) and lithium hydroxide monohydrate (441 mg, 10.5 mmol) in a mixture of water and methanol (28 mL, 4:1) was stirred for 2.5 hours at room temperature. After removing the solvent, the crude product was purified by flash chromatography (RP-18 Silica, acetonitrile + 0.1FA, water + 0.1FA) to yield α-sensor (**6**) (127 mg, 0.3 mmol, 12%), β-sensor (**6**) (90 mg, 0.2 mmol, 10%) and α/β-sensor (**6**)-mixture (15 mg, 0.03 mmol, 2%).

α-sensor (**6**)

^{1}H NMR (400 MHz, CD_3OD) δ 1.11 – 1.22 (m, 6H), 1.93 – 2.06 (m, 4H), 2.26 (s, 3H), 2.83 (dd, 1H, *J* = 4.4, 12.7), 3.32 – 3.38 (m, 1H), 3.54 – 3.63 (m, 1H), 3.65 – 3.91 (m, 5H), 3.91 – 4.01 (m, 1H), 6.86 (d, 1H, *J* = 7.8 Hz), 7.09 (d, 1H, *J* = 7.9 Hz), 7.26 (s, 1H).

^{13}C NMR (100 MHz, CD_3OD) δ 21.1, 22.6, 23.4, 23.8, 27.5, 41.6, 53.7, 64.6, 68.7, 70.0, 73.2, 75.8, 102.4, 123.3, 126.1, 126.9, 137.1, 138.7, 153.3, 171.1, 175.4.

HRMS (ESI-) *m/z*: calculation for $[C_{21}H_{30}NO_9]^-$ 440.19261; found 440.19474.

*Synthesis of Methylated Sensor (**15**)*

Compound (**7**)[16]

Conventional Synthesis

A suspension of *N*-acetylneuraminic acid (10.0 g, 32.3 mmol) and Amberlite® IRC 120H H^+ form resin (10.0 g) in anhydrous methanol (400 mL) was refluxed with vigorous stirring for 5 days at 70 °C. After this period, additional 5 g of Amberlite® IRC 120H H^+ form resin was added, and the suspension was stirred for a further 2 days at 70 °C. The resin was filtered off and washed exhaustively with methanol. The solvent was removed under reduced pressure. The crude product was redissolved in methanol and kept at -20 ˚C overnight for crystallization. The crystals were collected and washed with cold ethyl acetate **:** methanol (6 **:** 1) to provide compound (**7**) as colorless crystals (4.3 g, 12.7 mmol, 39%).

^{1}H NMR (400 MHz, CD_3OD) δ 1.64 (dd, 1H, *J* = 12.8, 11.3 Hz), 2.00 (s, 3H), 2.34 (dd, 1H, *J* = 12.9 Hz, 4.9 Hz), 3.27 (s, 3H), 3.50 (d, 1H, *J* = 9.0 Hz), 3.62 – 3.69 (m, 1H), 3.78 – 3.86 (m, 7H), 3.95 – 4.04 (m, 1H).

^{13}C NMR (100 MHz, CD_3OD) δ 22.7, 41.6, 51.6, 53.1, 53.8, 65.3, 67.7, 70.2, 71.4, 72.3, 100.4, 170.8, 174.8.

HRMS (ESI+) *m/z*: calculation for $[C_{13}H_{23}NNaO_9]^+$ 360.12650; found 360.12612.

Conventional synthesis with modification leading to higher product yield

A suspension of *N*-acetylneuraminic acid (10.0 g, 32.3 mmol) and Amberlite® IRC 120H H^+ form resin (15.0 g) in anhydrous methanol (400 mL) was refluxed with vigorous stirring for 3 days at 70 °C. The resin was removed by hot filtration and washed exhaustively with methanol. After removing the solvent, the crude product was redissolved in methanol and kept at -20 ˚C overnight for crystallization. The crystals were collected and washed with a cold ethyl acetate **:** methanol mixture of 6 **:** 1 to yield compound (**7**) as colorless crystals (6.1 g, 18.0 mmol, 56%).

^{1}H NMR (400 MHz, CD_3OD) δ 1.64 (dd, 1H, *J* = 12.9, 11.2 Hz), 2.00 (s, 3H), 2.35 (dd, 1H, *J* = 12.9 Hz, 4.9 Hz), 3.27 (s, 3H), 3.50(d, 1H, *J* = 9.0 Hz), 3.62 – 3.69 (m, 1H), 3.79 – 3.86 (m, 7H), 3.96 – 4.05 (m, 1H).

^{13}C NMR (100 MHz, CD_3OD) δ 22.7, 41.6, 51.6, 53.1, 53.8, 65.3, 67.7, 70.2, 71.4, 72.3, 100.4, 170.8, 174.8.

Microwave-Assisted Synthesis

A suspension of *N*-acetylneuraminic acid (4.0 g, 12.9 mmol) and Amberlite® IRC 120H H^+ form resin (6.0 g) in anhydrous methanol (160 mL) was refluxed using microwave method 1. After the resin was filtered off hot and washed exhaustively with methanol, the solvent was removed under reduced pressure. The crude product was recrystallized from methanol at -20 °C. The crystals were collected and washed with a cold ethyl acetate : methanol mixture of 6 : 1 to yield compound (**7**) as colorless crystals (2.3 g, 6.8 mmol, 52%).

^1H NMR (400 MHz, CD_3OD) δ 1.64 (dd, 1H, *J* = 12.9, 11.3 Hz), 2.00 (s, 3H), 2.34 (dd, 1H, *J* = 12.9 Hz, 4.9 Hz), 3.27 (s, 3H), 3.47 – 3.54 (m, 1H), 3.62 – 3.69 (m, 1H), 3.77 – 3.85 (m, 7H), 3.95 – 4.04 (m, 1H).

^{13}C NMR (100 MHz, CD_3OD) δ 22.7, 41.6, 51.6, 53.1, 53.8, 65.3, 67.7, 70.2, 71.4, 72.3, 100.4, 170.8, 174.8.

A suspension of *N*-acetylneuraminic acid (10.0 g, 32.3 mmol) and Amberlite® IRC 120H H^+ form resin (20.0 g) in anhydrous methanol (210 mL) was refluxed with vigorous using microwave method 3. Reaction progress was monitored by IR-spectroscopy. After the resin was filtered off and washed exhaustively with methanol, the solvent was removed under reduced pressure. Crude compound (**7**) was obtained as orange solid (6.6 g, 19.6 mmol, 61%). It was possible to synthesize compound (**8**) with crude compound (**7**).

Compound (**8**)[16]

A mixture of compound (**7**) (2.5 g, 7.4 mmol) and camphor sulfonic acid (86 mg, 0.4 mmol) in anhydrous acetone (34 mL) was stirred at room temperature for 3 hours. After neutralizing the solution with triethylamine, it was evaporated to dryness. The crude product was purified by silica gel column chromatography (dichloromethane : methanol : triethylamine = 92 : 8 : 0.1) to give compound (**8**) as a colorless powder (2.5 g, 6.6 mmol, 89%).

^1H NMR (400 MHz, CD_3OD) δ 1.29 (s, 3H), 1.35 (s, 3H), 1.59 – 1.69 (m, 1H), 2.01 (s, 3H), 2.35 (dd, 1H, *J* = 12.9, 4.9 Hz), 3.25 (s, 3H), 3.47 (d, 1H, *J* = 8.5 Hz), 3.66 – 3.73 (m, 1H), 3.79 – 3.85 (m, 4H), 3.91 – 4.04 (m, 2H), 4.07 – 4.15 (m, 1H), 4.22 – 4.31 (m, 1H).

^{13}C NMR (100 MHz, CD_3OD) δ 22.7, 25.6, 27.3, 41.5, 51.5, 53.2, 53.6, 67.5, 68.6, 71.5, 72.6, 75.8, 100.4, 110.2, 170.8, 174.8.

HRMS (ESI+) *m/z*: calculation for $[C_{16}H_{27}NNaO_9]^+$ 400.15780; found 400.15692.

Compound (**9**)[16]

A mixture of compound (**8**) (2.0 g, 5.3 mmol), barium oxide (3.4 g, 21.9 mmol) and barium hydroxide octahydrate (348 mg, 1.1 mmol) in anhydrous N,N-dimethylformamide (35 mL) was stirred at room temperature. After 20 min, methyl iodide (3.5 mL, 56.3 mmol) was added, and the suspension was kept stirring for 20 hours at room temperature. The solids were filtered off and washed exhaustively with ethyl acetate.

The filtrate was concentrated under reduced pressure. Brine (100 mL) was added, and the aqueous phase was extracted with ethyl acetate (3*100 mL). The organic layer was dried over anhydrous Na_2SO_4, then evaporated under reduced pressure and lyophilized. The crude product was recrystallized from acetone : n-hexane (2 : 1) to give compound (**9**) as colorless crystals (1.4 g, 3.3 mmol, 63%).

^{1}H NMR (400 MHz, CD_3OD) δ 1.30 (s, 3H), 1.39 (s, 3H), 1.52 (dd, 1H, *J* = 11.3, 12.8 Hz), 1.98 (s, 3H), 2.46 (dd, 1H, *J* = 4.8, 12.9 Hz), 3.22 (s, 3H), 3.33 (s, 3H), 3.44 – 3.55 (m, 4H), 3.51 – 3.67 (m, 1H), 3.74 (dd, 1H, *J* = 1.5, 10.6 Hz) 3.78 (s, 3H), 3.95 – 4.08 (m, 2H), 4.08 – 4.16 (m, 1H), 4.20 – 4.31 (m, 1H).

^{13}C NMR (100 MHz, CD_3OD) δ 23.0, 25.5, 26.9, 37.7, 51.2, 51.5, 52.9, 57.9, 61.7, 67.3, 73.3, 77.7, 78.1, 79.8, 100.3, 109.4, 169.6, 173.3.

HRMS (ESI+) *m/z*: calculation for $[C_{18}H_{31}NNaO_9]^+$ 428.1891; found 428.1908.

Compound (**10**)[16]

To a mixture of compound (**9**) (2.8 g, 6.8 mmol) in a mixture of water and methanol (28 mL, 1:1), 1 M NaOH (14 mL) was added.

After stirring for 3 hours at room temperature, the mixture was neutralized by adding Amberlite® IRC 120H H^+ form resin. The resin was filtered off and washed with methanol and water. The filtrate was evaporated to dryness and lyophilized to give crude compound (**10-1**) as a colorless powder (2.6 g, 6.7 mmol, 98%).

^{1}H NMR (400 MHz, CD_3OD) δ 1.31 (s, 3H), 1.39 (s, 3H), 1.54 (dd, 1H, *J* = 11.2, 12.8 Hz), 1.99 (s, 3H), 2.47 (dd, 1H, *J* = 4.8, 12.9 Hz), 3.24 (s, 3H), 3.34 (s, 3H), 3.41 – 3.47 (m, 1H), 3.53 (s, 3H), 3.58 – 3.66 (m, 1H), 3.72 – 3.79 (m, 1H), 3.94 – 4.05 (m, 2H), 4.09 – 4.19 (m, 1H), 4.22 – 4.32 (m, 1H).

^{13}C NMR (100 MHz, CD_3OD) δ 22.9, 25.6, 27.0, 37.9, 51.3, 51.4, 57.1, 61.8, 67.7, 72.9, 77.0, 77.9, 80.3, 101.1, 109.7, 172.5, 173.4.

HRMS (ESI-) *m/z*: calculation for $[C_{17}H_{28}NO_9]^-$ 390.17696; found 390.17743.

Conventional Synthesis of compound (**10**)

Compound (**10-1**) (1.3 g, 3.3 mmol) was treated with 30 mM HCl (27 mL) and Amberlite® IRC 120H H^+ form resin (1.3 g) and refluxed with vigorous stirring for 2 days at 70 °C. The resin was filtered off and

washed with water. The solvent was evaporated to dryness and lyophilized to give crude compound (**10**) (1.0 g, 3.1 mmol, 94%).

^{1}H NMR (100 MHz, CD_3OD) δ 1.75 (dd, 1H, *J* = 11.6, 12.7 Hz), 2.03 (s, 3H), 2.35 (dd, 1H, *J* = 4.7, 12.8 Hz), 3.33 – 3.37 (m, 4H), 3.44 (s, 3H), 3.58 – 3.71 (m, 2H), 3.72 – 3.81 (m, 2H), 4.00 – 4.09 (m, 1H), 4.12 – 4.22 (m, 1H).

^{13}C NMR (100 MHz, CD_3OD) δ 22.7, 36.8, 51.9, 57.1, 60.8, 64.0, 71.8, 71.9, 78.2, 79.5, 96.5, 171.4, 173.8.

HRMS (ESI-) *m/z*: calculation for $[C_{13}H_{22}NO_9]^-$ 336.12945; found 336.13204.

Microwave-Assisted Synthesis of compound (**10**)

Compound (**10-1**) (1.7 g, 4.3 mmol) was treated with 30 mM HCl (20 mL) and Amberlite® IRC 120H H^+ form resin (1.1 g) and refluxed with vigorous stirring using microwave method 2. The resin was filtered off and washed with water. The solvent was evaporated to dryness and lyophilized to give crude compound (**10**) (1.4 g, 4.0 mmol, 94%).

^{1}H NMR (400 MHz, CD_3OD) δ 1.77 (dd, 1H, *J* = 11.5, 12.7 Hz), 1.99 (s, 3H), 2.34 (dd, 1H, *J* = 4.7, 12.8 Hz), 3.33 – 3.37 (m, 4H), 3.44 (s, 3H), 3.58 – 3.70 (m, 2H), 3.71 – 3.81 (m, 2H), 3.99 – 4.09 (m, 1H), 4.11 – 4.19 (m, 1H).

^{13}C NMR (100 MHz, CD_3OD) δ 22.9, 36.8, 51.7, 57.1, 60.9, 64.0, 72.0, 78.4, 79.6, 96.6, 171.4, 173.4.

Compound (**11**)[16]

A suspension of compound (**10**) (1.3 g, 3.9 mmol) and Amberlite® IRC 120H H^+ form resin (1.3 g) in anhydrous methanol (20 mL) was stirred for 5 hours at room temperature. The resin was filtered off, washed with methanol and the filtrate was evaporated to dryness and lyophilized to give crude compound (**11**) (1.1 g, 3.0 mmol, 78%).

^{1}H NMR (400 MHz, CD_3OD) δ 1.78 (dd, 1H, *J* = 11.5, 12.8 Hz), 2.0 (s, 3H) 2.36 (dd, 1H, *J* = 4.7, 12.8 Hz), 3.36 – 3.39 (m, 4H), 3.46 (m, 3H), 3.60 -3.71 (m, 2H), 3.73 – 3.84 (m, 5H), 4.00 – 4.11 (m, 1H), 4.14 – 4.21 (m, 1H).

^{13}C NMR (100 MHz, CD_3OD) δ 22.9, 36.8, 51.7, 53.1, 57.1, 60.9, 64.0, 72.0, 78.4, 79.6, 96,6, 171.4, 173.4.

Compound (**12**)[11, 12]

Compound (**11**) (1.0 g, 2.8 mmol) was treated with Ac_2O (4.0 mL, 42.3 mmol) and anhydrous pyridine (3.8 mL, 47.1 mmol) and stirred overnight at room temperature. After 20 hours the solvent was removed

under reduced pressure. The crude product was purified by flash chromatography (silica gel, 100% ethyl acetate) to give crude compound (**12**) (1.0 g, 2.1 mmol, 75%).

^{1}H NMR (400 MHz, CD_3OD) δ 1.67 (dd, 1H, *J* = 11.2, 13.3 Hz), 1.99 (s, 3H), 1.99 – 2.00 (m, 6H), 2.10 (s, 3H), 2.57 (dd, 1H, *J* = 4.7, 13.3 Hz), 3.36 (s, 3H), 3.48 (s, 3H), 3.52 – 3.56 (m, 1H), 3.65 – 3.72 (m, 1H), 3.74 (s, 3H), 3.83 – 3.89 (m, 1H), 3.97 – 4.03 (m, 1H), 4.13 – 4.19 (m, 1H), 4.66 (dd, 1H, *J* = 2.4, 12.4 Hz), 4.97 – 5.05 (m, 1H)

^{13}C NMR (100 MHz, CD_3OD) δ 20.6, 20.7, 21.0, 23.0, 36.7, 51.2, 53.4, 57.4, 61.4, 63.7, 74.1, 74.5, 77.1, 78.7, 99.0, 168.8, 170.0, 171.7, 172.5, 173.4.

HRMS (ESI+) *m/z*: calculation for $[C_{20}H_{31}NNaO_{12}]^+$ 500.17385; found 500.17313.

Compound (**13**)[13]

Compound (**12**) (580 mg, 1.2 mmol) was dissolved in anhydrous dichloromethane (10 mL) and cooled to 0 °C. AcCl (1.5 mL, 21.1 mmol) and HCl (50 µL, 0.6 mmol) were added, and the reaction mixture was stirred for 10 min at 0 °C. After stirring overnight at room temperature dichloromethane (20 mL) was added, and the reaction mixture was extracted with water (30 mL), saturated $NaHCO_3$ solution (30 mL) and brine (30 mL). The organic layer was dried over anhydrous magnesium sulfate ($MgSO_4$) and evaporated under reduced pressure to give crude compound (**13-1**) (480 mg, 1.1 mmol, 87%).

HRMS (ESI-) *m/z*: calculation for $[C_{18}H_{27}ClNO_{10}]^-$ 452.1329; found 452.1349 and calculation for $[C_{18}H_{28}Cl_2NO_{10}]^-$ 488.1096; found 488.1113.

A solution of compound (**13-1**) (689 mg, 1.5 mmol) in a mixture of water and acetonitrile (26 mL, 1:1) was stirred for 3 hours at room temperature. After removing the solvent, the crude product was purified by silica gel chromatography (dichloromethane : methanol : triethylamine = 92:8:0.1) to yield compound (**13**) as colorless solid (610 mg, 1.4 mmol, 92%).

^{1}H NMR (400 MHz, CD_3OD) δ 1.71 (dd, 1H, *J* = 11.6, 12.6 Hz), 1.98 (s, 3H), 2.02 (s, 3H), 2.06 (s, 3H), 2.34 (dd, 1H, *J* = 6.7, 12.8 Hz), 3.34 (s, 3H), 3.47 (s, 3H), 3.51 – 3.55 (m, 1H), 3.63 – 3.69 (m, 1H), 3.76 (s, 3H), 3.94 – 4.03 (m, 2H), 4.20 – 4.28 (m, 1H), 4.66 – 4.73 (m, 1H), 5.09 – 5.16 (m, 1H).

^{13}C NMR (100 MHz, CD_3OD) δ 20.7, 21.0, 22.9, 37.0, 51.6, 53.1, 57.0, 61.3, 64.1, 73.1, 75.0, 77.9, 79.6, 96.5, 171.2, 172.1, 172.6, 173.3.

HRMS (ESI+) *m/z*: calculation for $[C_{18}H_{29}NNaO_{11}]^+$ 458.16328; found 458.16229.

Reaction with polymer-supported triphenyl-phosphine beads (referred to as synthesis approach (i) in the main text)

Compound (**14**)[14]

To a mixture of compound (**13**) (950 mg, 2.2 mmol), polymer-supported triphenyl-phosphine beads (4.1 g, 6.6 mmol, 1.6 mmol/g) and thymol (990 mg, 6.6 mmol) in anhydrous acetonitrile (30 mL), DEAD (2.9 mL, 6.4 mmol, 40% solution in THF) was added slowly at -5 °C. The reaction mixture was stirred for 1 hour at 0 °C and for 18.5 hours at room temperature. After removing the solvent, the crude product was purified by column chromatography (silica gel, 25-100% ethyl acetate in cyclohexane) to yield compound (**14**) as colorless solid (Batch 1: 270 mg, 0.5 mmol, 22%, Batch 2: 90 mg, 0.2 mmol, 7% overall yield: 29%)

HRMS (ESI+) *m/z*: calculation for $[C_{28}H_{41}NNaO_{11}]^+$ 590.25718; found 590.25738 and by-product calculation for $[C_{34}H_{51}N_3NaO_{15}]^+$ 764.32124 found 764.32157.

Sensor (**15**)[15]

To a solution of compound (**14**) (270 mg, 475.7 µmol) in anhydrous methanol (30 mL) sodium methoxide solution (30 µL, 0.16 mmol, 5.4 M) was added. After stirring 45 min at room temperature the solution was neutralized with Amberlite® IRC 120H H^+ form resin filtered and the solvent was evaporated under reduced pressure to give crude compound (**15-1**) (220 mg, 455.0 µmol, 96%).

A mixture of compound (**15-1**) (170 mg, 351.6 µmol) and lithium hydroxide monohydrate (64 mg, 1.5 mmol) in a mixture of water and methanol (10 mL, 4:1) was stirred for 1.5 hours at room temperature. After removing the solvent, the crude product (136 mg, 289.7 µmol, 82%) was purified by flash chromatography (RP-18 Silica, 26 g column, acetonitrile + 0.1FA, water + 0.1FA, 5-95% acetonitrile in water) to yield α-sensor (**15**) (3.6 mg, 7.7 µmol, 2%). Due to the contamination with the by-product, it was not possible to isolate β-sensor (**15**) purely.

^{1}H NMR (400 MHz, CD_3OD) δ 1.10 – 1.21 (m, 6H), 1.82 (pseudo t, 1H, *J* = 12.1, 12.2 Hz), 1.97 (s, 3H), 2.24 (s, 3H), 2.86 (dd, 1H, *J* = 4.4, 12.6 Hz), 3.32 – 3.44 (m, 6H), 3.48 (s, 3H), 3.65 – 3.72 (m, 1H), 3.83 – 3.90 (m, 1H), 3.91 – 3.98 (m, 1H), 3.99 – 4.09 (m, 2H), 6.84 (d, 1H, *J* = 7.5 Hz), 7.08 (d, 1H, *J* = 7.9 Hz), 7.24 (s, 1H).

^{13}C NMR (100 MHz, CD_3OD) δ 21.1, 23.0, 23.5, 23.7, 27.5, 37.6, 51.3, 57.3, 61.1, 64.0, 73.1, 75.1, 79.3, 79.6, 102.5, 123.0, 125.8, 126.9, 137.0, 138.6, 152.4, 171.1 (determined using HMBC spectrum), 173.4.

HRMS (ESI-) *m/z*: calculation for $[C_{23}H_{35}NO_9]^-$ 469.23173; found 469.23006.

An alternative reaction with triphenyl-phosphine (not polymer-supported); referred to as synthesis approach (ii) in the main text)

We replaced polymer-supported triphenyl-phosphine with dissolved triphenyl-phosphine. As a result, one impurity, which was observed after the synthesis of compound (**14**) using polymer-supported triphenyl-phosphine, was not detected anymore. Therefore, the purification of sensor (**15**) was facilitated, resulting in substantial yield improvements (α-sensor (**15**) yield increased from about 2% using polymer-supported triphenyl-phosphine to about 31% using dissolved triphenyl-phosphine; β-sensor (**15**) yield increased from non-detectable amounts to about 15%, respectively). Details of this modified reaction scheme were as follows:

Compound (**14**)[14]

Compound (**13**) (500 mg, 1.2mmol), triphenyl-phosphine (452 mg, 1,7 mmol), and thymol (517 mg, 3.4 mmol) were dissolved in anhydrous acetonitrile (9 mL). DEAD (539 µL, 1.2 mmol, 40% solution in THF) was added slowly at -5 °C. The reaction mixture was stirred for 1.5 hours at 0 °C and for 17 hours at room temperature. The solvent was removed, and the crude product was purified by column chromatography (silica gel, 50-100% ethyl acetate in cyclohexane) to yield compound (**14**) as colorless solid (148 mg, 23%).

HRMS (ESI+) *m/z*: calculation for $[C_{28}H_{41}NNaO_{11}]^+$ 590.25718; found 590.25807.

Sensor (**15**)[15]

Compound (**14**) (110 mg, 139.8 µmol) was dissolved in anhydrous methanol (12 mL). Sodium methoxide solution (12 µL, 0.07 mmol, 5.4 M) was added and the solution was stirred for 45 min at room temperature. After neutralizing the solution with Amberlite® IRC 120H H^+ form resin, the solvent was evaporated under reduced pressure to give crude compound (**15-1**) (90 mg, µmol, 96%).

Compound (**15-1**) (90 mg, 186.1 µmol) and lithium hydroxide monohydrate (34 mg, 0.79 mmol) were dissolved in a mixture of water and methanol (5 mL, 4:1). After stirring for 1.5 hours at room temperature and removing the solvent, the crude product was purified by flash chromatography (RP-18 Silica, 26 g column, acetonitrile + 0.1FA, water + 0.1FA, 5-95% acetonitrile in water) to yield α-sensor (**15**) (27 mg, 57.5 µmol, 31%), β-sensor (**15**) (12.8 mg, 27.3 µmol, 15%) and α-β- sensor (**15**) mixture (13 mg, 27.7 µmol, 15%).

α-sensor (**15**)

^1H NMR (400 MHz, CD_3OD) δ 1.10 – 1.21 (m, 6H), 1.77 – 1.88 (m, 1H), 1.97 (s, 3H), 2.24 (s, 3H), 2.85 (dd, 1H, *J* = 4.4, 12.6 Hz), 3.32 – 3.43 (m, 6H), 3.49 (s, 3H), 3.65 – 3.72 (m, 1H), 3.83 – 3.90 (m, 1H), 3.91 – 3.97 (m, 1H), 3.99 – 4.10 (m, 2H), 6.85 (d, 1H, *J* = 7.8 Hz), 7.08 (d, 1H, *J* = 7.8 Hz), 7.24 (s, 1H).

^{13}C NMR (100 MHz, CD_3OD) δ 21.1, 23.0, 23.5, 23.7, 27.5, 37.6, 51.3, 57.3, 61.1, 64.0, 73.1, 75.1, 79.2, 79.6, 102.4, 123.1, 125.9, 126.9, 137.0, 138.6, 152.3, 171.1, 173.4.

HRMS (ESI-) *m/z*: calculation for $[C_{23}H_{34}NO_9]^-$ 468.22391; found 468.22673.

β-sensor (**15**)

^{1}H NMR (400 MHz, CD_3OD) δ 1.21 – 1.29 (m, 6H), 1.71 (dd, 1H, *J* = 11.3, 12.6 Hz), 1.98 (s, 3H), 2.23 (s, 3H), 2.74 (dd, 1H, *J* = 4.6, 12.8 Hz), 3.33 – 3.40 (m, 2H), 3.42 (s, 3H), 3.52 (s, 3H), 3.60 – 3.67 (m, 1H), 3.74 -3.80 (m, 1H), 3.81 – 3.93 (m, 2H), 3.98 – 4.05 (m, 1H), 4.12 – 4.2 (m, 1H), 6.75 (d, 1H, *J* = 7.7 Hz), 7.00 – 7.12 (m, 2H).

^{13}C NMR (100 MHz, CD_3OD) δ 21.3, 22.9, 23.2, 28.2, 39.4, 51.4, 57.3, 60.9, 64.1, 72.2, 73.4, 78.0, 80.1, 101.1, 117.2, 124.0, 126.9, 135.9, 137.3, 153.4, 170.9, 173.5.

Reaction without purification

Compound (**12**) (400 mg, 0.8 mmol) was dissolved in anhydrous dichloromethane (10 mL) and cooled to 0 °C. AcCl (1 mL, 14.1 mmol) and HCl (33 µL, 0.4 mmol) were added and the reaction mixture was stirred for 45 min at 0 °C. After stirring overnight at room temperature dichloromethane (50 mL) was added, and the reaction mixture was extracted with water (30 mL), saturated $NaHCO_3$ solution (30 mL) and brine (30 mL). The organic layer was dried over anhydrous Na_2SO_4 and evaporated under reduced pressure to give crude compound (**13-1**) (200 mg, 0.5 mmol, 57%, HRMS (ESI-) *m/z*: calculation for $[C_{18}H_{23}ClNO_{10}]^-$ 452.13290; found 452.13280). A solution of compound (**13-1**) (200 mg, 0.5 mmol) in a mixture of water and acetonitrile (6 mL, 1:1) was stirred for 3 hours at room temperature. After removing the solvent, crude compound (**13**) obtained as slightly yellow solid (180 mg, 0.4 mmol, 80%, HRMS (ESI+) *m/z*: calculation for $[C_{18}H_{29}NNaO_{11}]^+$ 458.16328; found 458.16162). To a mixture of compound (**13**) (170 mg, 0.39 mmol), triphenyl-phosphine (307 mg, 1.2 mmol) and thymol (176.0 mg, 1.2 mmol) in anhydrous acetonitrile (3 mL), DEAD (184 µL, 0.4 mmol, 40% solution in THF) was added slowly at 0 °C. The reaction mixture was stirred for 1.5 hours at 0 °C and for 48 hours at room temperature. After removing the solvent, the crude compound (**14**) was dissolved in anhydrous methanol (5 mL) and sodium methoxide solution (24 µL, 0.13 mmol, 5.4 M) were added. After stirring 45 min at room temperature the solution was neutralized with Amberlite® IRC 120H H^+ form resin filtered, and the solvent was evaporated under reduced pressure. A mixture of crude compound (**15-1**) and lithium hydroxide monohydrate (83 mg, 2.0 mmol) in a mixture of water and methanol (16 mL, 1:1) was stirred for 1.5 hours at room temperature. After removing the solvent, the crude sensor (**15**) (43 mg, 23%) was purified by flash chromatography (RP-18 Silica, acetonitrile + 0.1FA, water + 0.1FA) to yield α-sensor (**15**) (10 mg, 0.2 mmol, 3%) and β-sensor (**15**). Due to the small quantity, it was not possible to determine the exact yield of β-sensor (**15**).

α-sensor (**15**) HRMS (ESI-) *m/z*: calculation for $[C_{23}H_{34}NO_9]^-$ 468.22391; found 468.22337

β-sensor (**15**) HRMS (ESI-) *m/z*: calculation for $[C_{23}H_{34}NO_9]^-$ 468.22391; found 468.22761

Optimized synthesis strategy (referred to as synthesis approach (iii) in the main text)

Compound (**16**)

Compound (**11**) (1.7 g, 4.7 mmol) and camphor sulfonic acid (55 mg, 0.2 mmol) were dissolved in anhydrous acetone (25 mL) and stirred at room temperature for 5 hours. After neutralizing the solution with triethylamine, and removal of the solvent under reduced pressure, the crude product was purified by silica gel column chromatography (dichloromethane : methanol : triethylamine = 96:4:0.1) to give compound (**16**) as a colorless powder (1.2 g, 3.1 mmol, 67%).

HRMS (ESI+) *m/z*: calculation for $[C_{17}H_{29}NNaO_9]^+$ 414.17345; found 414.17310

Compound (**17**)[14]

To a mixture of compound (**16**) (566 mg, 1.5 mmol), triphenyl-phosphine (651 mg, 2.5 mmol), thymol (569 mg, 3.8 mmol) and molecular sieve 3 Å in anhydrous acetonitrile (11 mL), DEAD (680 µL, 1.5 mmol, 40% solution in THF) was added slowly at -5 °C. The reaction mixture was stirred for 1.5 hours at 0 °C and for 17 hours at room temperature. After filtering out the molecular sieve, the solvent was removed, and the crude product was purified by column chromatography (silica gel, 67-100% ethyl acetate in cyclohexane) to yield compound (**17**) as colorless solid (491 mg, 0.9 mmol, 65%)

HRMS (ESI+) *m/z*: calculation for $[C_{27}H_{41}NNaO_9]^+$ 546.26735; found 546.26965

Compound (**15**)[15]

Compound (**17**) (446 mg, 851.8 µmol) was stirred with Amberlite® IRC 120H (H^+ form, 446 mg) in 30 mM HCl at 35 °C for 3.5 h. The resin was removed by filtration and washed with acetonitrile. The filtrate was concentrated, and the residue was dissolved in water. Insoluble material was filtered off, redissolved, and evaporated to afford crude compound (**15-1**) (217 mg, 448.8 µmol, 53%).

Compound (**15-1**) (217 mg, 448.8 µmol) and lithium hydroxide monohydrate (80 mg, 3.1 mmol) were dissolved in a mixture of water and methanol (10 mL, 4:1) and stirred for 1.5 hours at room temperature. After removing the solvent, water was added, and the pH was adjusted to 2 using formic acid. Amberlite® IRC 120H H+ form resin was then added and stirred for 5 minutes. After the ion exchanger was filtered off, the solvent was removed under reduced pressure. The crude product was purified by flash chromatography (RP-18 Silica, 26 g column, acetonitrile + 0.1FA, water + 0.1FA, 5-95% acetonitrile in water) to yield α-sensor (**15**) (45 mg, 95.8 µmol, 21%).

HRMS (ESI-) *m/z*: calculation for $[C_{23}H_{34}NO_9]^-$ 468.22391; found 468.22635

Supporting Tables

Table S1. Neuraminidase Assay Kit Fluorometric Blue. Columns B, C and E-H represent fluorescence data (Ex/Em = 320/450 nm) corresponding to the activity of three different neuraminidases. Specifically, Columns B and C contain fluorescence values derived from the supplied neuraminidase (standard, n=2) with characterized enzymatic activity (Column A). Columns E-H display fluorescence measurements obtained from viral (n=2) and bacterial (n=2) N, each at defined concentrations (Column D).

Neuraminidase Activity [mU/mL]	Standard		Neuraminidase Concentration [ng/mL]	viral Neuraminidase		bacterial Neuraminidase	
**A**	**B**	**C**	**D**	**E**	**F**	**G**	**H**
20	40051	40668	*0.5*	**28115**	**28942**	41313	41513
10	**30495**	**30734**	*0.25*	14408	15045	42628	41296
5	12621	14513	*0.125*	7097	6926	42109	42875
2.5	4308	5207	*0.0625*	3710	3527	42219	42158
1.25	2283	2373	*0.03125*	2400	2488	40742	39601
0.625	1697	1735	*0.01563*	1890	1968	**30357**	**30206**
0.313	1587	1607	*0.0078*	1759	1690	18249	17913

Table S2. Estimation of neuraminidase activity of H1N1 (A/California/7/2009) ffu/mL. Activity was assessed using viral neuraminidase in combination to 4-MUNANA and a standard curve of 4-methylumbelliferone.

ffu/mL [10^x]	Neuraminidase activity [mU/mL] Mean ± SD
**2**	N/A
**3**	0.32 ± 0.22
**4**	4.12 ± 1.01
**5**	55.55 ± 9.08
**6**	243.08 ± 97.51

Table S3. Comparison of our taste-based screening approach with commonly employed diagnostic techniques.

**Aspect**	**PCR**	**Rapid antigen tests (RIDTs – rapid influenza diagnostic tests)**	**Serological tests**	**Taste-based sensor (15)**
**Specificity**	Very high (>95%).[17]	Very high (95-100%).[18]	Highly dependent on assay type.[19]	High selectivity for viral neuraminidase.
**Sensitivity**	Very high (>95%).[17] Gold-standard, limit of detection around 4.2 x 10^2 plaque forming units (PFU)/ml.[20]	Relatively low (larger risk of false negatives), especially in early stages (50-95%).[18, 20, 21]	Highly dependent on assay type.[19]	Governed by thymol taste threshold (1100 pp).[2, 3] We detected thymol cleavage at 10^3 ffu/mL. For fluorescence-based neuraminidase cleavage assays, a limit of detection of 10^3 PFU/ml was reported before.[20]
**Variability**	May not detect strains possessing novel haemagglutinin or neuraminidase profiles.[22]	High variation depending on test and specimen-related virus isolation.[22, 23]	High variation due to various serological assays available.[19]	More robust against mutations but enzyme activities may differ across strains.[24]
**Time window for application**	≤ 7 days after onset.[25]	≤ 4 days after onset.[26]	Mainly for epidemiological surveillance, only after antibody production.[19]	Late-stage-disease saliva results suggest a time window of ~1-7 days post-infection.
**Time for detection**	Slow (up to several days).[27]	Fast (~15-30 min).[28]	~2 days.[19]	Fast (10-30 min).
**Relative costs**	$$$[29]	$$[30]	NA[22]	$, would not interfere with supply chains of other tests during epidemics.[24]

Supporting Figures

Synthesized Sensors

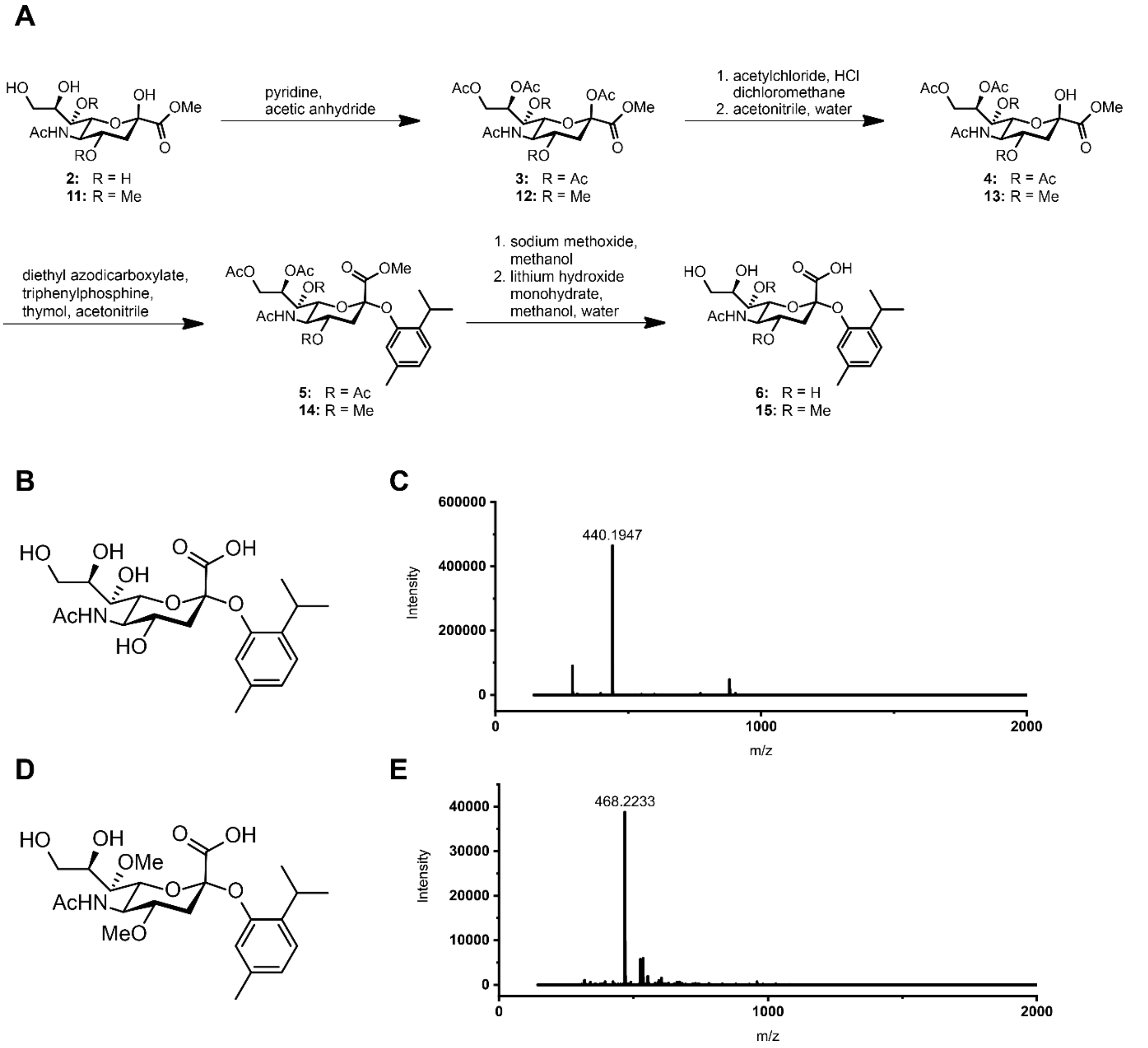

Figure S1. (A) Synthesis scheme of sensors with thymol, (B) unmethylated reference sensor (**6**), (C) HRMS (ESI-) of sensor (**6**) calculation for $[C_{21}H_{30}NO_9]^-$ 440.19261; found 440.19474, (D) sensor (**15**) and (E) HRMS (ESI-) of sensor (**15**) calculation for $[C_{23}H_{34}NO_9]^-$ 468.22391; found 468.22337.

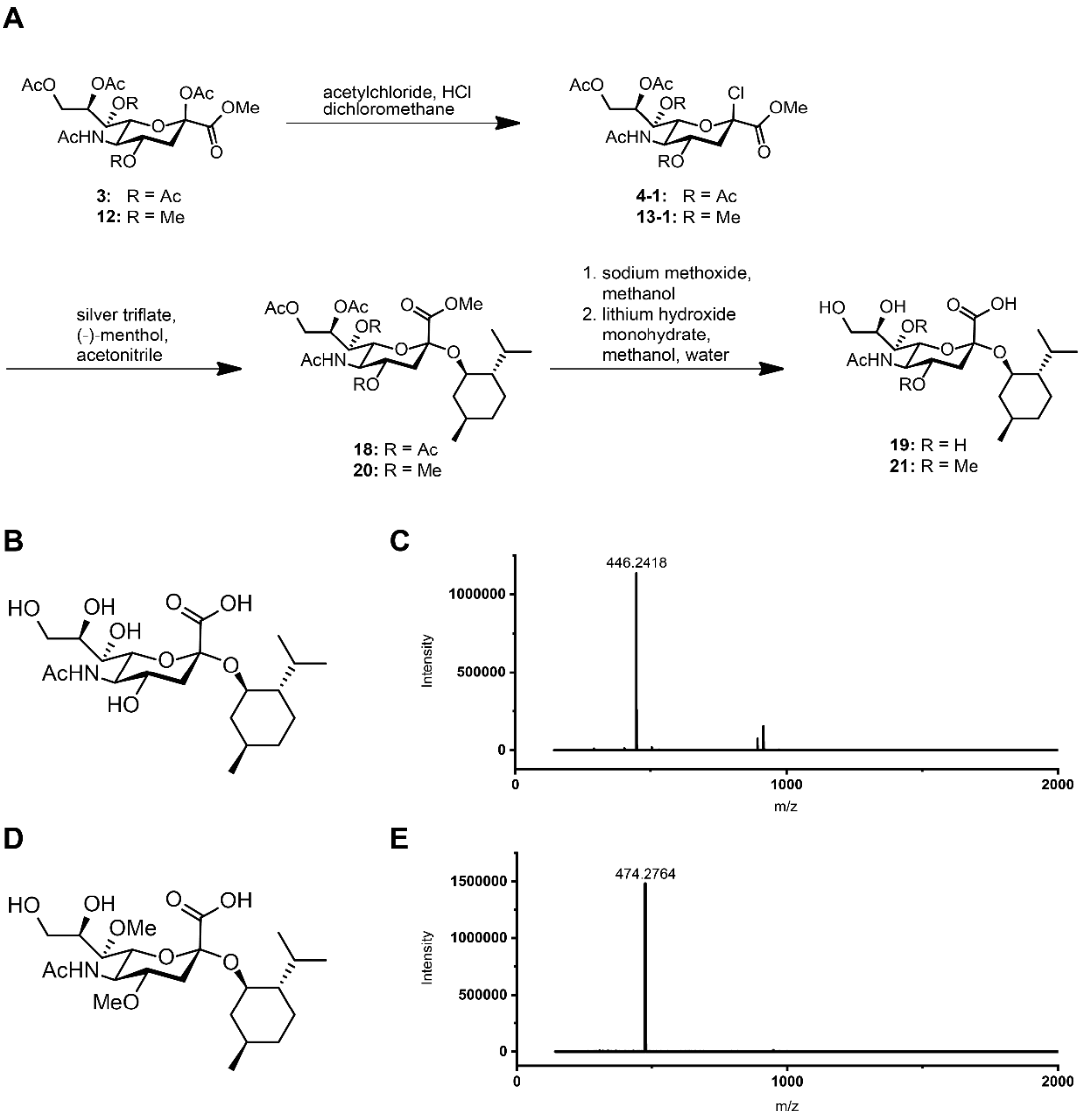

Figure S2. (A) Synthesis scheme of sensors with menthol, (B) unmethylated reference sensor (**19**), (C) HRMS (ESI-) of sensor (**19**) calculation for $[C_{21}H_{36}NO_9]^-$ 446.23956; found 446.24181, (D) sensor (**21**) and (E) HRMS (ESI-) of sensor (**21**) calculation for $[C_{23}H_{40}NO_9]^-$ 474.27085; found 474.27649.

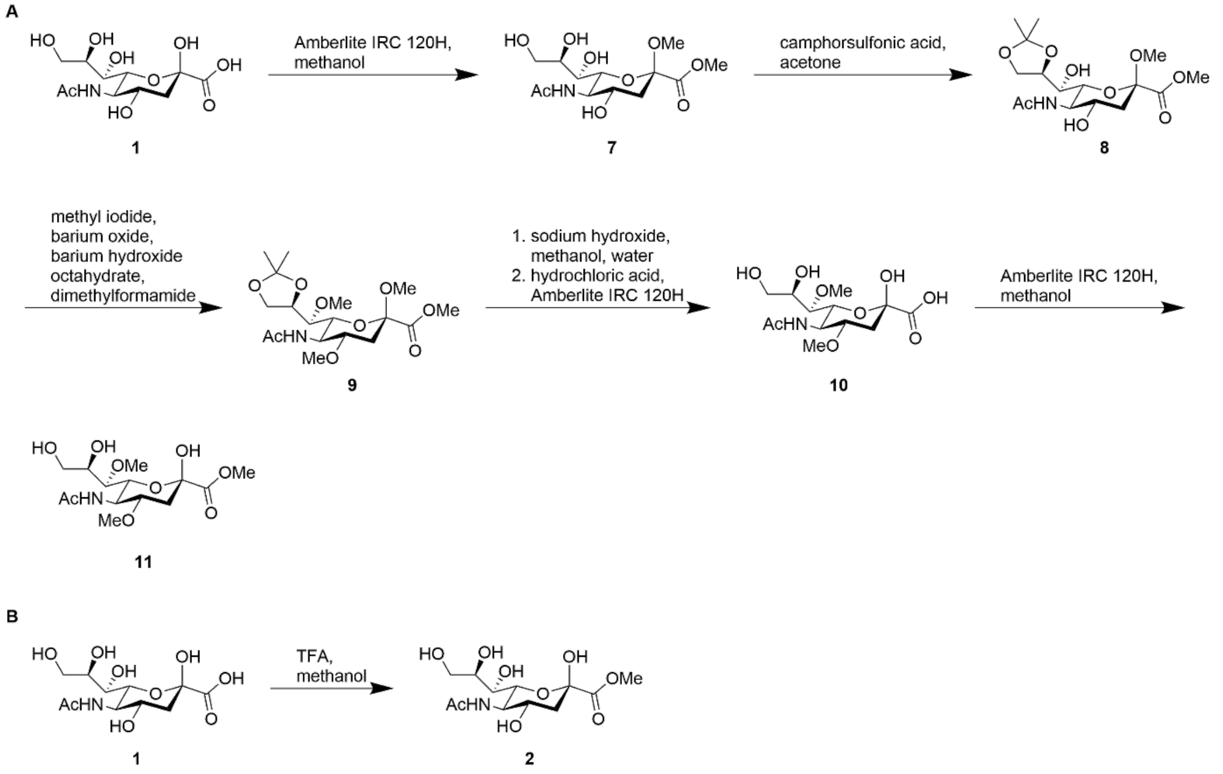

Figure S3. (A) Synthesis of 4,7-di-O-methyl-*N*-acetylneuraminic acid (**10**) with subsequent esterification to compound (**11**). (B) Esterification of *N*-acetylneuraminic acid to compound (**2**).

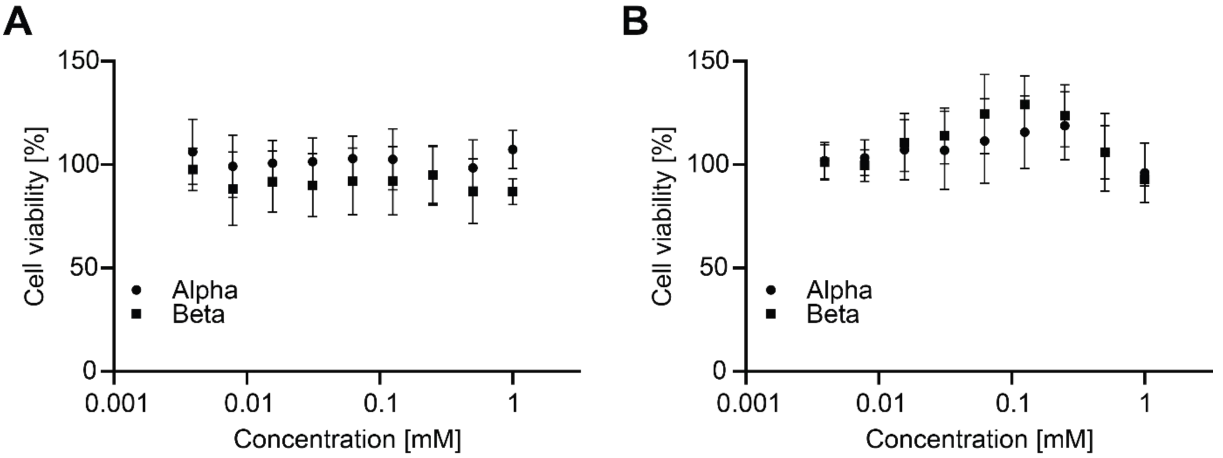

Figure S4. Cytotoxicity assessment of α- and β-sensor (**15**) after 24 h incubation using (A) NIH 3T3 and (B) HEK 293 cell lines. All measurements were performed as biological and technical triplicates (mean ± SD).

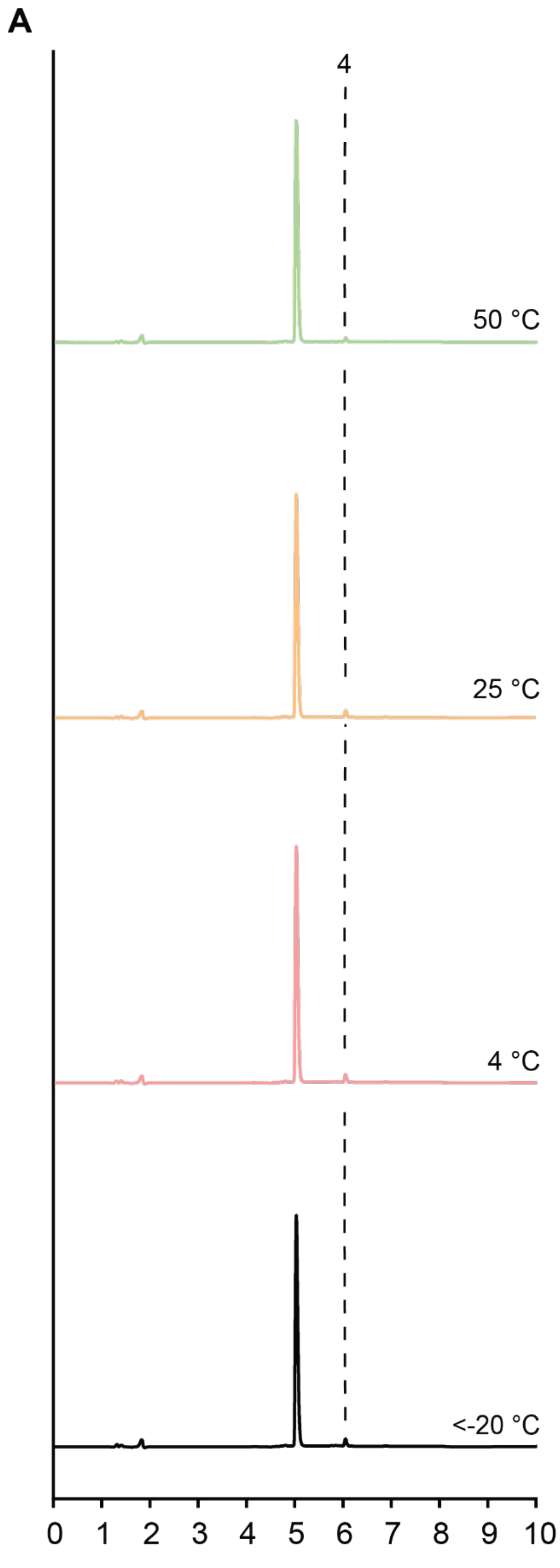

Figure S5. Stability assessment of α-sensor (**15**). (A) α-sensor (**15**) stored at different temperatures over a four-week period. (B) Summary of the stability data for α-sensor (**15**). All measurements were performed as technical triplicates (mean ± SD). (4) Thymol.

Neuraminidase Cleavage Experiments

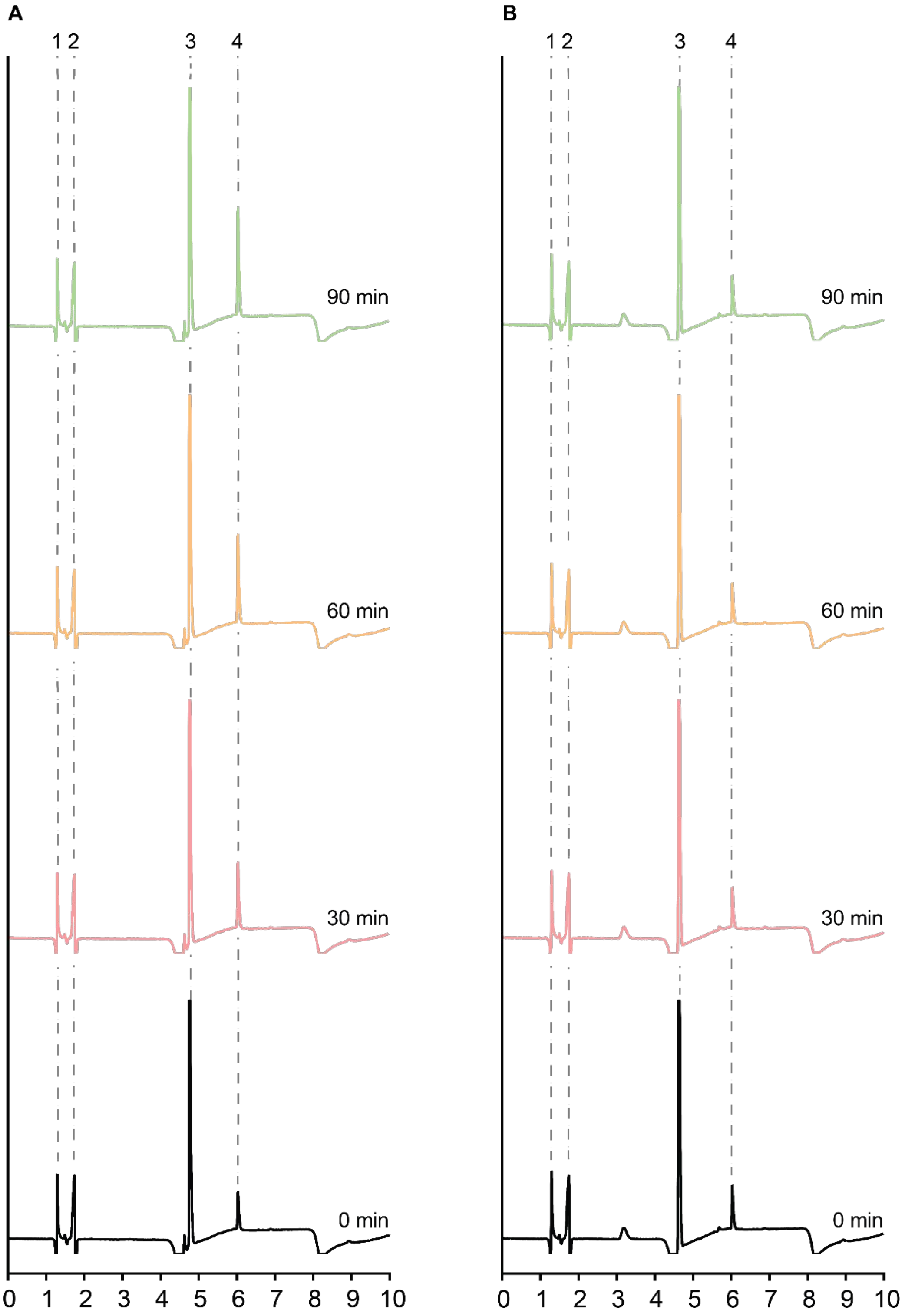

Figure S6. (A) α-Unmethylated reference sensor (**6**) and (B) β-unmethylated reference sensor (**6**) with H1N1 viral neuraminidase in PBS buffer over 90 min. Measurement with β-unmethylated reference sensor (**6**) was conducted in duplicate and the measurement with α-unmethylated reference sensor (**6**) was performed as technical triplicate. (1) and (2) PBS, (3) sensor and (4) thymol.

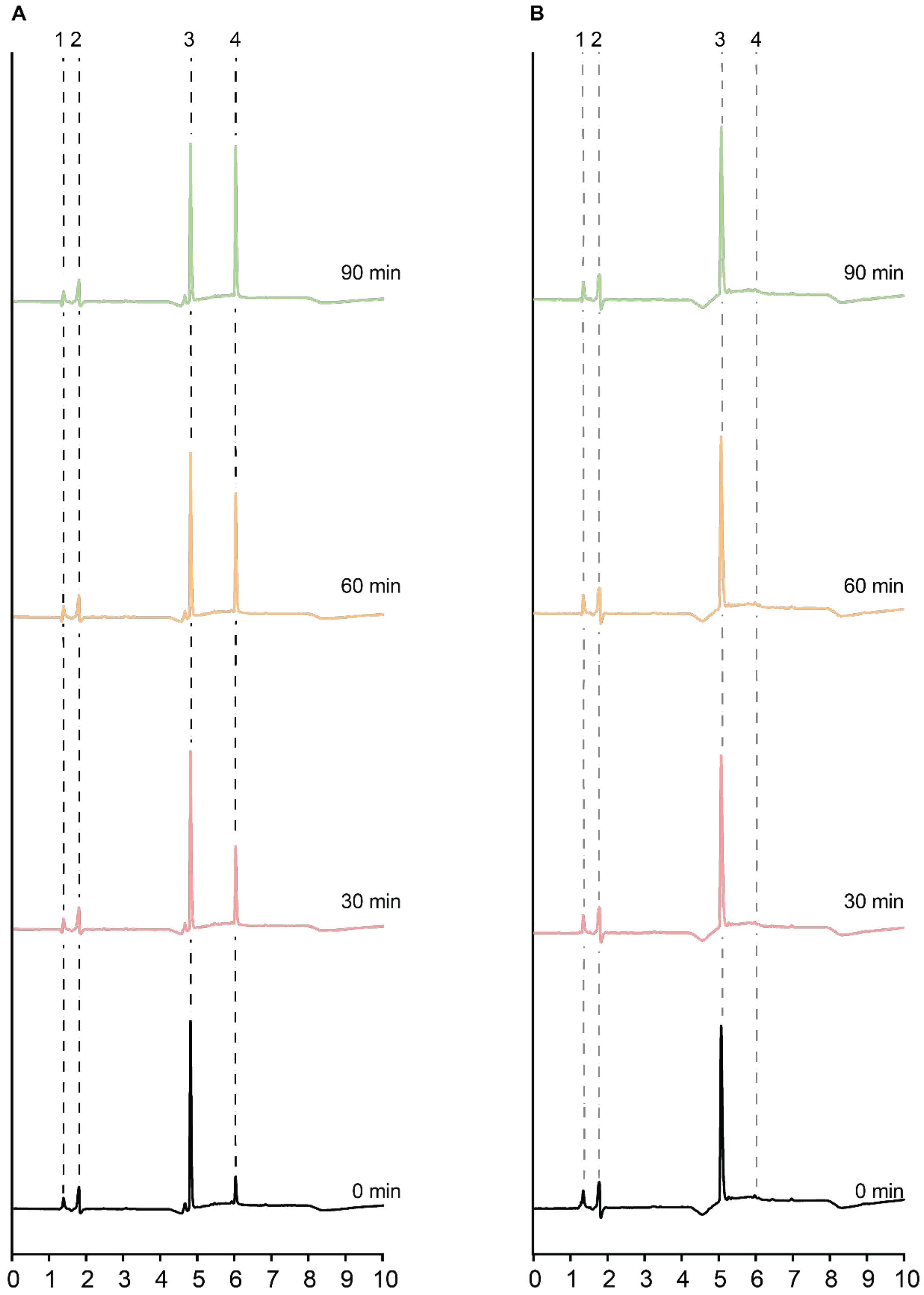

Figure S7. (A) α-Unmethylated reference sensor (**6**) incubated with *M. viridifaciens* neuraminidase in *M. viridifaciens* buffer and (B) α-sensor (15) incubated with *M. viridifaciens* neuraminidase in *M. viridifaciens* buffer over 90 min. Measurements were performed as technical triplicate. (1) and (2) buffer (3) sensor and (4) thymol.

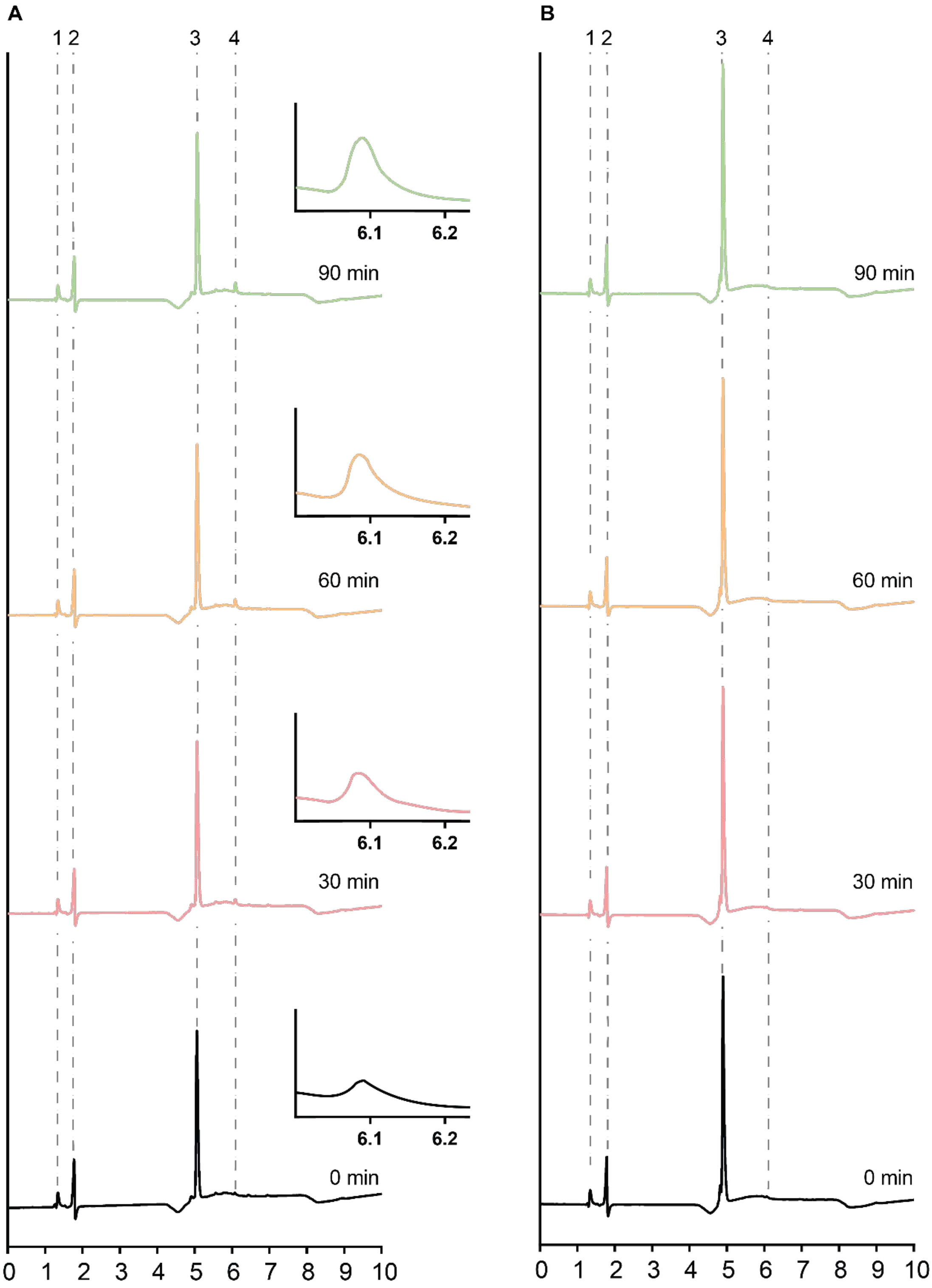

Figure S8. (A) α-Sensor (**15**) and (B) β-sensor (**15**) incubated with viral neuraminidase in PBS buffer over 90 min. Measurements were performed as technical triplicate. (1) and (2) PBS, (3) Sensor and (4) thymol.

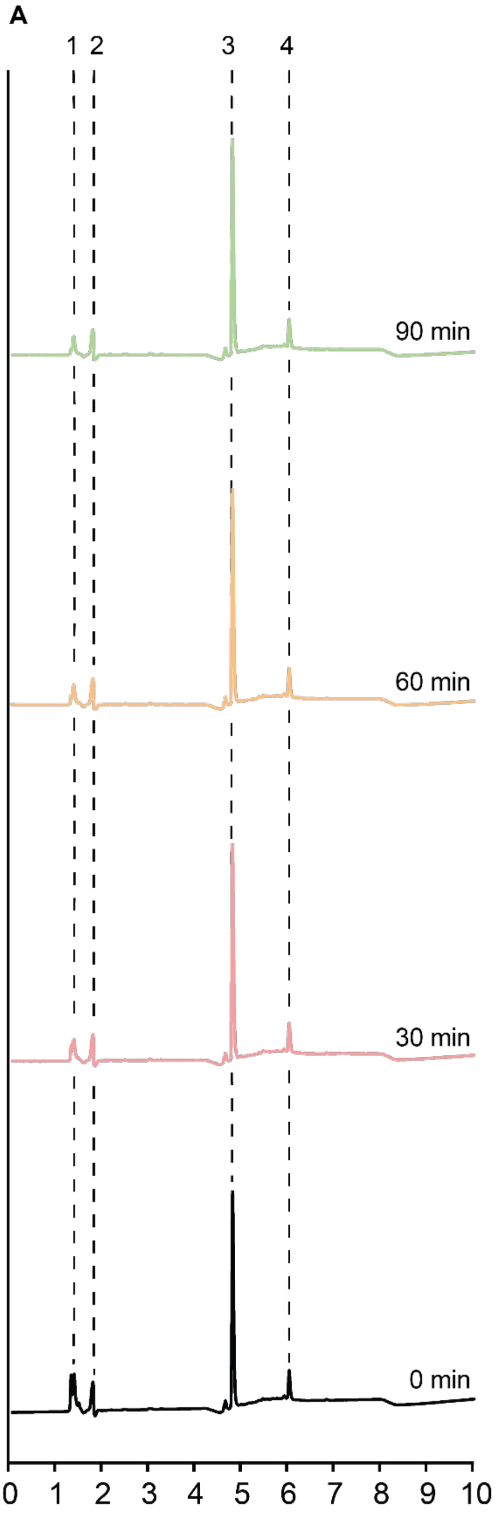

Figure S9. α-Unmethylated reference sensor (**6**) stability in (A) *M. viridifaciens* buffer and (B) PBS buffer. Measurements were performed as technical triplicate. (1) and (2) buffer, (3) sensor and (4) thymol. Residual thymol resulting from the synthesis was found in the starting solution at t = 0 min, notably, the peak area remains constant throughout the investigated period.

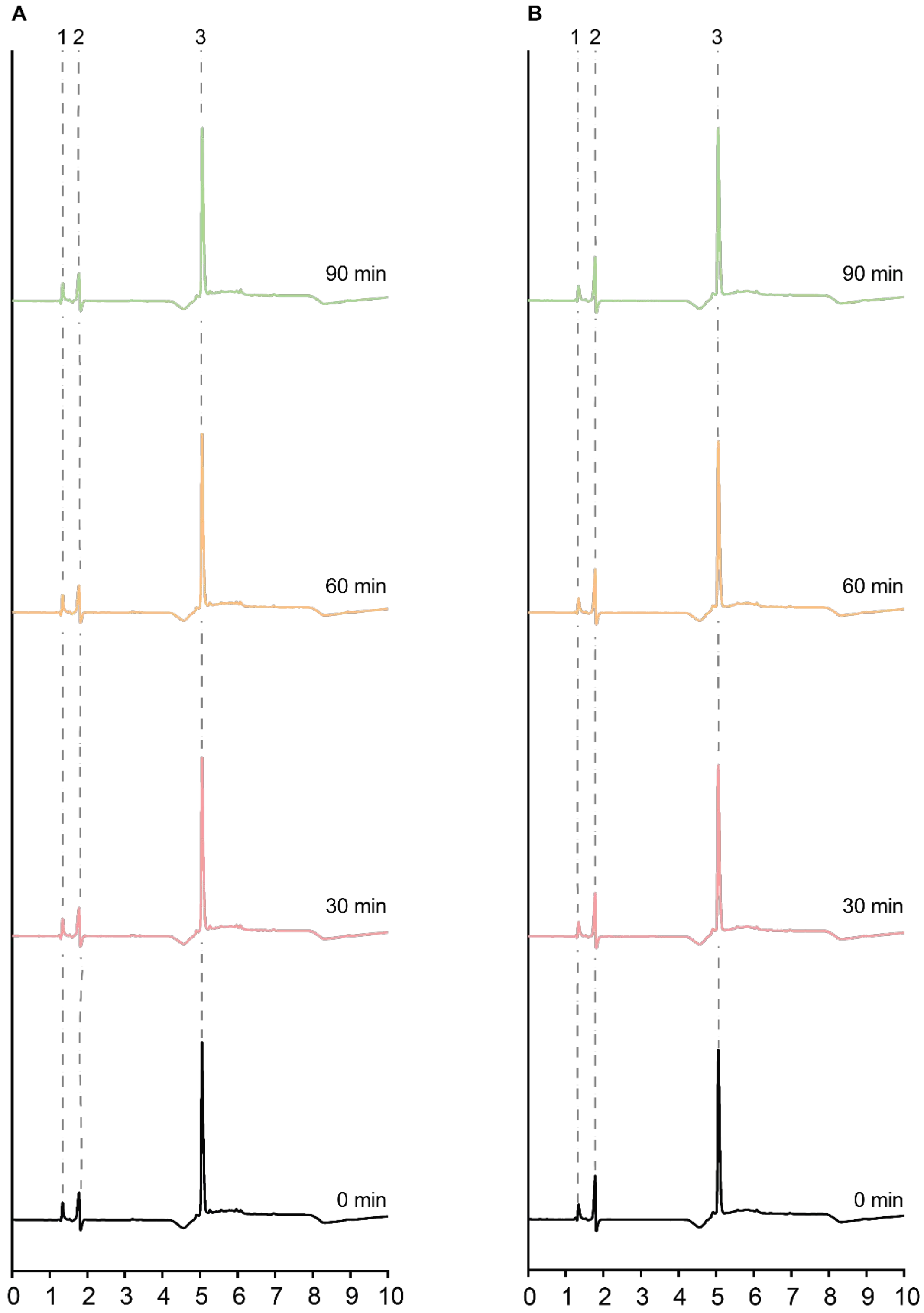

Figure S10. α-Sensor (**15**) stability in (A) *M. viridifaciens* buffer and (B) PBS buffer. Measurements were performed as technical triplicate. (1) and (2) buffer and (3) sensor.

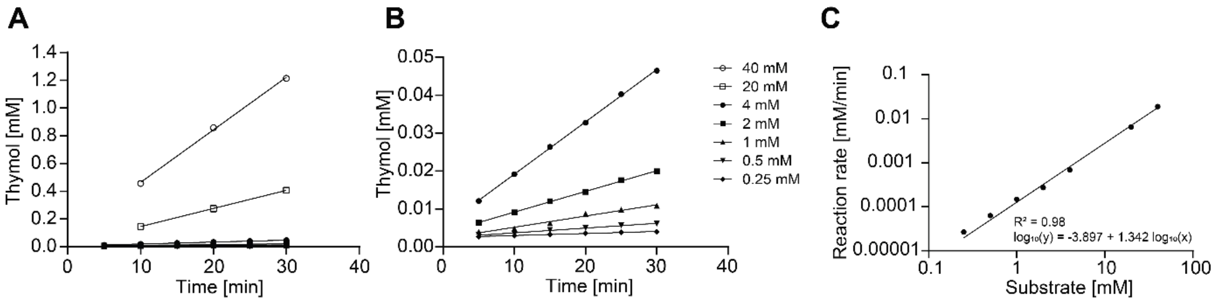

Figure S11: Enzymatic cleavage of the α-sensor (**15**) with 10 mU/mL viral neuraminidase. (A) Full range view of the cleavage at different α-sensor (**15**) concentrations over time. (B) Expanded view of the lower values of the cleavage at different α-sensor (**15**) concentrations over time (C) Reaction rate as a function of substrate concentration. Each sample was measured as technical triplicate (mean ± SD). The thymol standard curve had an $R^2 = 1.00$.

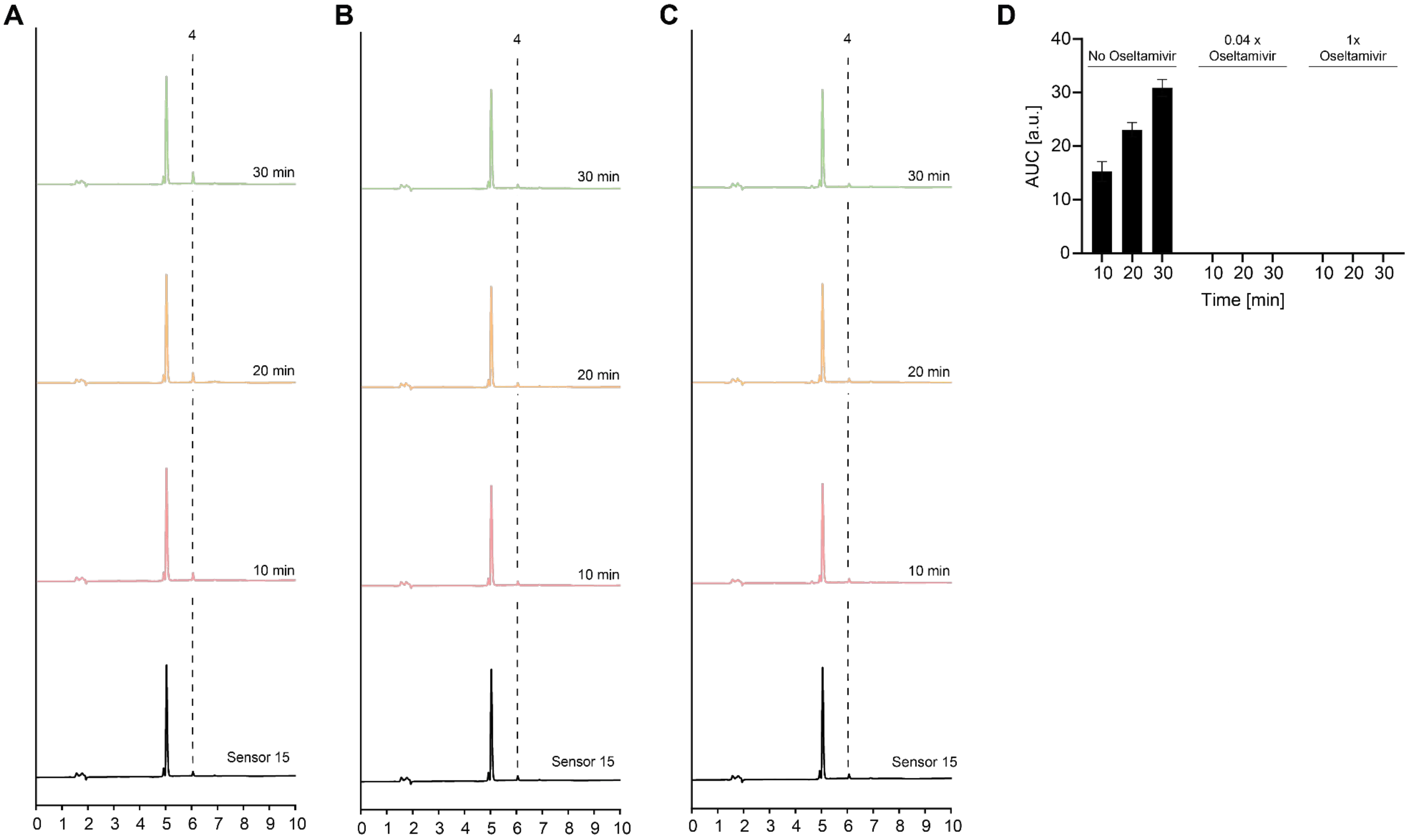

Figure S12. Inhibitor assessment of α-sensor (**15**) using oseltamivir phosphate and 10 mU/mL viral neuraminidase over 30 min. (A) α-sensor (**15**) without inhibitor, (B) α-sensor (**15**) with 0.04x fold oseltamivir phosphate, (C) α-sensor (**15**) with 1x fold oseltamivir phosphate, and (D) summary of inhibition data. All measurements were performed as technical triplicates (mean ± SD). (Sensor 15) no inhibitor. no viral neuraminidase control (4) Thymol.

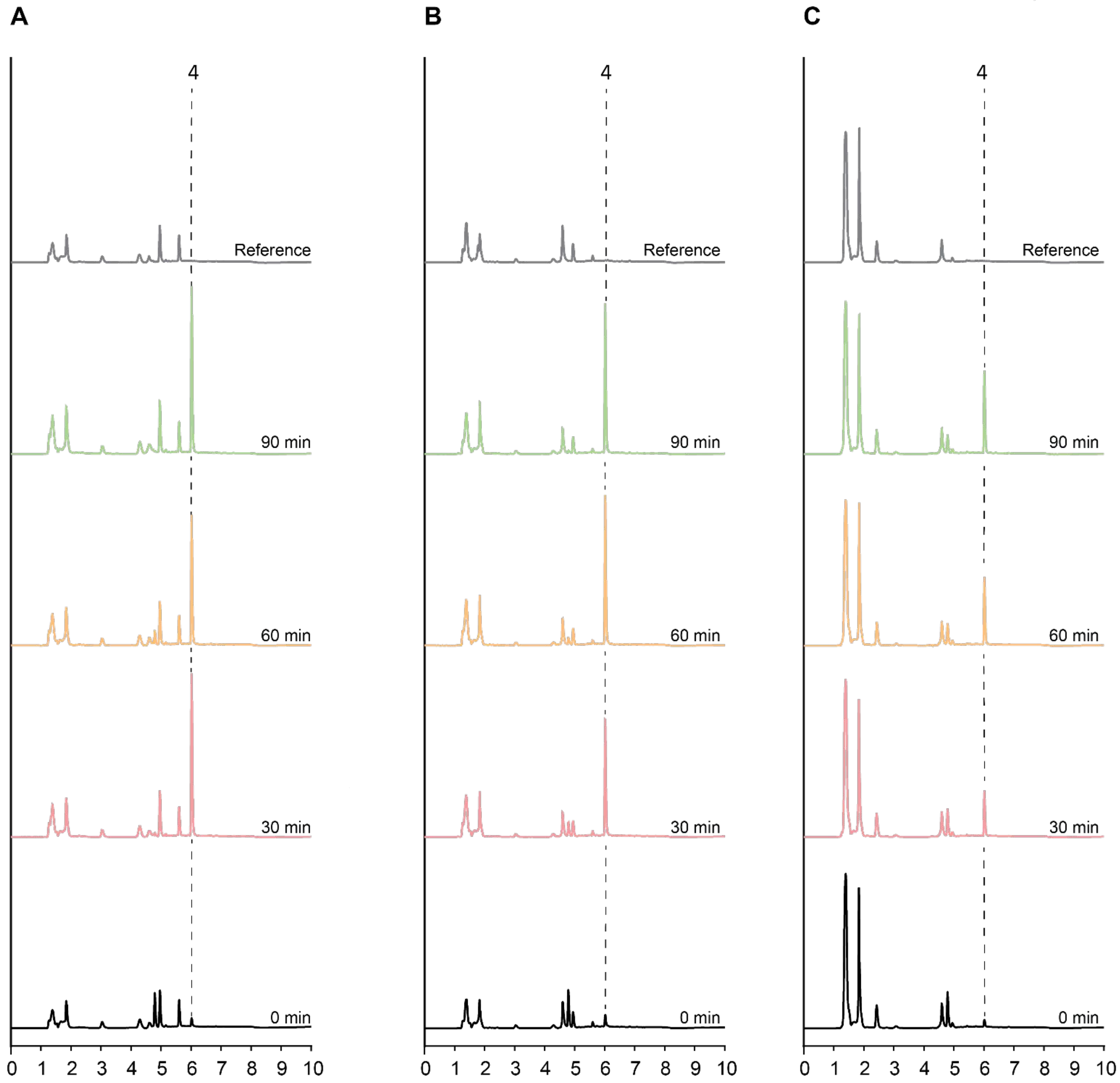

Figure S13. Unmethylated reference sensor (**6**) stability in saliva of healthy people. Measurements were performed as biological triplicate. (4) Thymol.

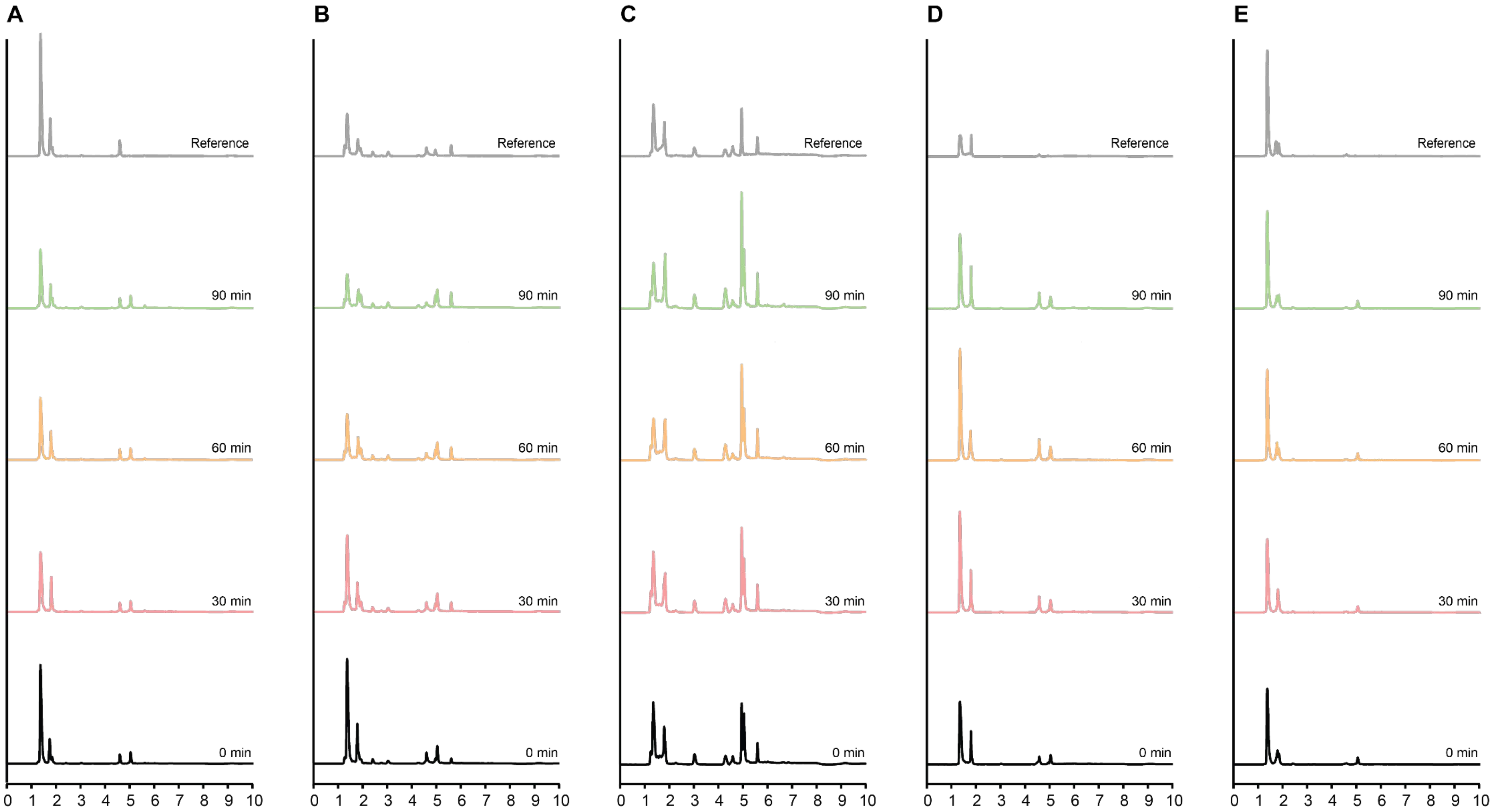

Figure S14. α-Sensor (**15**) stability in saliva of healthy people (day 1). Measurements were performed as five biological replicates (n=5).

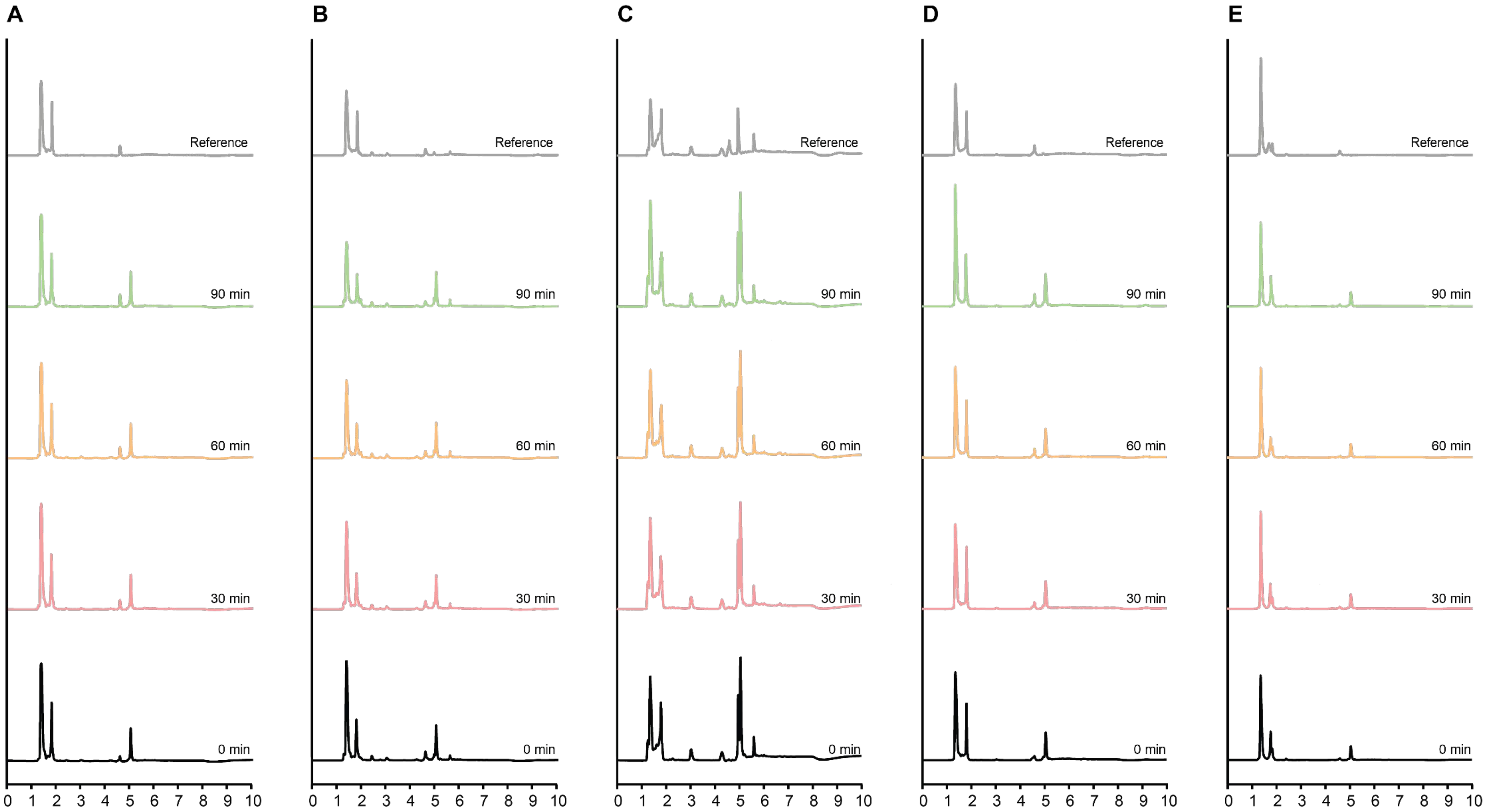

Figure S15: α-Sensor (**15**) incubated in saliva of healthy people (day 1) spiked with bacterial neuraminidase. Measurements were performed in five biological replicates (n=5).

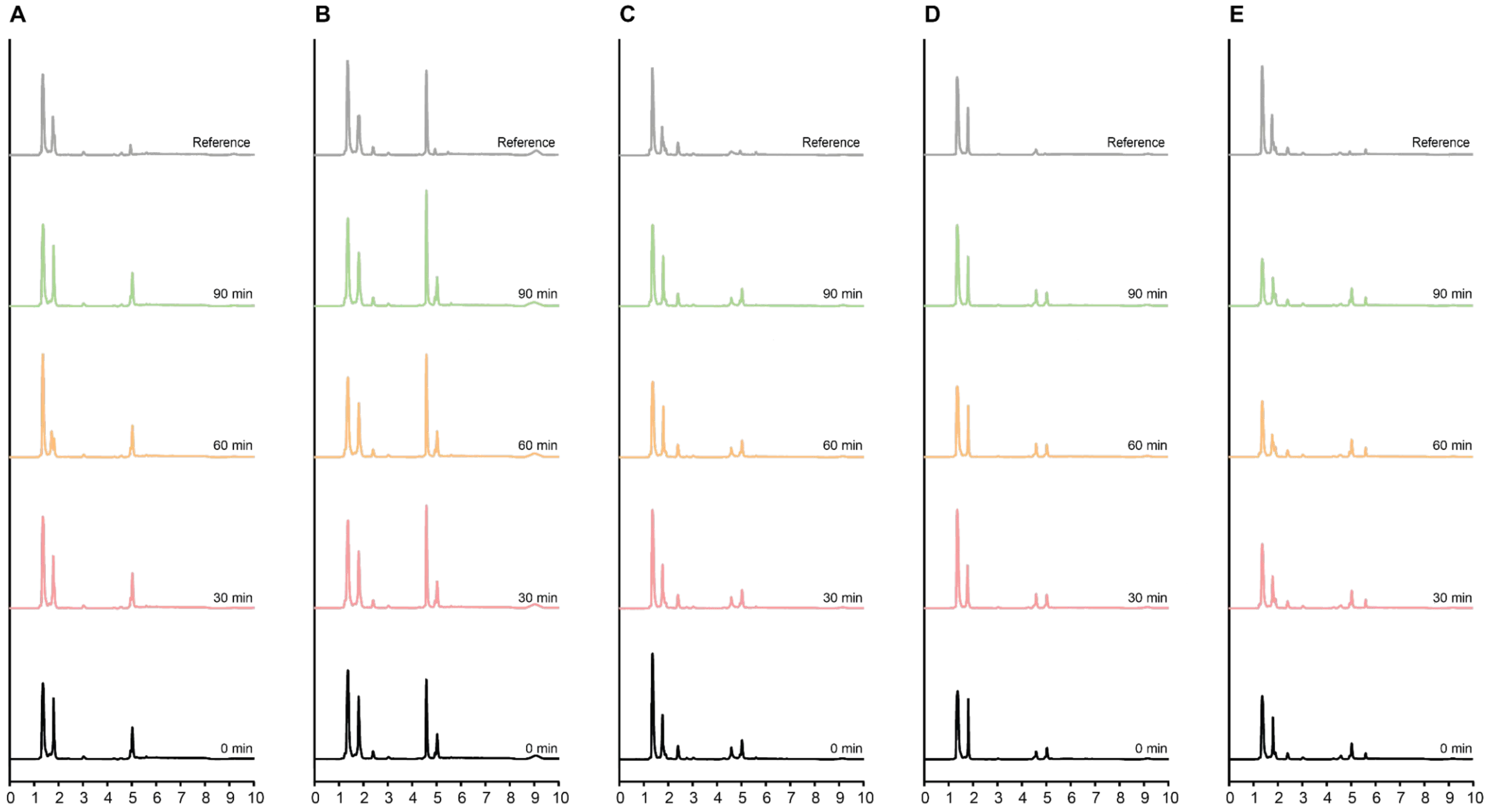

Figure S16. α-Sensor (**15**) stability in saliva of healthy people (day 2). Measurements were performed as five biological replicates (n=5).

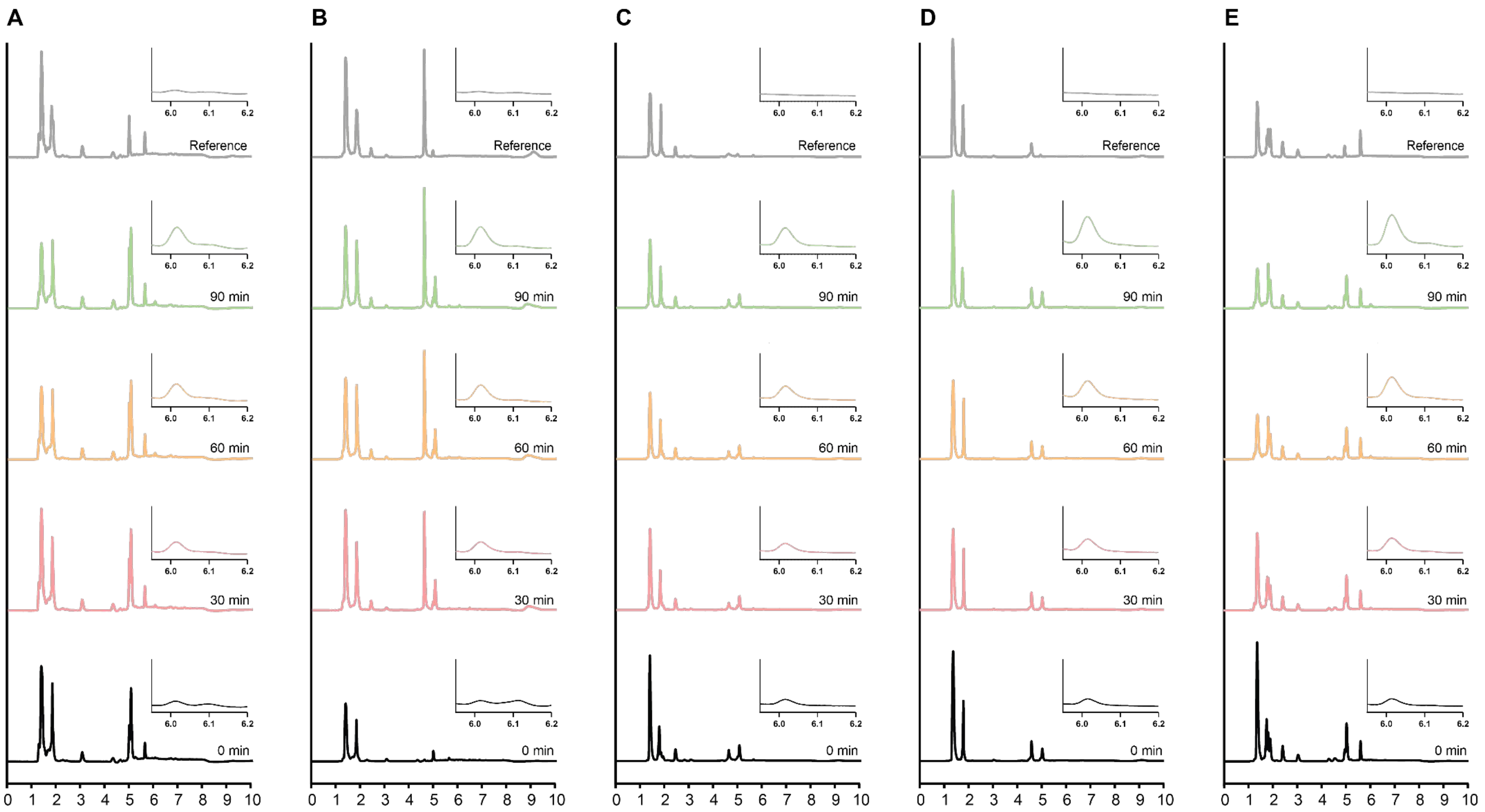

Figure S17. α-Sensor (**15**) incubated in saliva of healthy people (day 2) spiked with viral neuraminidase. Measurements were performed as five biological replicates (n=5). In panel (B), no sugar can be detected at 0 min; this measurement was therefore not included in the calculations (**Figure 3**).

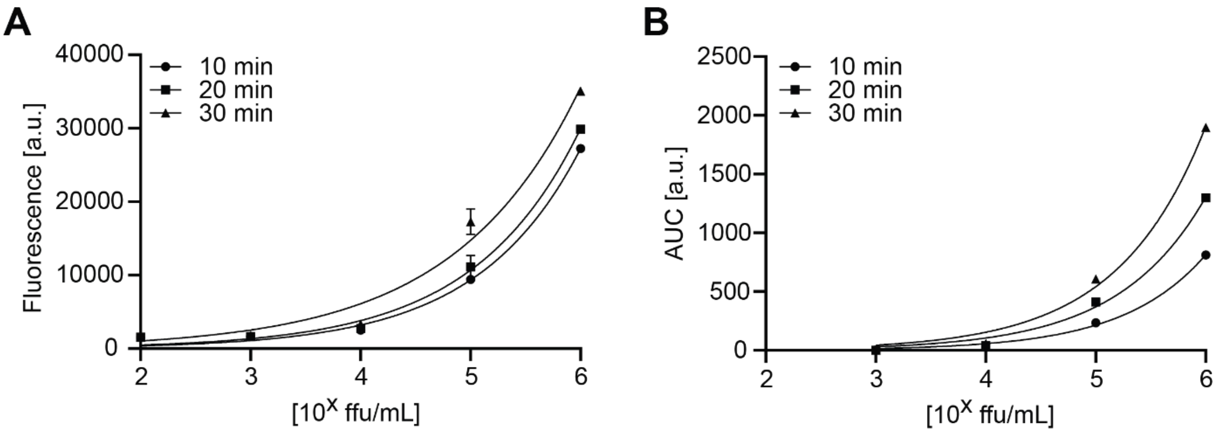

Figure S18: Release of (A) 4-methylumbelliferone from 4-MUNANA and (B) thymol from α-sensor (**15**) in response to different concentrations of H1N1 (A/California/7/2009) ffu/mL and assessed after 10, 20, and 30 min, respectively. All measurements were performed as technical triplicates (mean ± SD). The 4MU standard curve had an R^2 = 0.99.

Comparison with ZstatFlu-II®

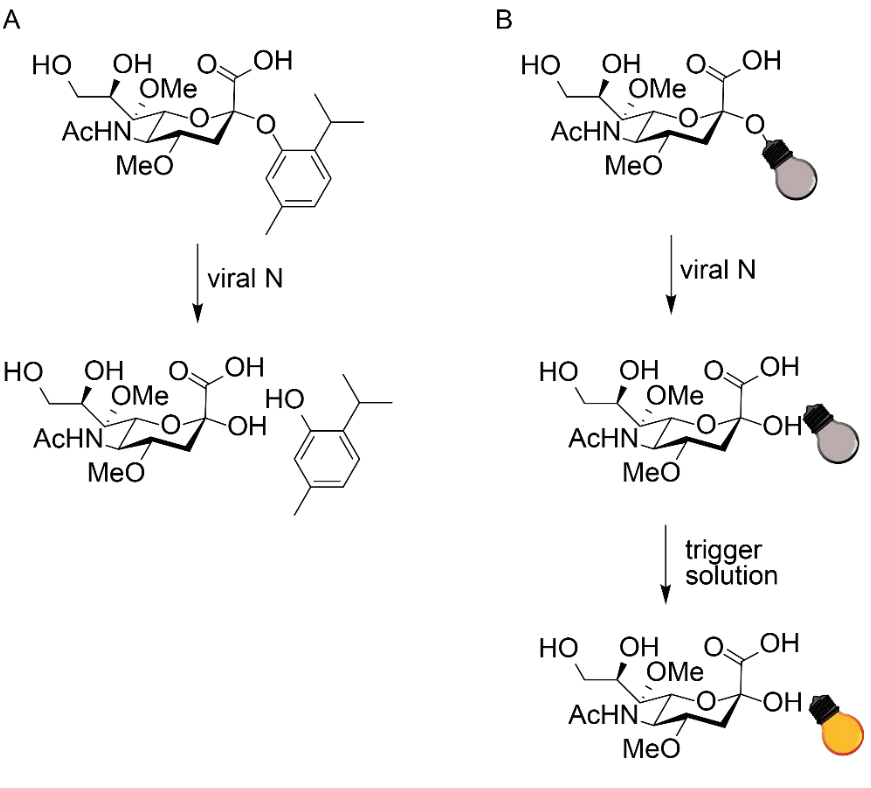

Figure S19. Comparison of (A) a taste-based test system with (B) the test system ZstatFlu-II®.[31]

Crystal Structures of Bacterial and Mammalian Neuraminidases

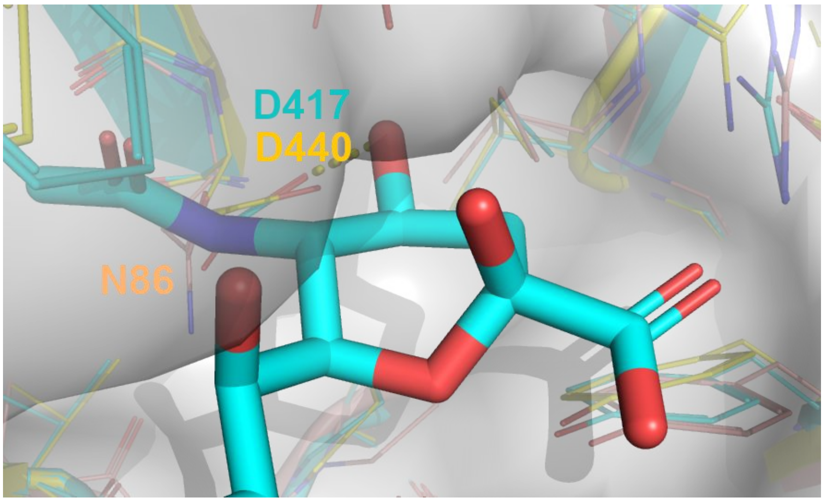

Figure S20. Comparison of the binding pockets of *Streptococcus pneumonia*, *Streptococcus oralis*, and human neuraminidases. As reference, the crystal structure of neuraminidase NanA of *Streptococcus pneumonia* (PDB:3H72[32]) is shown in cyan, with *N*-acetylneuraminic acid as ligand. Additional structures of *Staphylococcus oralis* neuraminidase NanA (yellow, obtained from AlphaFold[33], AF-A0A1L7HA05-F1-v4) and human cytosolic neuraminidase Neu2 (orange/pink, PDB: 1SNT[34]) are superposed. For all cases, the binding pocket more closely resembles that of *M. viridifaciens*, with the O4 hydroxyl group in close proximity to an aspartate or asparagine as opposed to the extended pocket available in viral H1N1. Upon insertion of a methoxy group in this position, steric clashes and a loss in hydrogen bonding could contribute to a reduced affinity.

Crystal Structure of Neuraminic Acid

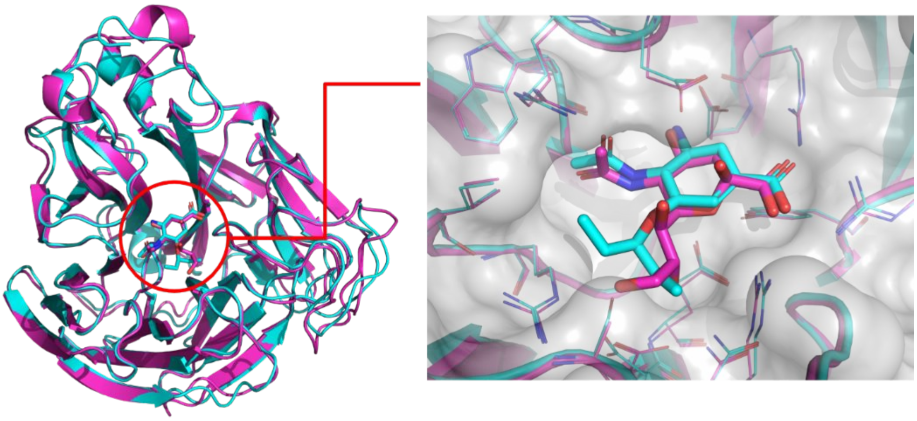

Figure S21. Superposition of the influenza H1N1 complexed with 2-deoxy-2,3-dehydro-*N*-acetylneuraminic acid (cyan, PDB: 1EUS[35]) and H3N2 complexed with neuraminic acid (pink, PDB: 8DWB[36]), showing that neuraminic acid establishes a similar binding mode to dehydrated inhibitors via formation of a distorted pyranose conformation.

Molecular Docking Results

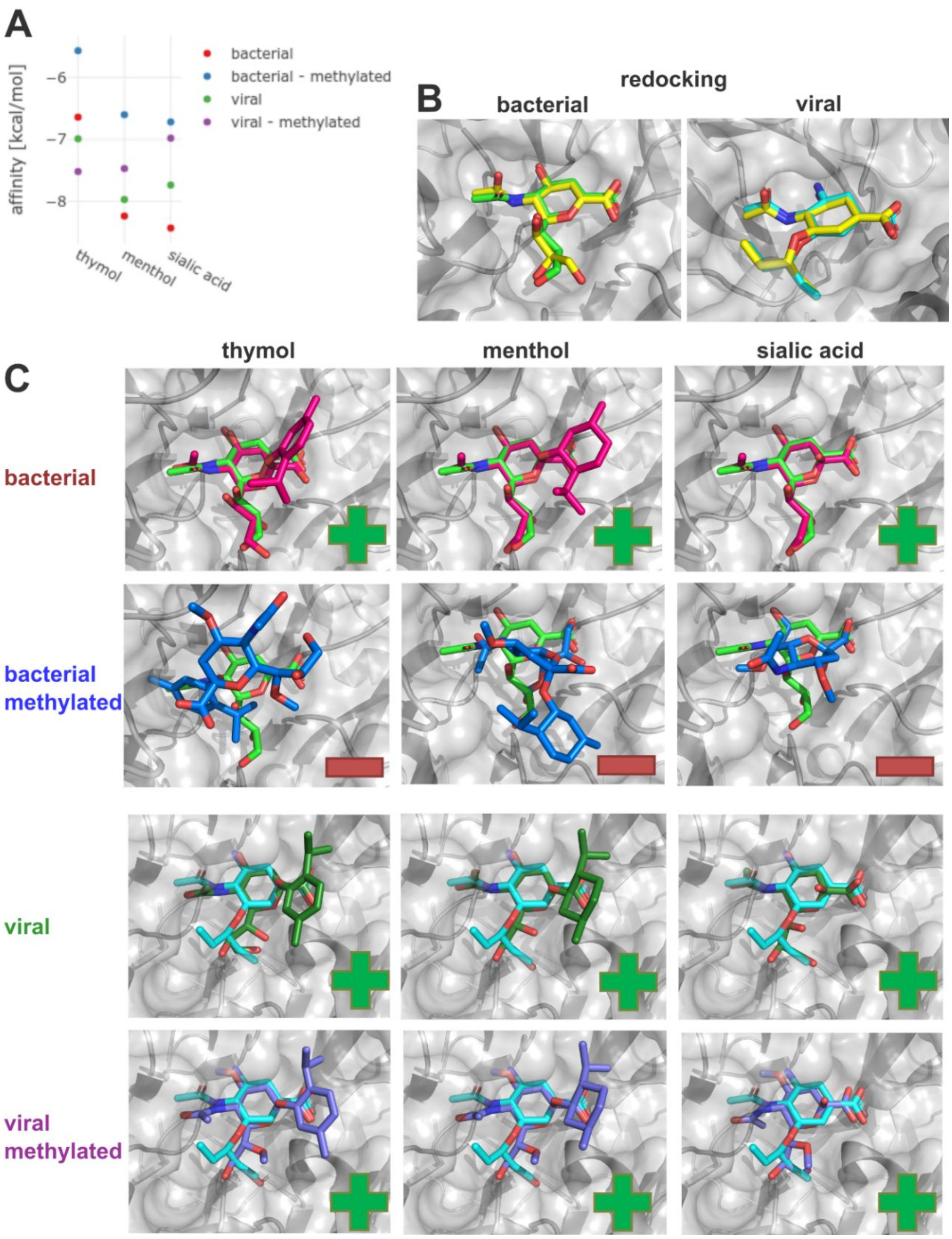

Figure S22. Molecular docking results. (A) CNN affinity scores for the best binding poses of each docking run. The scores are grouped by ligand type and colored according to methylation and receptor type. Methylated sensors show the most positive (unfavorable) affinity score across all bacterial systems as none of the top 10 binding poses showed the respective ligand to enter the active site. (B) Redocking poses of native ligands oseltamivir and 2-deoxy-2,3-dehydro-*N*-acetyl neuraminic acid, colored yellow and shown together with the crystal binding pose. (C) Top poses for each system shown in corresponding colors to the legend in (A). Symbols (+) and (-) indicate whether the pyranose ring establishes a similar binding pose to the native ligand.

Characterization of Compounds

A

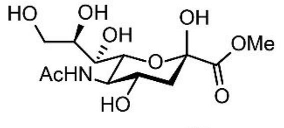

B

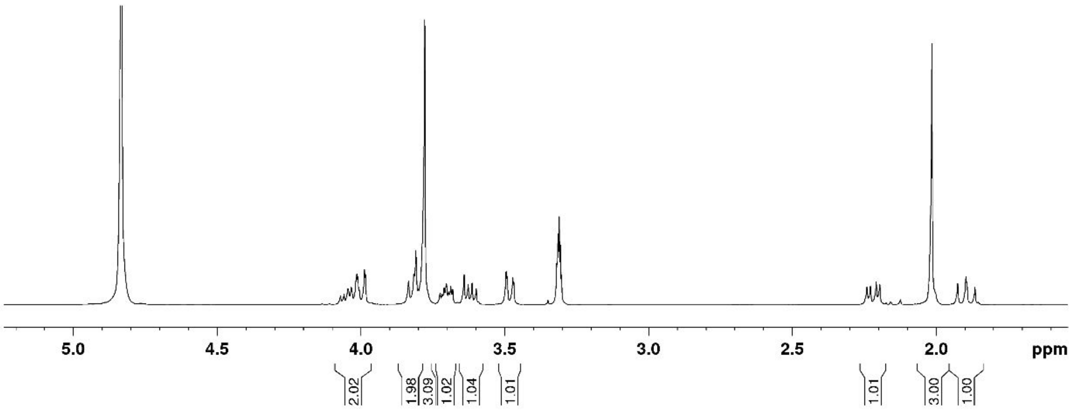

C

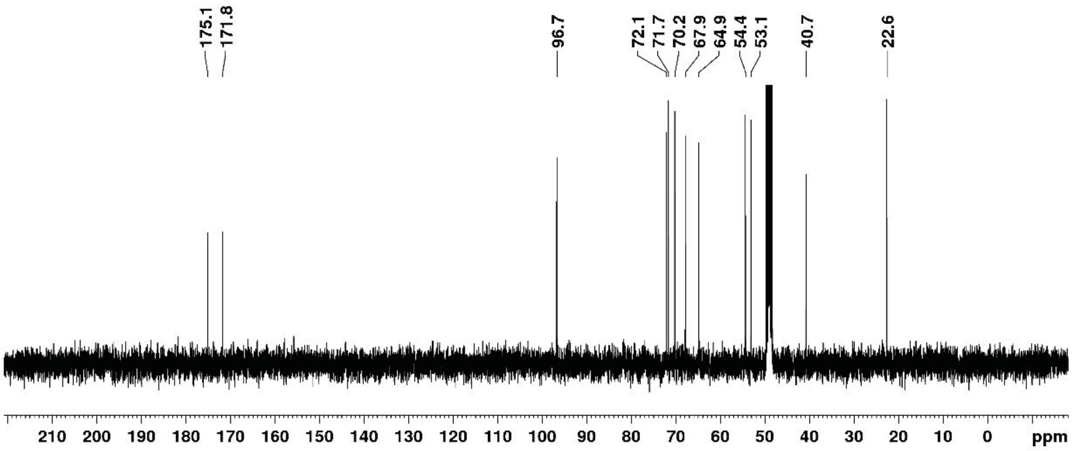

Figure S23. (A) compound (**2**) (B) ^{1}H NMR (400 MHz) spectrum of compound (**2**) in CD_3OD (C) ^{13}C NMR spectrum of compound (**2**) in CD_3OD.

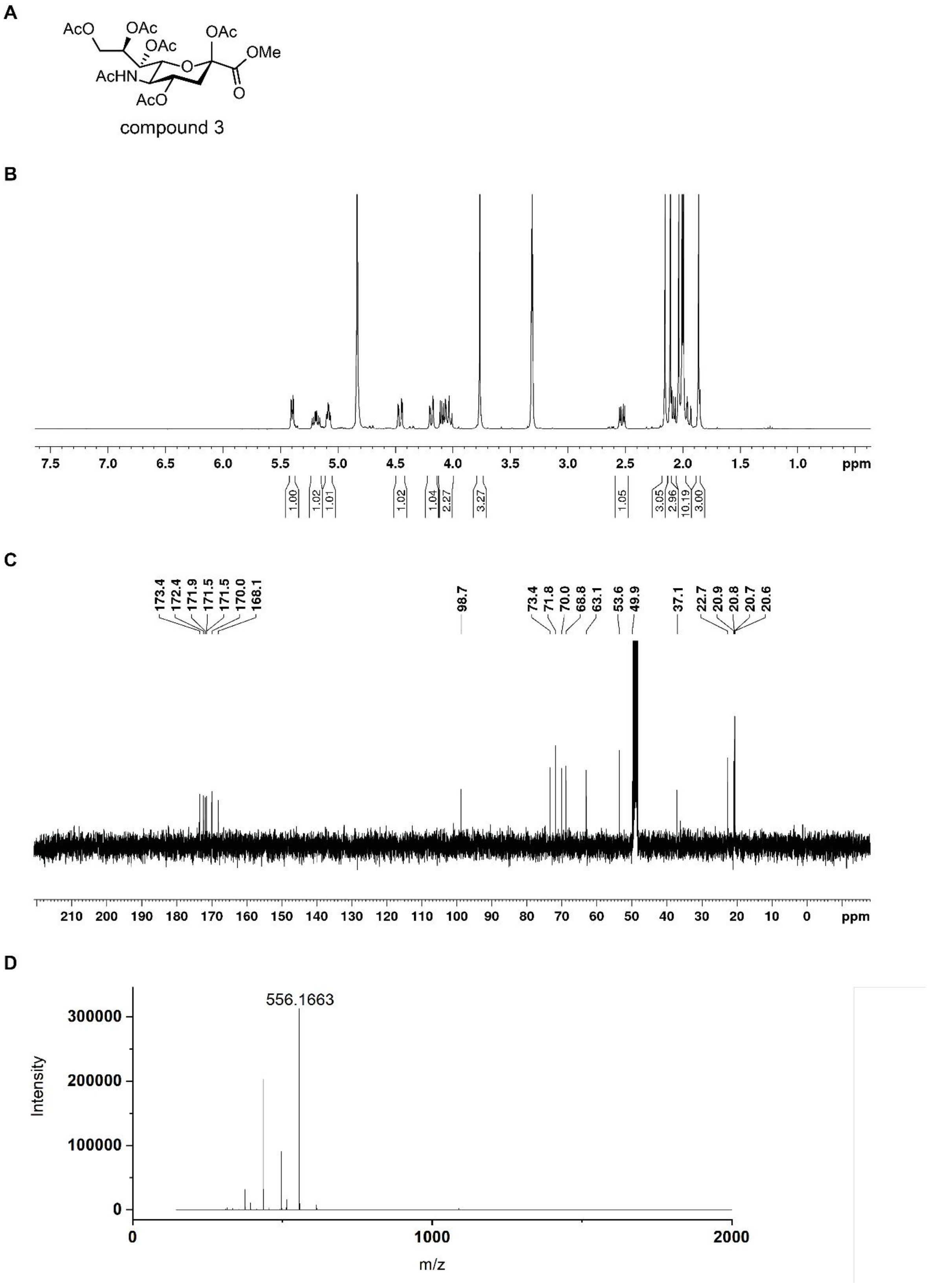

Figure S24. (A) compound (**3**) (B) ^{1}H NMR (400 MHz) spectrum of compound (**3**) in CD_3OD (C) ^{13}C NMR spectrum of compound (**3**) in CD_3OD (D) HRMS (ESI+) of compound (**3**) calculation for $[C_{22}H_{31}NNaO_{14}]^+$ 556.16368; found 556.16637.

A

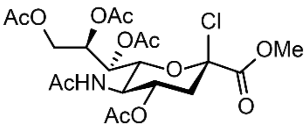

compound 4-1

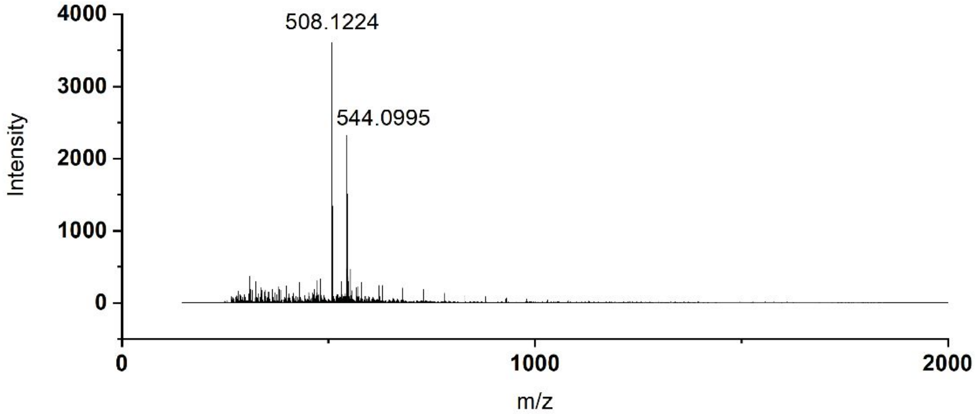

Figure S25. (A) compound (**4-1**) (B) HRMS (ESI-) of compound (**4-1**) calculation for $[C_{20}H_{27}ClNO_{12}]^-$ 508.1227; found 508.1224 and calculation for $[C_{20}H_{28}Cl_2NO_{12}]^-$ 544.0994; found 544.0995.

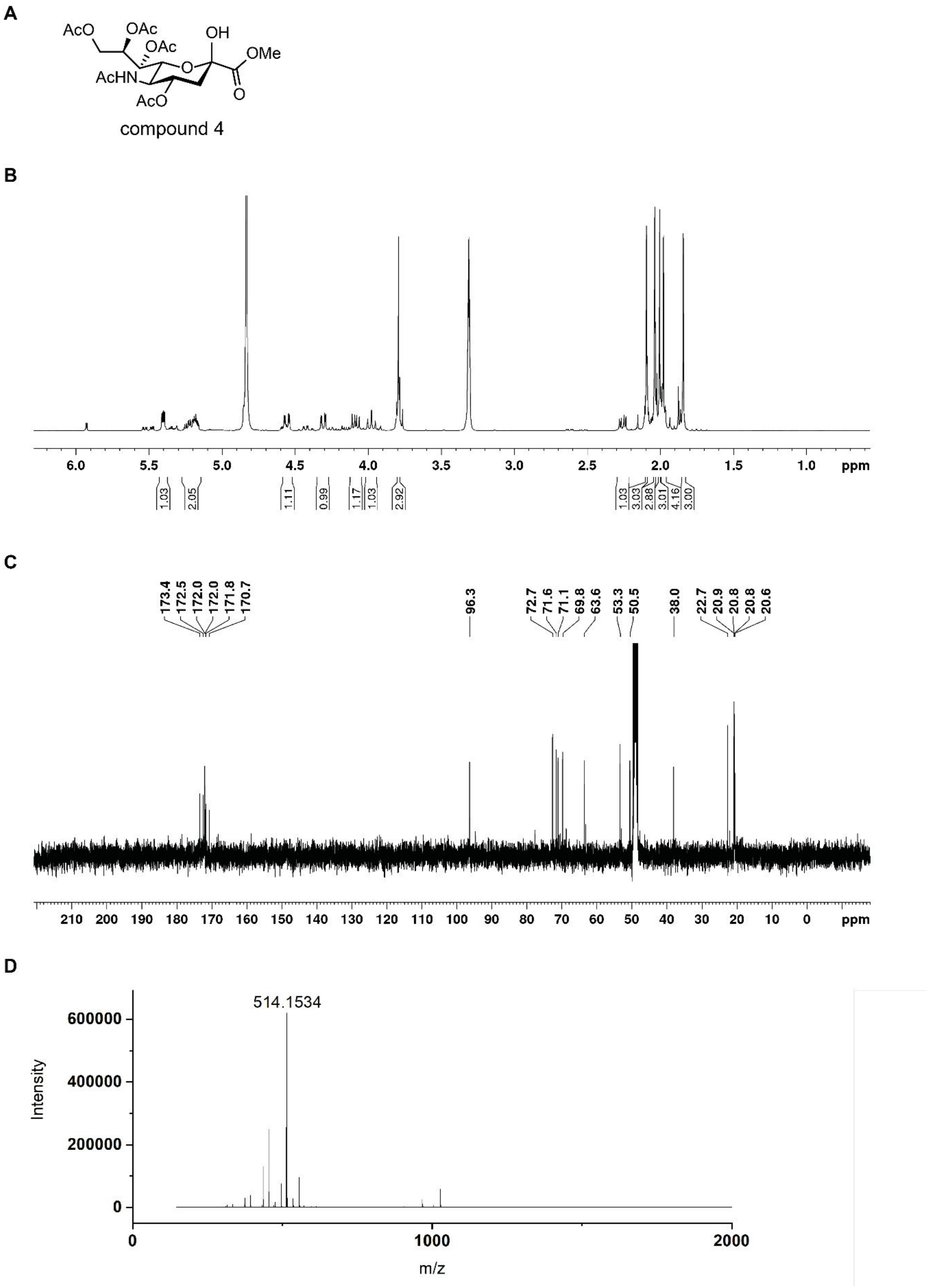

Figure S26. (A) Compound (**4**) (B) ^{1}H NMR (400 MHz) spectrum of compound (**4**) in CD_3OD (C) ^{13}C NMR spectrum of compound (**4**) in CD_3OD (D) HRMS (ESI+) of compound (**4**) calculation for $[C_{20}H_{29}NNaO_{13}]^+$ 514.1531; found 514.1534.

A

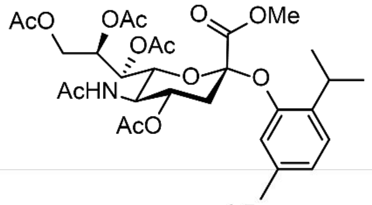

compound 5

B

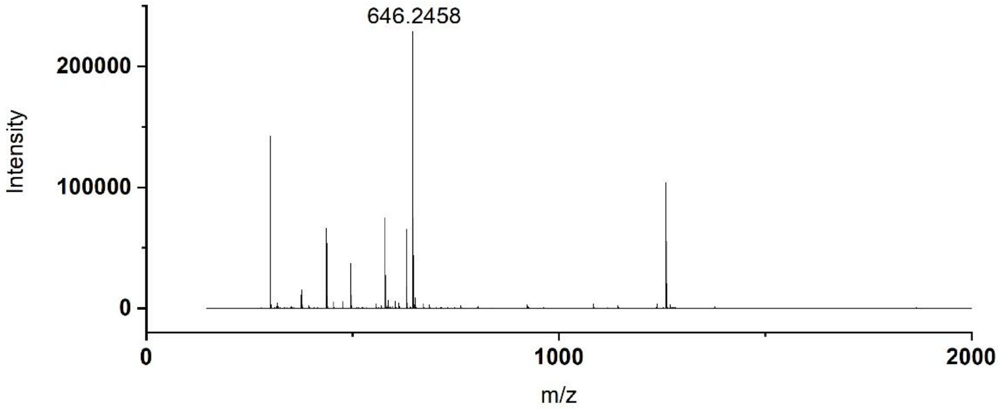

Figure S27. (A) Compound (**5**) (B) HRMS (ESI+) of compound (**5**) calculation for $[C_{30}H_{41}NNaO_{13}]^+$ 646.24701; found 646.24587.

A

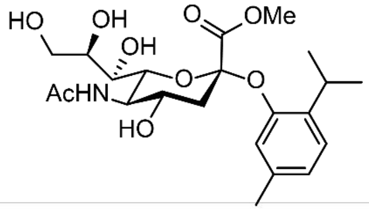

compound 6-1

B

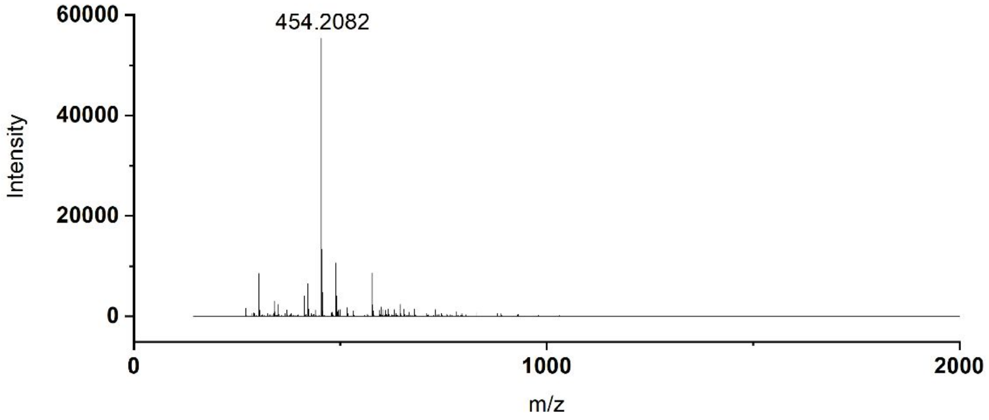

Figure S28. (A) Compound (**6-1**) (B) HRMS (ESI-) of compound (**6-1**) calculation for $[C_{22}H_{32}NO_9]^-$ 454.20826; found 454.20820.

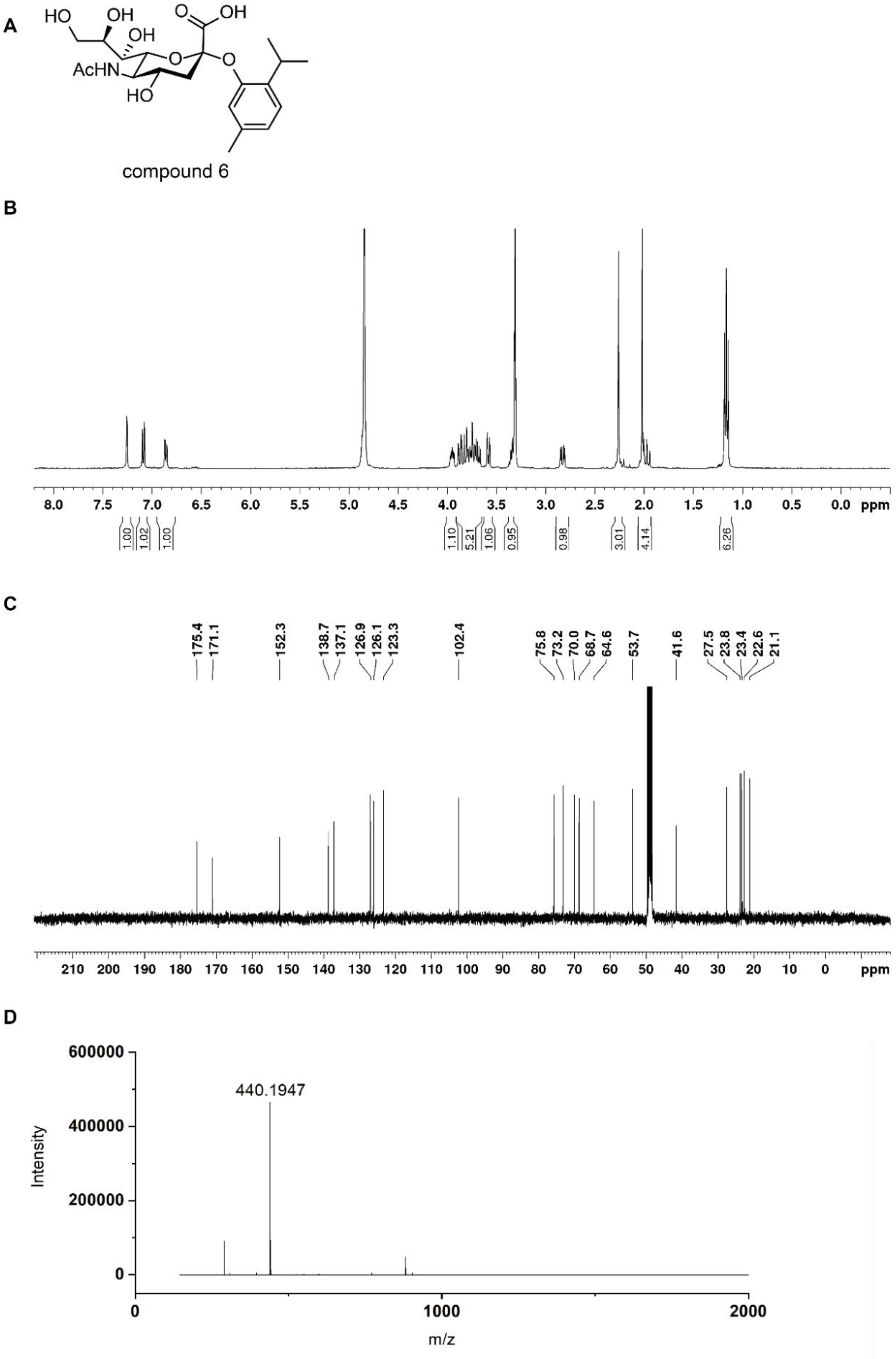

Figure S29. (A) Compound (**6**) (B) ^{1}H NMR (400 MHz) spectrum of compound (**6**) in CD_3OD (C) ^{13}C NMR spectrum of compound (**6**) in CD_3OD (D) HRMS (ESI-) of compound (**6**) calculation for $[C_{21}H_{30}NO_9]^-$ 440.19261; found 440.19474.

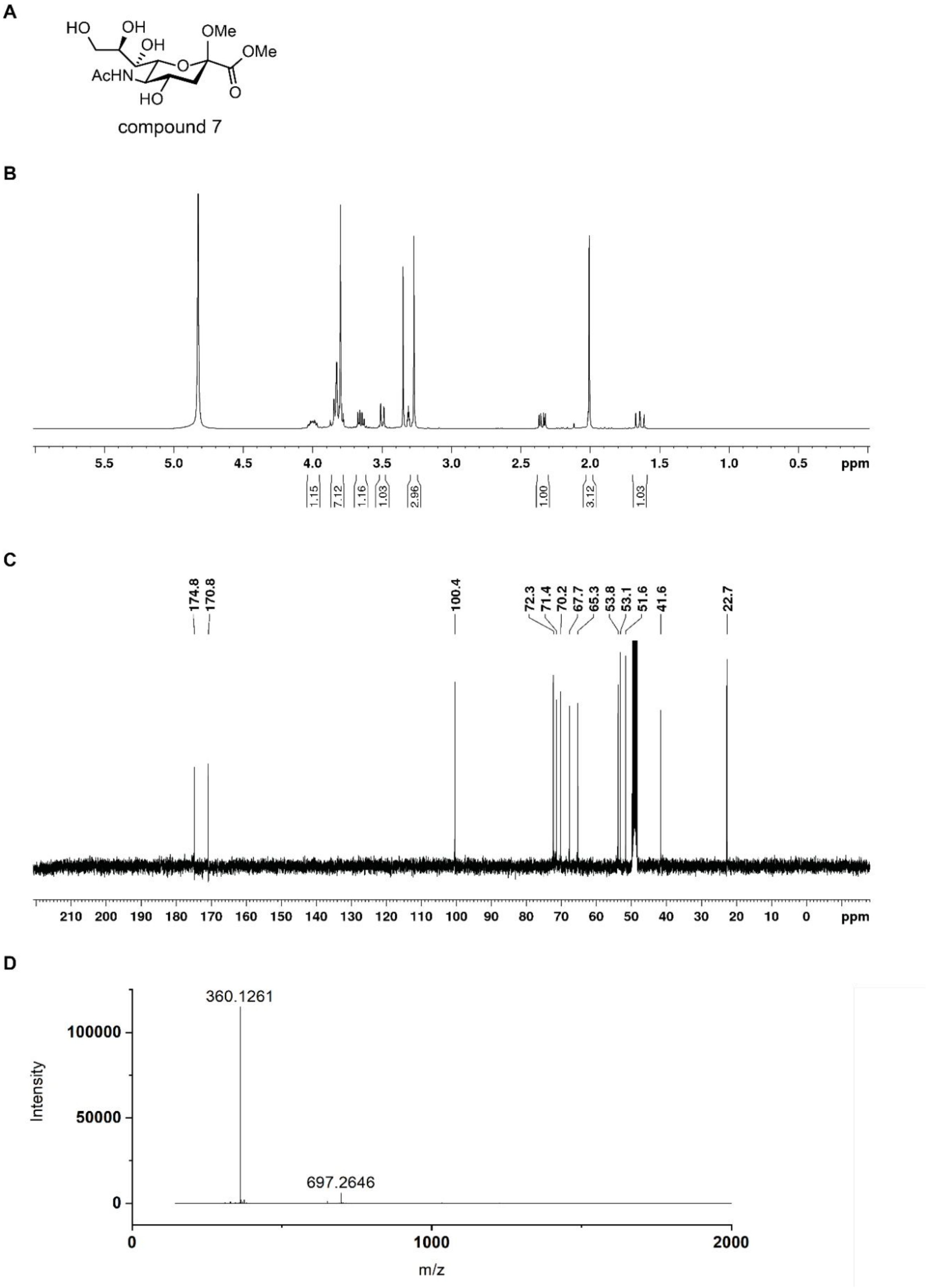

Figure S30. (A) Compound (**7**) (B) ^{1}H NMR (400 MHz) spectrum of compound (**7**) in CD_3OD (conventional synthesis) (C) ^{13}C NMR spectrum of compound (**7**) in CD_3OD (conventional synthesis) (D) HRMS (ESI+) of compound (**7**) calculation for $[C_{13}H_{23}NNaO_9]^+$ 360.12650; found 360.12612 and calculation for $[C_{26}H_{46}N_2NaO_{18}]^+$ 697.26378; found 697.26468 (conventional synthesis).

A

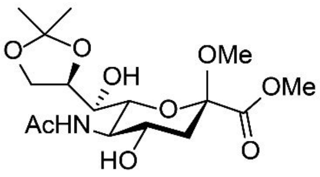

compound 8

B

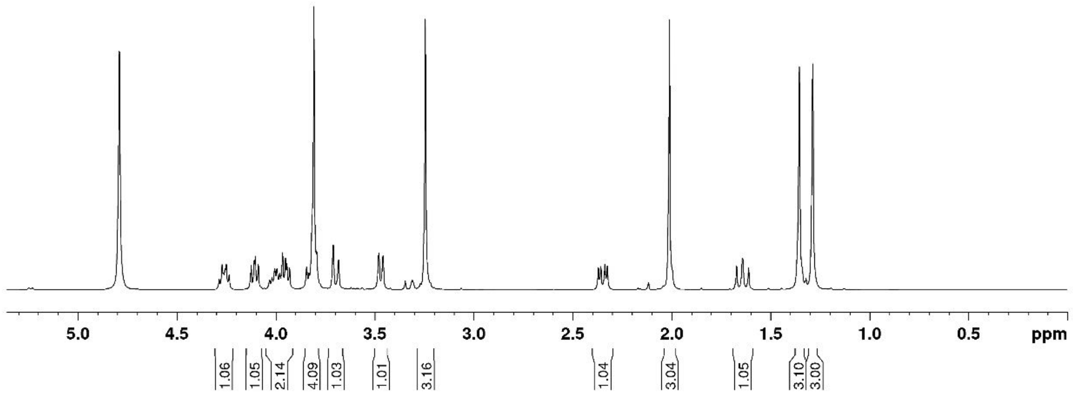

C

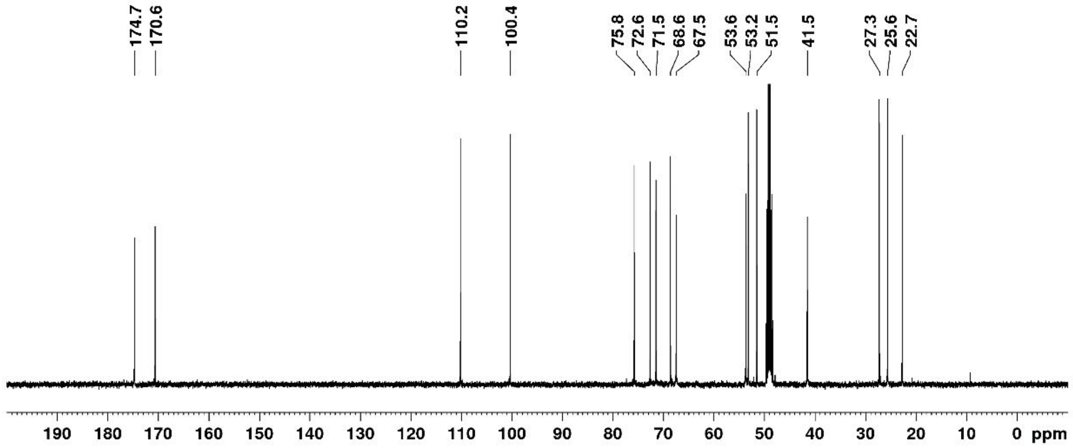

D

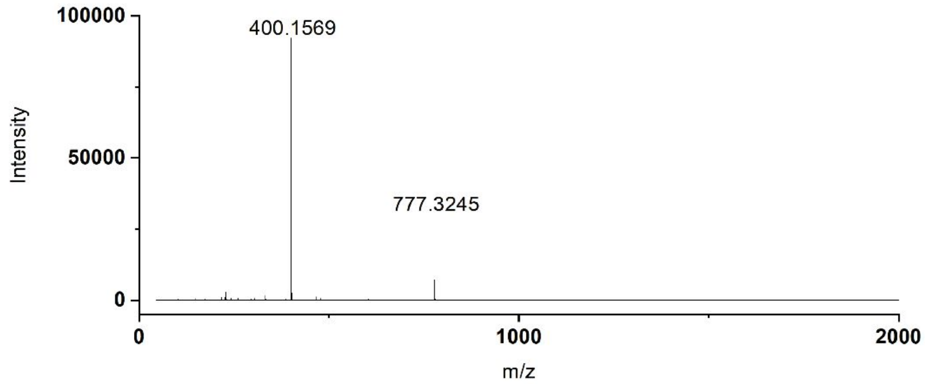

Figure S31. (A) Compound (**8**) (B) ^{1}H NMR (400 MHz) spectrum of compound (**8**) in CD_3OD (C) ^{13}C NMR spectrum of compound (**8**) in CD_3OD (D) HRMS (ESI+) of compound (**8**) calculation for $[C_{16}H_{27}NNaO_9]^+$ 400.15780; found 400.15692 and calculation for $[C_{32}H_{54}N_2NaO_{18}]^+$ 777.32638; found 777.32452.

A

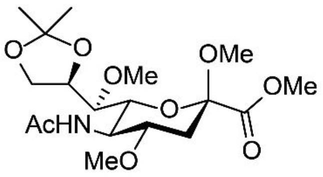

compound 9

B

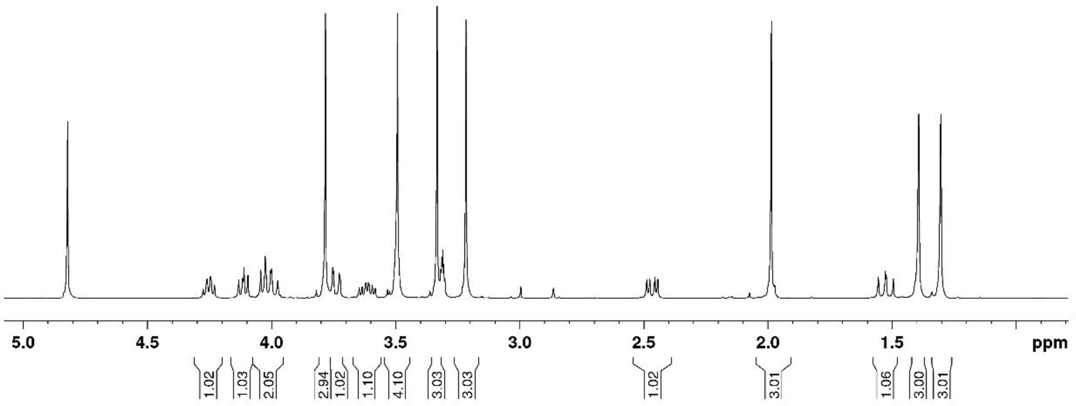

C

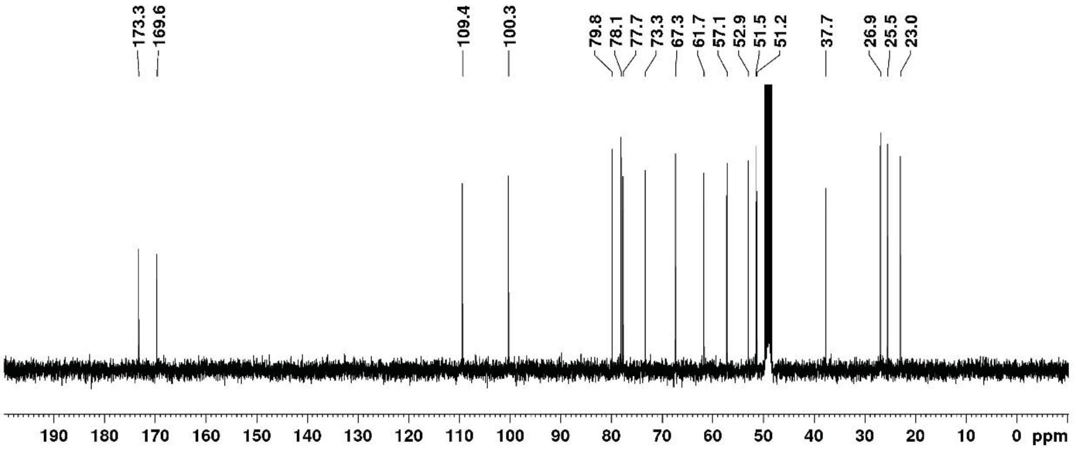

D

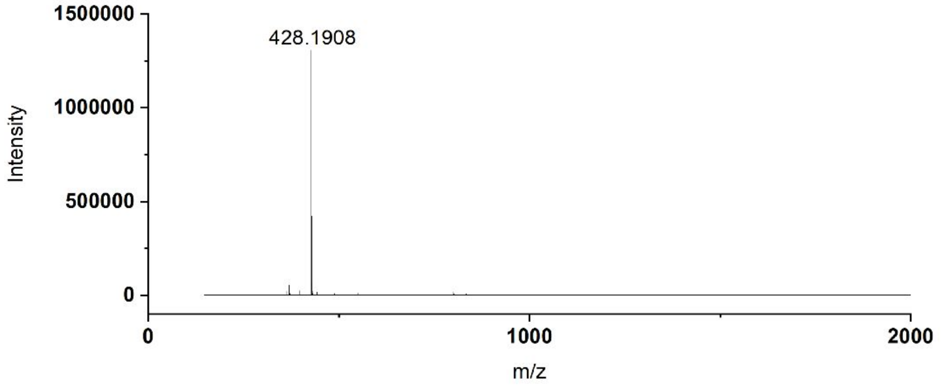

Figure S32. (A) Compound (**9**) (B) ^{1}H NMR (400 MHz) spectrum of compound (**9**) in CD_3OD (C) ^{13}C NMR spectrum of compound (**9**) in CD_3OD (D) HRMS (ESI+) of compound (**9**) calculation for $[C_{18}H_{31}NNaO_9]^+$ 428.1891; found 428.1908.

A

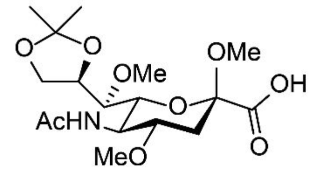

compound 10-1

B

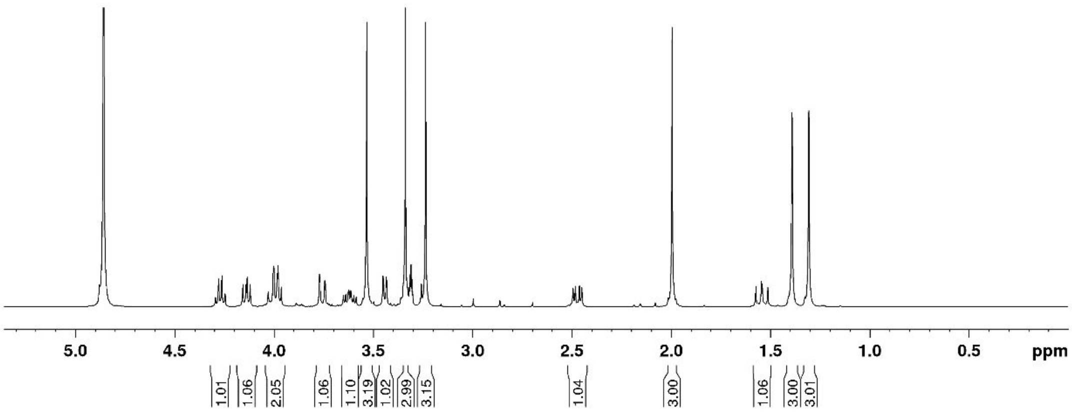

C

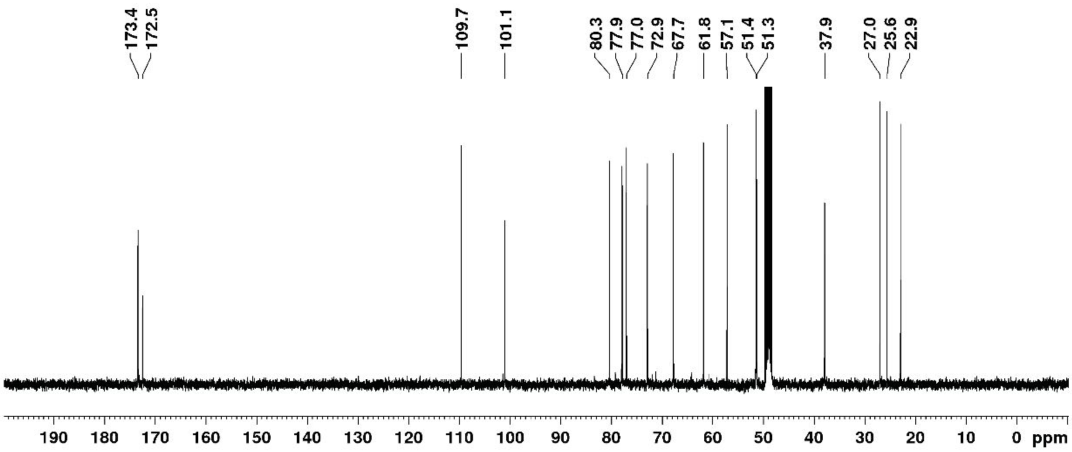

D

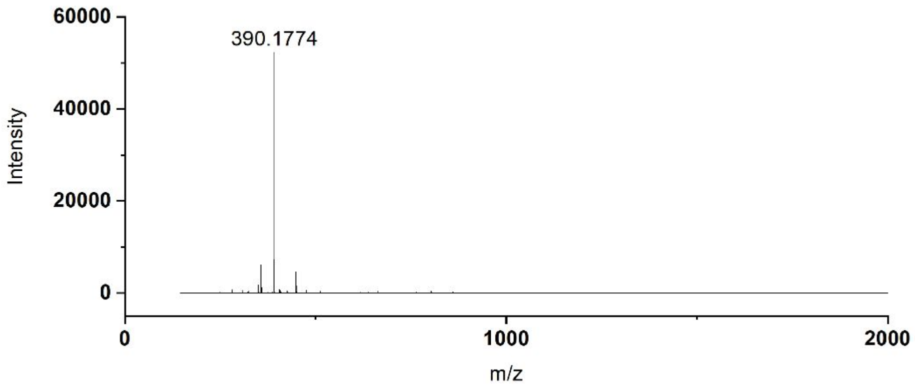

Figure S33. (A) Compound (**10-1**) (B) ^{1}H NMR (400 MHz) spectrum of compound (**10-1**) in CD_3OD (C) ^{13}C NMR spectrum of compound (**10-1**) in CD_3OD (D) HRMS (ESI-) of compound (**10-1**) calculation for $[C_{17}H_{28}NO_9]^-$ 390.17696; found 390.17743.

A

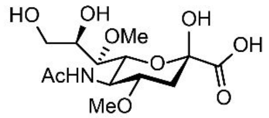

compound 10

B

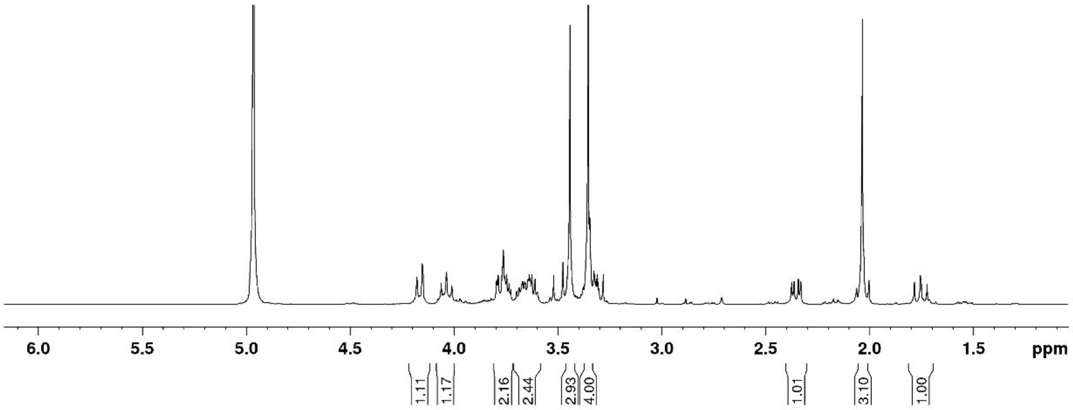

C

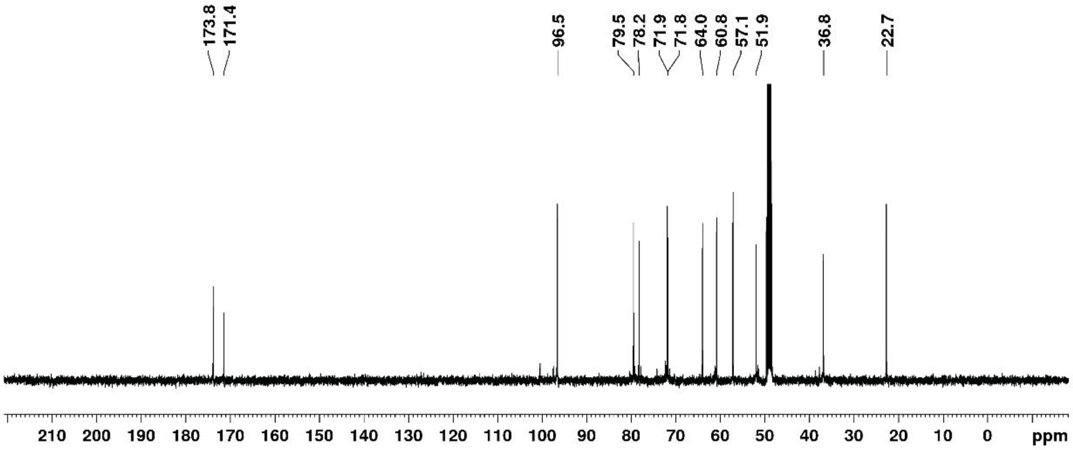

D

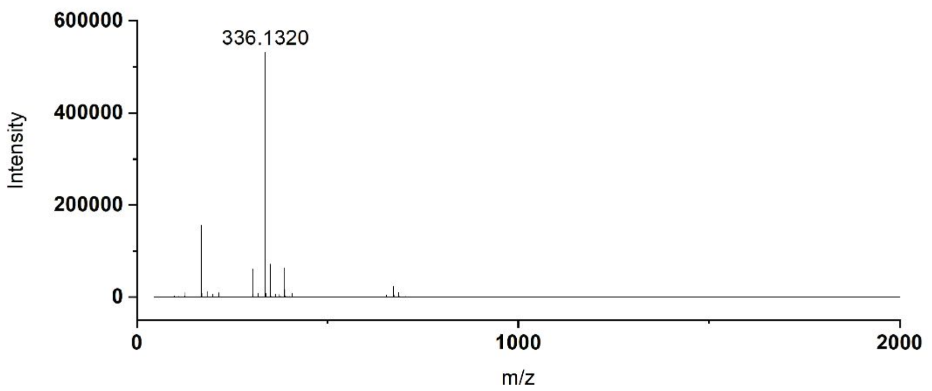

Figure S34. (A) Compound (**10**) (B) ^{1}H NMR (400 MHz) spectrum of compound (**10**) in CD_3OD (C) ^{13}C NMR spectrum of compound (**10**) in CD_3OD (D) HRMS (ESI-) of compound (**10**) calculation for $[C_{13}H_{22}NO_9]^-$ 336.12945; found 336.13204.

A

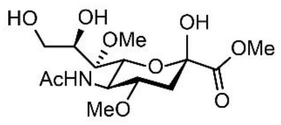

compound 11

B

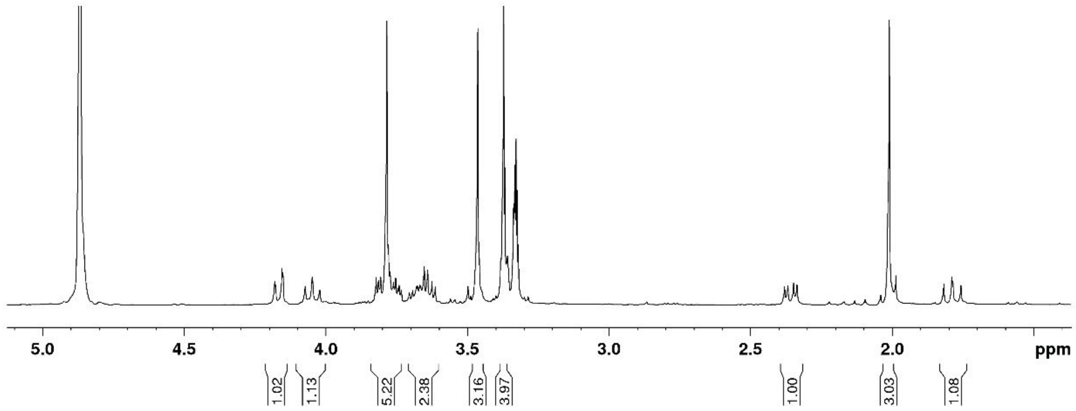

C

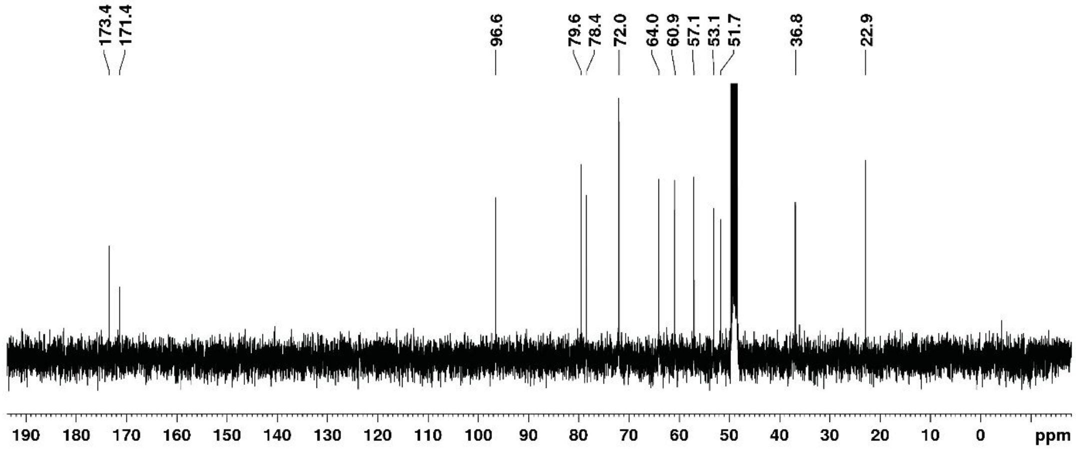

Figure S35. (A) Compound (**11**) (B) ^{1}H NMR (400 MHz) spectrum of compound (**11**) in CD_3OD (C) ^{13}C NMR spectrum of compound (**11**) in CD_3OD.

A

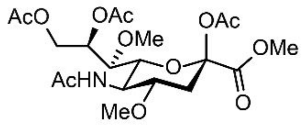

compound 12

B

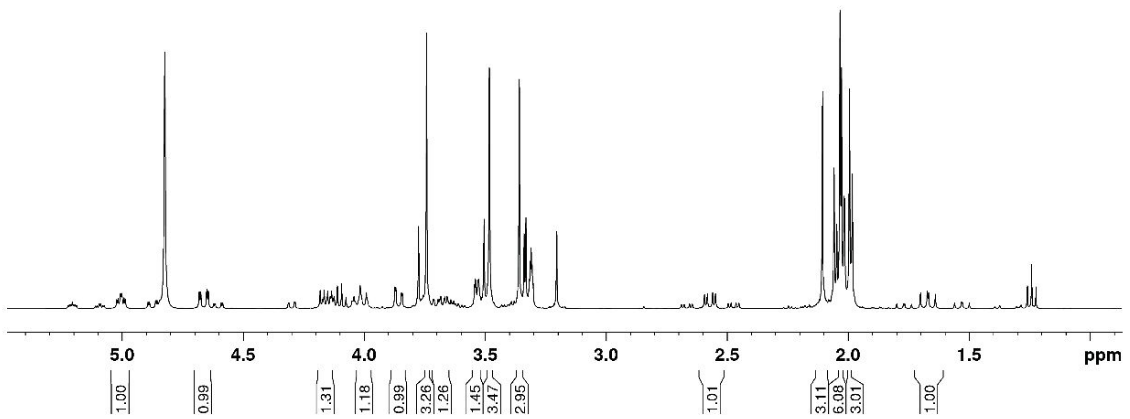

C

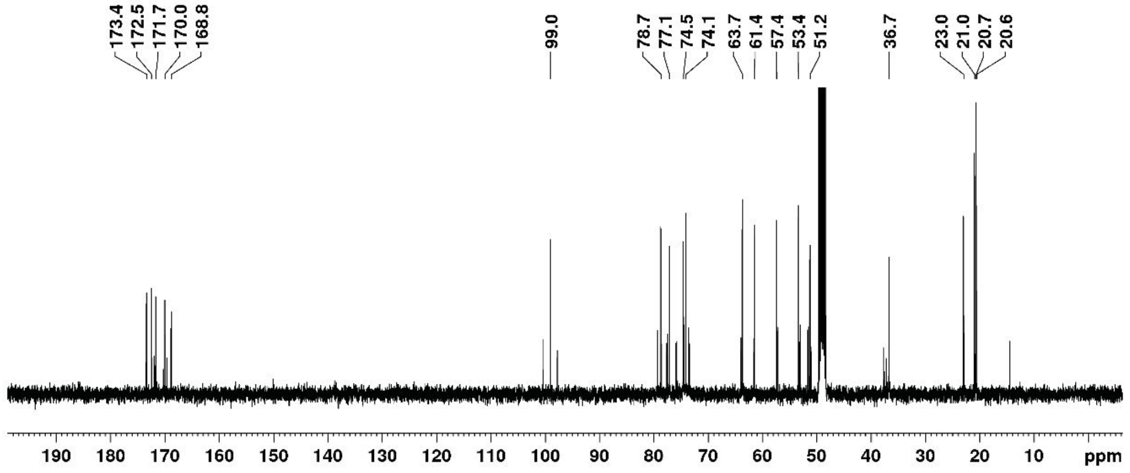

D

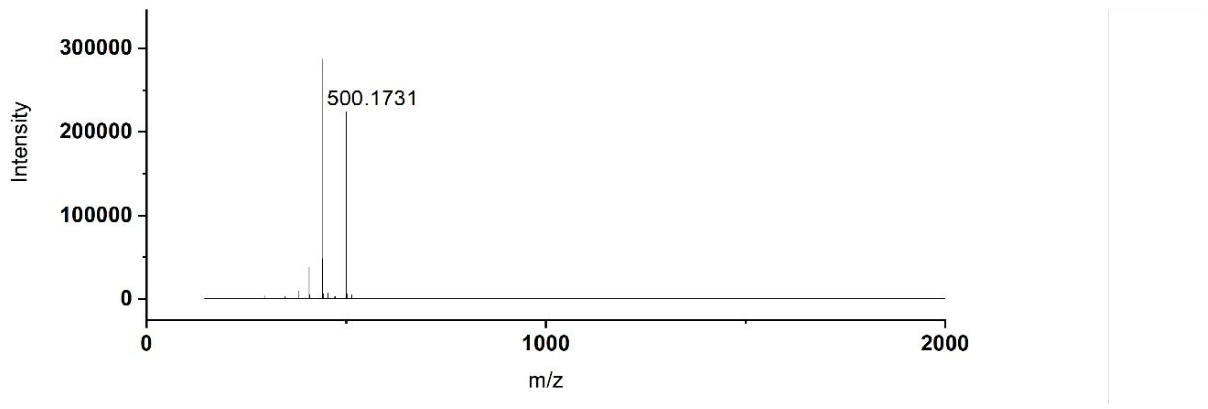

Figure S36. (A) Compound (**12**) (B) ^{1}H NMR (400 MHz) spectrum of compound (**12**) in CD_3OD (C) ^{13}C NMR spectrum of compound (**12**) in CD_3OD (D) HRMS (ESI+) of compound (**12**) calculation for $[C_{20}H_{31}NNaO_{12}]^+$ 500.17385; found 500.17313.

A

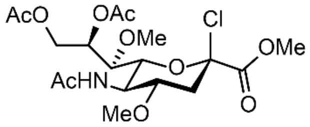

compound 13-1

B

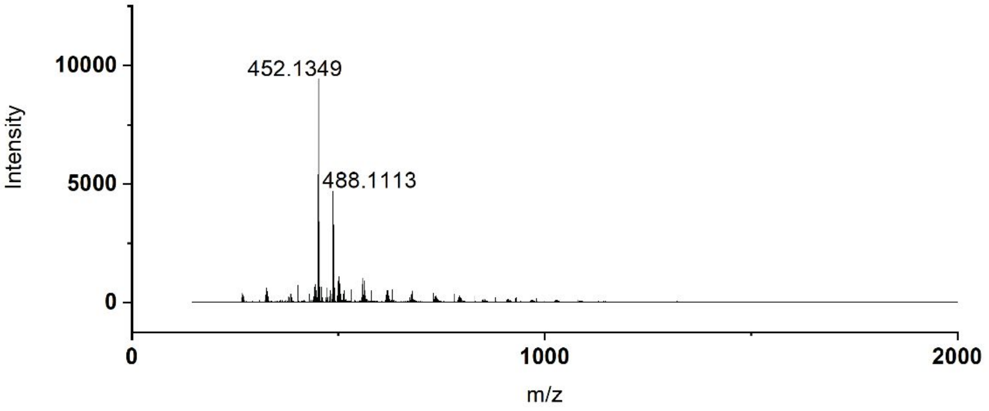

Figure S37. (A) Compound (**13-1**) (B) HRMS (ESI-) of compound (**13-1**) calculation for $[C_{18}H_{27}ClNO_{10}]^-$ 452.1329; found 452.1349 and calculation for $[C_{18}H_{28}Cl_2NO_{10}]^-$ 488.1096; found 488.1113.

A

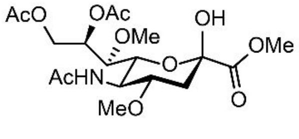

compound 13

B

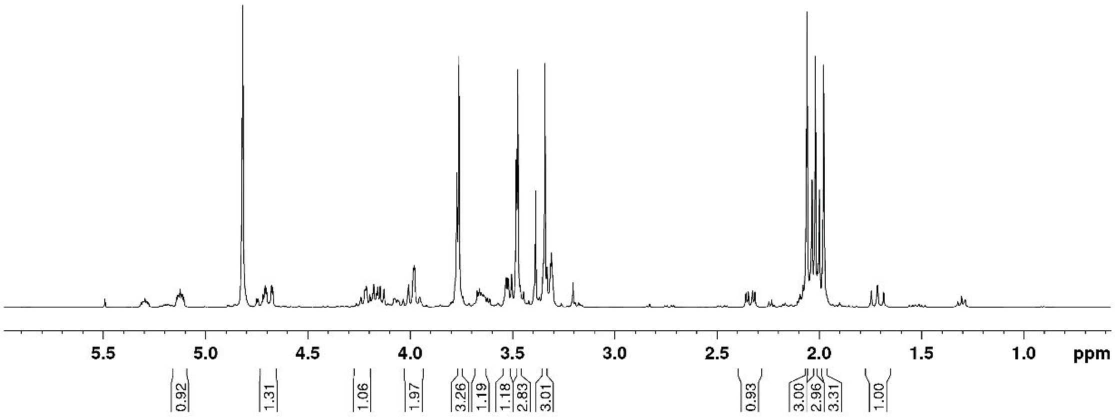

C

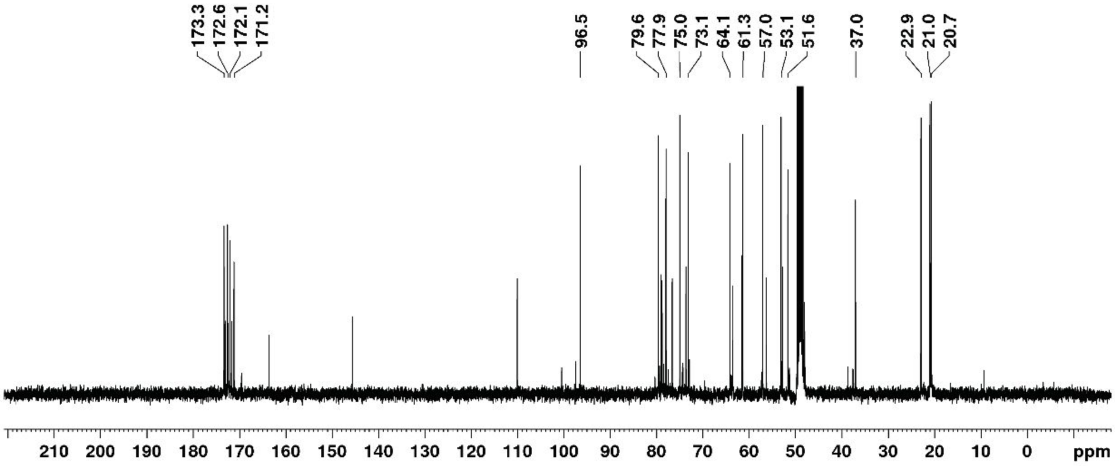

D

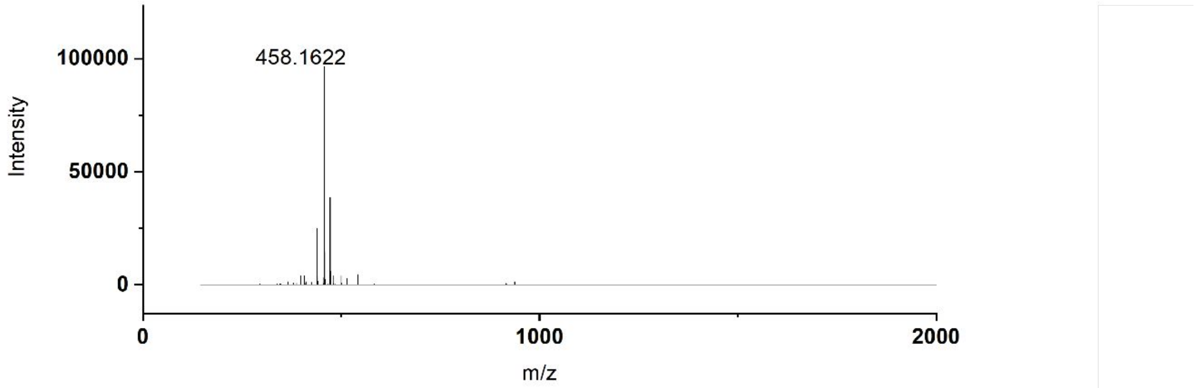

Figure S38. (A) Compound (**13**) (B) ^{1}H NMR (400 MHz) spectrum of compound (**13**) in CD_3OD (C) ^{13}C NMR spectrum of compound (**13**) in CD_3OD (D) HRMS (ESI+) of compound (**13**) calculation for $[C_{18}H_{29}NNaO_{11}]^+$ 458.16328; found 458.16229.

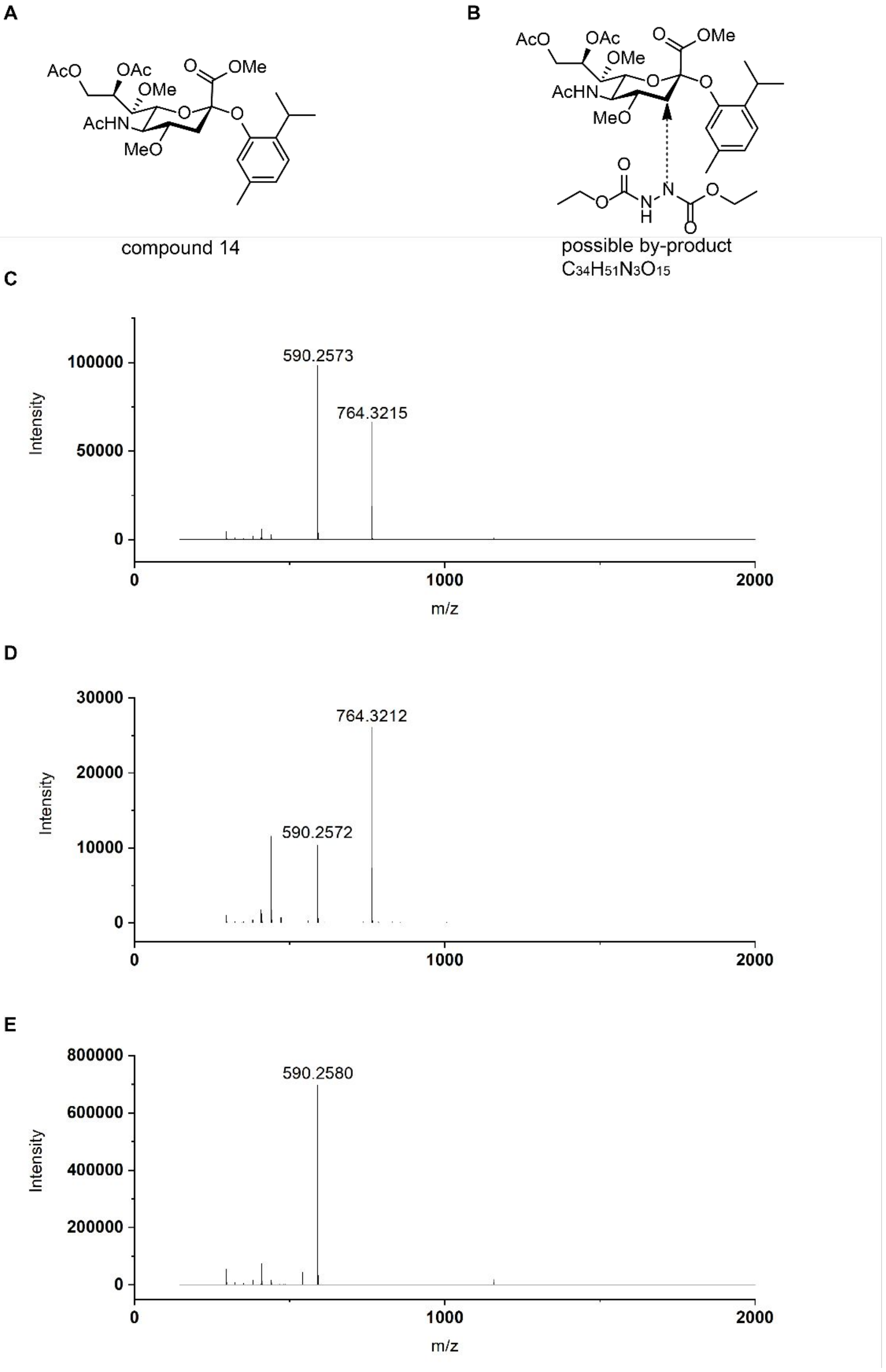

Figure S39. (A) Compound (**14**) (B) possible structure of the observed by-product. A possible interaction or binding site is shown by a dashed arrow (C) HRMS (ESI+) of compound (**14**) (charge 1 from approach (i)) calculation for $[C_{28}H_{41}NNaO_{11}]^+$ 590.25718; found 590.25738 and by-product calculation for $[C_{34}H_{51}N_3NaO_{15}]^+$ 764.32124 found 764.32157 (D) HRMS (ESI+) of compound (**14**) (charge 2 from approach (i)) calculation for $[C_{28}H_{41}NNaO_{11}]^+$ 590.25718; found 590.25720 and by-product calculation for $[C_{34}H_{51}N_3NaO_{15}]^+$ 764.32124 found 764.32127. (E) HRMS (ESI+) of compound (**14**) (from approach (ii)) calculation for $[C_{28}H_{41}NNaO_{11}]^+$ 590.25718; found 590.25807.

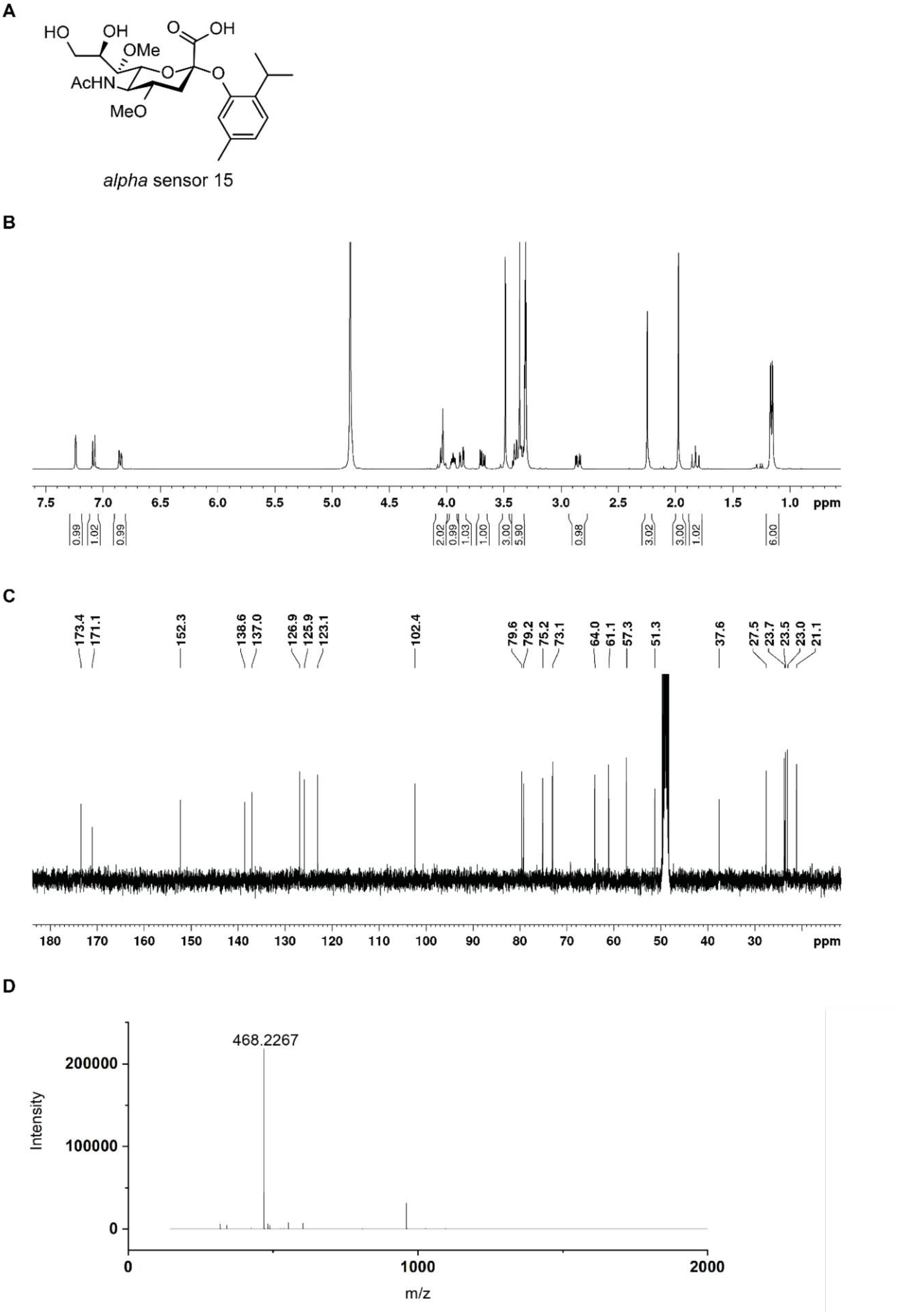

Figure S40. (A) α-sensor (**15**) (B) ^{1}H NMR (400 MHz) spectrum of α-sensor (**15**) in CD_3OD (C) ^{13}C NMR spectrum of α-sensor (**15**) in CD_3OD (D) HRMS (ESI-) of α-sensor (**15**) calculation for $[C_{23}H_{34}NO_9]^-$ 468.22391; found 468.22673 (all data shown from approach (ii)).

A

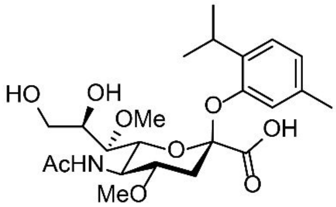

beta sensor 15

B

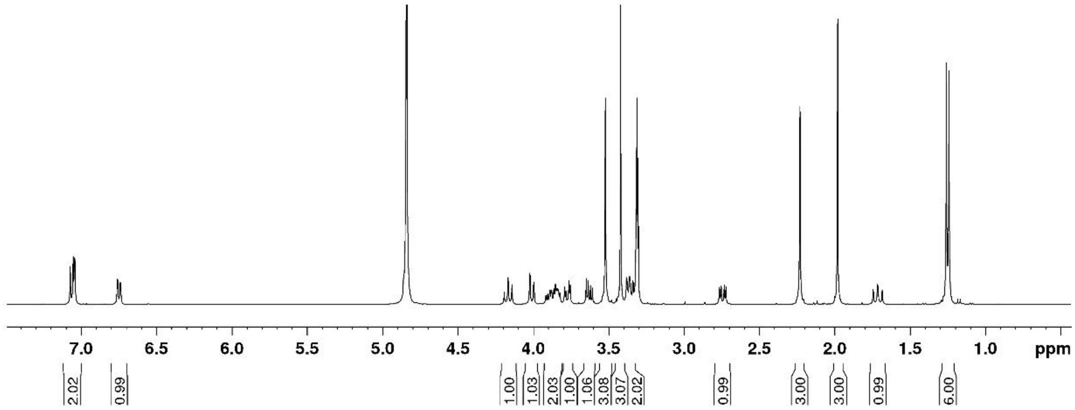

C

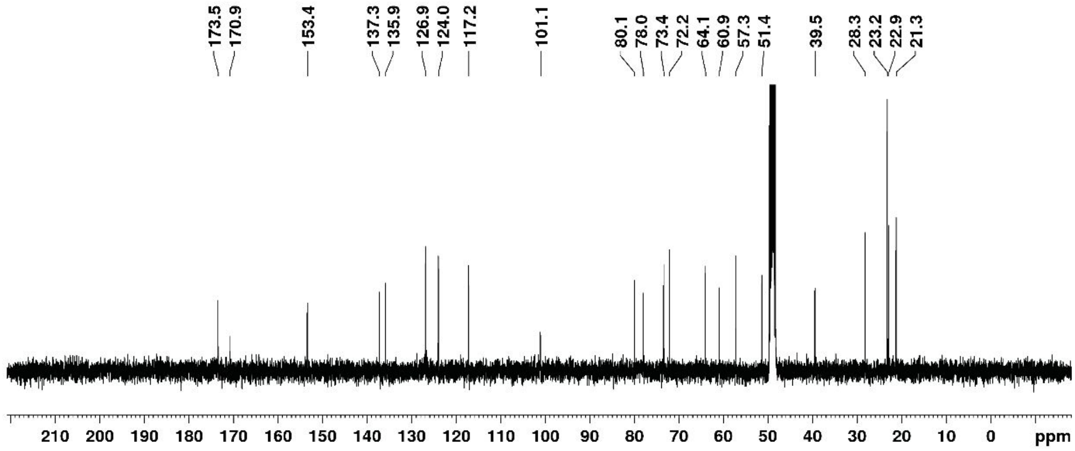

Figure S41. (A) β-sensor (**15**) (B) ^{1}H NMR (400 MHz) spectrum of beta sensor (**15**) in CD_3OD (C) ^{13}C NMR spectrum of β-sensor (**15**) in CD_3OD (all data shown from approach (ii)).

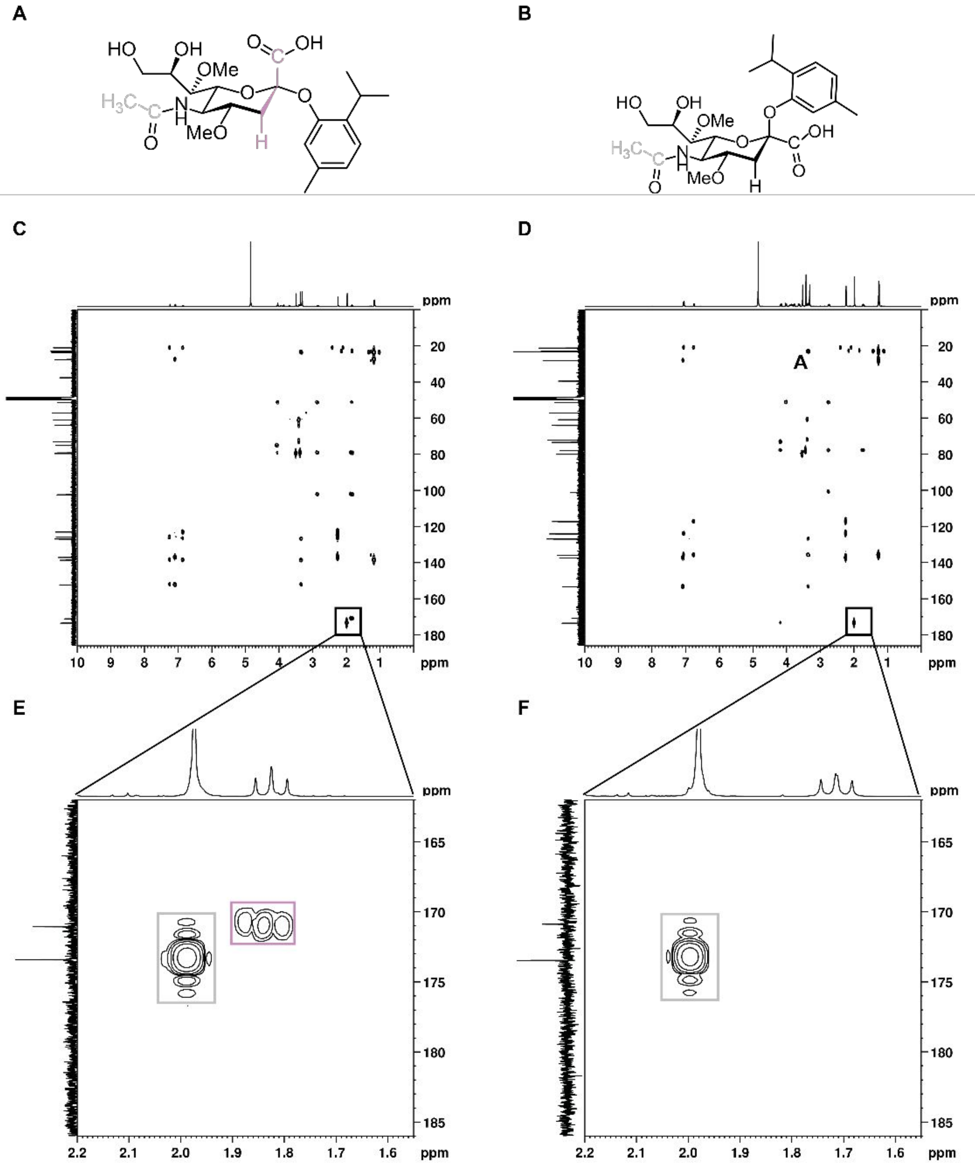

Figure S42. (A) α-sensor (**15**) and (B) β-sensor (**15**) with relevant motifs highlighted in grey and pink. HMBC spectra in CD_3OD of (C) α-sensor (**15**), and (D) β-sensor, as well as respective magnifications (E, F) of the HMBC spectra. The relevant coupling observed for the α-sensor (**15**) but not the β-sensor (**15**) is highlighted in pink, and identical couplings of both sensors are highlighted in grey (all data shown from approach (ii)).

A

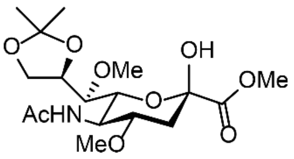

compound 16

B

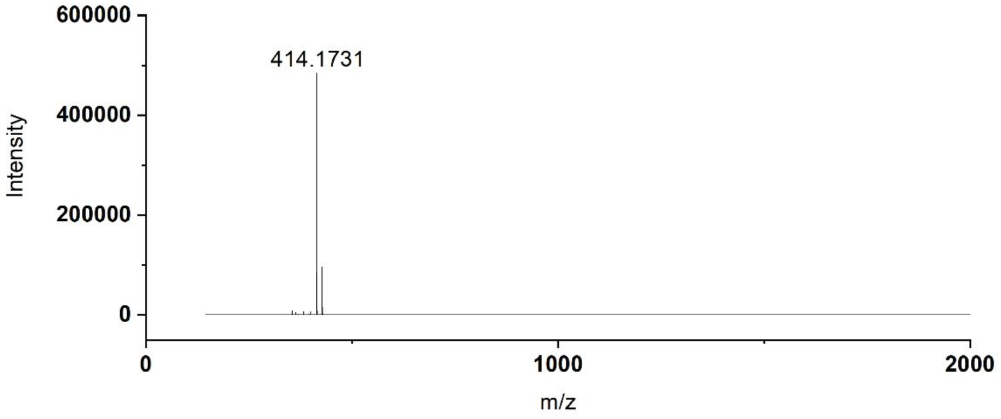

Figure S43. (A) compound (**16**) (B) HRMS (ESI+) of compound (**16**) calculation for $[C_{17}H_{29}NNaO_9]^+$ 414.17345; found 414.17310

A

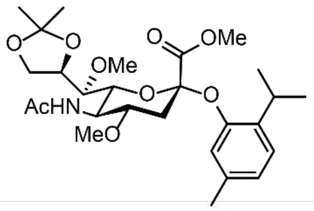

compound 17

B

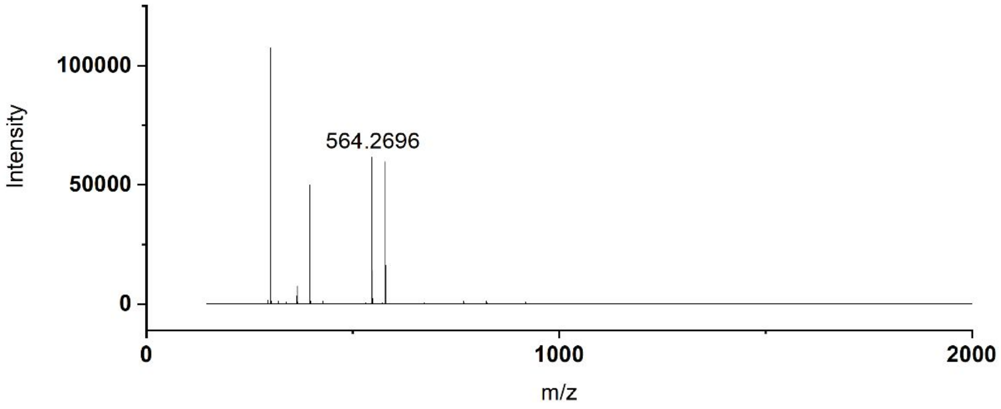

Figure S44. (A) compound (**17**) (B) HRMS (ESI+) of compound (**17**) calculation for $[C_{27}H_{41}NNaO_9]^+$ 546.26735; found 546.26965

Supporting References

(1) abcam. Neuraminidase Assay Kit (Fluorometric - Blue, ab138888). Neuraminidase Assay Kit (Fluorometric - Blue, ab138888): Cambridge, UK.
(2) Wilson III, C. W.; Shaw, P. E. Importance of thymol, methyl N-methylanthranilate, and monoterpene hydrocarbons to the aroma and flavor of mandarin cold-pressed oils. *Journal of Agricultural and Food Chemistry* **1981**, *29* (3), 494-496.
(3) Sánchez, L. M.; Ramos, M. J. G.; del Mar Gómez-Ramos, M.; Vazquez, P. P.; Flores, J. M. Presence, persistence and distribution of thymol in honeybees and beehive compartments by high resolution mass spectrometry. *Environmental Advances* **2021**, *5*, 100085.
(4) Özakar, R. S.; Özakar, E. Current overview of oral thin films. *Turkish journal of pharmaceutical sciences* **2021**, *18* (1), 111.
(5) Burdock, G. A. *Fenaroli's handbook of flavor ingredients*; CRC press, 2016.
(6) Meng, E. C.; Goddard, T. D.; Pettersen, E. F.; Couch, G. S.; Pearson, Z. J.; Morris, J. H.; Ferrin, T. E. UCSF ChimeraX: Tools for structure building and analysis. *Protein Science* **2023**, *32* (11), e4792.
(7) Case, D. A.; Aktulga, H. M.; Belfon, K.; Cerutti, D. S.; Cisneros, G. A.; Cruzeiro, V. W. D.; Forouzesh, N.; Giese, T. J.; Götz, A. W.; Gohlke, H.; et al. AmberTools. *Journal of Chemical Information and Modeling* **2023**, *63* (20), 6183-6191. DOI: 10.1021/acs.jcim.3c01153.
(8) Hanwell, M. D.; Curtis, D. E.; Lonie, D. C.; Vandermeersch, T.; Zurek, E.; Hutchison, G. R. Avogadro: an advanced semantic chemical editor, visualization, and analysis platform. *Journal of cheminformatics* **2012**, *4*, 1-17.
(9) McNutt, A. T.; Li, Y.; Meli, R.; Aggarwal, R.; Koes, D. R. GNINA 1.3: the next increment in molecular docking with deep learning. *J. Cheminf.* **2025**, *17* (1), 1–8. DOI: 10.1186/s13321-025-00973-x.
(10) Liav, A.; Hardgrave, R. F.; Blystone, S.; Turner, G. A.; Foundation, O. M. R. Synthesis of 4-alkoxy-n-acetylneuraminic acid. 1995.
(11) Liav, A.; Hansjergen, J. A.; Achyuthan, K. E.; Shimasaki, C. D. Synthesis of bromoindolyl 4,7-di-O-methyl-Neu5Ac: specificity toward influenza A and B viruses. *Carbohydr. Res.* **1999**, *317* (1), 198–203. DOI: 10.1016/s0008-6215(99)00058-0.
(12) Marra, A.; Sinay, P. Acetylation of N-Acetylneuraminic Acid and Its Methyl-Ester. *Carbohydrate research* **1989**, *190* (2), 317-322. DOI: Doi 10.1016/0008-6215(89)84135-7.
(13) Shelke, S. V.; Cutting, B.; Jiang, X. H.; Koliwer-Brandl, H.; Strasser, D. S.; Schwardt, O.; Kelm, S.; Ernst, B. A Fragment-Based In Situ Combinatorial Approach To Identify High-Affinity Ligands for Unknown Binding Sites. *Angew Chem Int Edit* **2010**, *49* (33), 5721-5725. DOI: 10.1002/anie.200907254.
(14) Kánya, N.; Kun, S.; Batta, G.; Somsák, L. Glycosylation with ulosonates under Mitsunobu conditions: scope and limitations. *New Journal of Chemistry* **2020**, *44* (34), 14463-14476. DOI: 10.1039/d0nj03044a.
(15) Šardzík, R.; Noble, G. T.; Weissenborn, M. J.; Martin, A.; Webb, S. J.; Flitsch, S. L. Preparation of aminoethyl glycosides for glycoconjugation. *Beilstein journal of organic chemistry* **2010**, *6* (1), 699-703.
(16) Yang, W.; Liu, X.; Peng, X.; Li, P.; Wang, T.; Tai, G.; Li, X. J.; Zhou, Y. Synthesis of novel N-acetylneuraminic acid derivatives as substrates for rapid detection of influenza virus neuraminidase. *Carbohydrate research* **2012**, *359*, 92-96.
(17) Miller, M. B. Clinical Impact of Rapid Point-of-Care PCR Influenza Testing in an Urgent Care Setting: a Single-Center Study. *J. Clin. Microbiol.* **2019**.
(18) Ryu, S. W.; Suh, I. B.; Ryu, S.-M.; Shin, K. S.; Kim, H.-S.; Kim, J.; Uh, Y.; Yoon, K. J.; Lee, J.-H. Comparison of three rapid influenza diagnostic tests with digital readout systems and one conventional rapid influenza diagnostic test. *J. Clin. Lab. Anal.* **2018**, *32* (2), e22234. DOI: 10.1002/jcla.22234.

(19) Trombetta, C. M.; Perini, D.; Mather, S.; Temperton, N.; Montomoli, E. Overview of Serological Techniques for Influenza Vaccine Evaluation: Past, Present and Future. *Vaccines* **2014**, *2* (4), 707–734. DOI: 10.3390/vaccines2040707.
(20) Tabata, K. V.; Minagawa, Y.; Kawaguchi, Y.; Ono, M.; Moriizumi, Y.; Yamayoshi, S.; Fujioka, Y.; Ohba, Y.; Kawaoka, Y.; Noji, H. Antibody-free digital influenza virus counting based on neuraminidase activity. *Sci. Rep.* **2019**, *9* (1067), 1–13. DOI: 10.1038/s41598-018-37994-6.
(21) Phetcharakupt, V.; Pasomsub, E.; Kiertiburanakul, S. Clinical manifestations of influenza and performance of rapid influenza diagnostic test: A university hospital setting. *Health Sci. Rep.* **2021**, *4* (4), e408. DOI: 10.1002/hsr2.408.
(22) Gavin, P. J.; Thomson, R. B. Review of Rapid Diagnostic Tests for Influenza. *Clinical and Applied Immunology Reviews* **2004**, *4* (3), 151–172. DOI: 10.1016/s1529-1049(03)00064-3.
(23) Abraham, M. K.; Perkins, J.; Vilke, G. M.; Coyne, C. J. Influenza in the Emergency Department: Vaccination, Diagnosis, and Treatment: Clinical Practice Paper Approved by American Academy of Emergency Medicine Clinical Guidelines Committee. *J. Emerg. Med.* **2016**, *50* (3), 536–542. DOI: 10.1016/j.jemermed.2015.10.013.
(24) Lin, X.; Liu, X.-Y.; Zhang, B.; Qin, A.-Q.; Hui, K.-M.; Shi, K.; Liu, Y.; Gabriel, D.; Li, X. J. A rapid influenza diagnostic test based on detection of viral neuraminidase activity. *Sci. Rep.* **2022**, *12* (505), 1–8. DOI: 10.1038/s41598-021-04538-4.
(25) van Elden, L. J. R.; Nijhuis, M.; Schipper, P.; Schuurman, R.; van Loon, A. M. Simultaneous Detection of Influenza Viruses A and B Using Real-Time Quantitative PCR. *J. Clin. Microbiol.* **2001**.
(26) Uyeki, T. M.; Bernstein, H. H.; Bradley, J. S.; Englund, J. A.; File, T. M.; Fry, A. M.; Gravenstein, S.; Hayden, F. G.; Harper, S. A.; Hirshon, J. M.; et al. Clinical Practice Guidelines by the Infectious Diseases Society of America: 2018 Update on Diagnosis, Treatment, Chemoprophylaxis, and Institutional Outbreak Management of Seasonal Influenza. *Clin. Infect. Dis.* **2019**, *68* (6), 895–902. DOI: 10.1093/cid/ciy874.
(27) Steininger, C.; Kundi, M.; Aberle, S. W.; Aberle, J. H.; Popow-Kraupp, T. Effectiveness of Reverse Transcription-PCR, Virus Isolation, and Enzyme-Linked Immunosorbent Assay for Diagnosis of Influenza A Virus Infection in Different Age Groups. *J. Clin. Microbiol.* **2002**.
(28) Prevention, C. f. D. C. a. Rapid Influenza Diagnostic Tests. 2024.
(29) Chu, H. Y.; Englund, J. A.; Huang, D.; Scott, E.; Chan, J. D.; Jain, R.; Pottinger, P. S.; Lynch, J. B.; Dellit, T. H.; Jerome, K. R.; et al. Impact of rapid influenza PCR testing on hospitalization and antiviral use: A retrospective cohort study. *J. Med. Virol.* **2015**, *87* (12), 2021–2026. DOI: 10.1002/jmv.24279.
(30) Diel, R.; Nienhaus, A. Rapid Point-of-Care Influenza Testing for Patients in German Emergency Rooms – A Cost-Benefit Analysis. *JHEOR* **2019**, *6* (3), 203–212. DOI: 10.36469/001c.11206.
(31) Achyuthan, K. E.; Pence, L. M.; Appleman, J. R.; Shimasaki, C. D. ZstatFlu®-II test: a chemiluminescent neuraminidase assay for influenza viral diagnostics. *Luminescence* **2003**, *18* (3), 131–139. DOI: 10.1002/bio.714.
(32) Hsiao, Y.-S.; Parker, D.; Ratner, A. J.; Prince, A.; Tong, L. Crystal structures of respiratory pathogen neuraminidases. *Biochem. Biophys. Res. Commun.* **2009**, *380* (3), 467–471. DOI: 10.1016/j.bbrc.2009.01.108.
(33) Jumper, J.; Evans, R.; Pritzel, A.; Green, T.; Figurnov, M.; Ronneberger, O.; Tunyasuvunakool, K.; Bates, R.; Žídek, A.; Potapenko, A.; et al. Highly accurate protein structure prediction with AlphaFold. *Nature* **2021**, *596*, 583–589. DOI: 10.1038/s41586-021-03819-2.
(34) Chavas, L. M. G.; Tringali, C.; Fusi, P.; Venerando, B.; Tettamanti, G.; Kato, R.; Monti, E.; Wakatsuki, S. Crystal Structure of the Human Cytosolic Sialidase Neu2: EVIDENCE FOR THE DYNAMIC NATURE OF SUBSTRATE RECOGNITION*. *J. Biol. Chem.* **2005**, *280* (1), 469–475. DOI: 10.1074/jbc.M411506200.
(35) Gaskell, A.; Crennell, S.; Taylor, G. The three domains of a bacterial sialidase: a β-propeller, an immunoglobulin module and a galactose-binding jelly-roll. *Structure* **1995**, *3* (11), 1197-1205. DOI: 10.1016/S0969-2126(01)00255-6 (acccessed 2024/12/18).

(36) Lei, R.; Hernandez Garcia, A.; Tan, T. J. C.; Teo, Q. W.; Wang, Y.; Zhang, X.; Luo, S.; Nair, S. K.; Peng, J.; Wu, N. C. Mutational fitness landscape of human influenza H3N2 neuraminidase. *Cell Rep.* **2023**, *42* (1), 111951. DOI: 10.1016/j.celrep.2022.111951.
